# Deep learning four decades of human migration

**DOI:** 10.1038/s41586-026-10611-7

**Published:** 2026-06-10

**Authors:** Thomas Gaskin, Guy J. Abel

**Affiliations:** 1 Department of Methodology, London School of Economics and Political Science, London, UK; 2 Department of Applied Mathematics and Theoretical Physics, University of Cambridge, Cambridge, UK; 3 Department of Sociology and Research Hub of Population Studies, The University of Hong Kong, Hong Kong SAR, China; 4 International Institute for Applied Systems Analysis, Laxenburg, Austria

**Keywords:** Sociology, Computational science

## Abstract

Human migration is a fundamental driver of global demographic change, shaping population structure, labour markets and social policy across countries^1–3^. Although long-term migration patterns are often linked to economic development^4^, they can shift rapidly in response to shocks such as conflict, environmental crises and political change^5^. Despite its importance, migration remains difficult to measure consistently: existing data are sparse, concentrated in high-income settings and are fragmented across incompatible definitions, temporal resolutions and data types^6–8^. Past efforts have relied on partial datasets, including flow records, stock estimates and model-based reconstructions with limited coverage^9–14^. A central challenge is therefore to construct a globally consistent, high-resolution account of migration flows over time. Here we present a new dataset of annual origin-destination migration across 230 countries and regions from 1990 to the present, integrating diverse data sources into a unified modelling framework. By combining official statistics, census-based stocks, net migration estimates and past flow reconstructions, our approach produces temporally detailed and spatially comprehensive estimates that substantially extend existing resources. Using an ensemble of deep recurrent neural networks informed by geographic, economic, cultural and political covariates, we capture both persistent trends and short-term responses to changing conditions—all while propagating uncertainty to generate confidence bounds. Our results outperform existing five-year flow estimates on held-out data and provide finer temporal resolution, revealing previously obscured dynamics in global migration patterns. This framework highlights regions in which uncertainty remains high and data collection is most urgently needed. By releasing all data, code and trained models, we provide a transparent and reproducible foundation for future work. These advances enable a more timely and detailed understanding of human mobility, with implications for research and policy in an increasingly dynamic global system.

## Abstract (web summary)

A global annual migration-flow dataset (1990–2024) is produced using deep-learning models and diverse sources to estimate movements across 230 countries with improved temporal resolution, coverage and uncertainty estimates.

## Main

The movement of people—within countries and between them—is an important topic across multiple domains. Migration drives demographic change, shaping the size and composition of populations; it can influence labour markets^1^, inform social policy^2^ and is a popular topic for public debate^3^. Although migration often follows long-term trends driven by development^4^, it can be dramatically altered by short-term shocks—armed conflict, famine, natural disasters, political instability, changes in national borders, peace agreements or independence movements^5^.

**Fig. 1:**
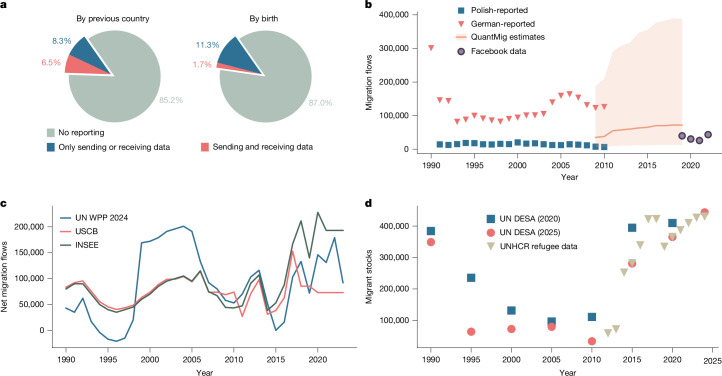
Availability of flow data across global migration corridors. **a**, Fraction of corridors that have reported flow values in the 1990–2020 period by any of the validation datasets used in this work^7,66–69^. Statistics for both origin- and birth-destination corridors are shown; these are further disaggregated by corridors for which neither, only one of, or both the sending and the receiving country has reported figures. **b**, Migration flow estimates based on domicile registration (usually with a local authority) are available for a small number of countries, but the discrepancies can be large: estimates of flows—based on registrations of people arriving from Poland as reported by German authorities (red) and de-registrations of people leaving Poland for Germany, as reported by Polish authorities (blue)^7^—are shown. The harmonized QuantMig estimates (orange; error bands show the 97.5% quantile) and the recent digital-trace estimates based on Facebook data are also shown. **c**, Various estimates of the net migration for France, such as those from the UN Population Division’s World Population Prospects (2024 Revision), the US Census Bureau (USCB)’s International Dataset^70^, and the French National Institute of Statistics and Economic Studies (INSEE)^71^. **d**, UN DESA estimates of the migrant stock of Somalians in Ethiopia, which do not agree across revisions. In some cases, they are based on refugee data figures from the UNHCR^72^.

Human migration, however, is notoriously difficult to define and track^6^. Current analyses of global migration systems rely heavily on migrant population data published at five-year intervals by the United Nations (UN) and at ten-year intervals by the World Bank. These datasets provide counts of migrants in each country by country of birth, typically referred to as stock data. Although relatively straightforward to collect, they offer only a snapshot at a fixed point in time and provide limited insight into the temporal dynamics of migration: migrants may have arrived immediately before the observation point or several decades earlier. To better capture migration dynamics, researchers have developed methods that estimate migration flows over multi-year periods by comparing changes in migrant stocks at the beginning and end of each interval^9^. However, as these estimates are tied to gaps in the underlying stock data, the resulting five- or ten-year estimates inevitably smooth or completely miss movements that occur in the intervening years. What researchers on global migration ideally need are annual flow data for all countries. Such data would allow them to track the tempo of migration systems with far greater precision, integrate migration patterns with other annually reported datasets on drivers such as economic change, conflict, climate or policy reforms, feed into annual population projection models, and facilitate both causal and comparative analyses across countries and regions. Yet existing annual migration flow data are predominantly available only from high-income Western countries with the statistical infrastructure to monitor migration. Such data only cover a small share of the global migration system^7,8^ (Fig. 1a) and reinforce a receiving-country bias in global migration research^15^.

In countries in which migration flow statistics are published, the definitions of what constitutes a migration event are determined by criteria designed to meet domestic policy needs^16,17^, which can bias comparative analyses. Although the UN recommends a twelve-month threshold^18^, where anyone relocating for the majority of a year or more qualifies as a migrant, this definition is not applied consistently. Some countries such as Germany mandate residential registration, requiring migrants to report their country of origin upon arrival. Others, such as the UK, rely on visa records, administrative data and, until recently, passenger surveys. A third common approach uses border entry statistics collected by immigration authorities. Each method has limitations: registration systems typically undercount emigration, as few individuals de-register when leaving; passenger surveys and border data are not comprehensive and may conflate short- and long-term travellers. As a result, estimates from sending and receiving countries often diverge markedly. For instance, in 2005 Germany reported 160,000 arrivals from Poland, whereas Poland recorded only 12,300 departures to Germany (Fig. 1b). In Europe, to reconcile such discrepancies, statistical demographers have developed models to estimate bilateral migration flows between countries. The most recent study, the QuantMig project^10,11^, made use of a Bayesian framework alongside expert insights to estimate bilateral migration flows for 30 European countries from 2009 to 2019. This produced a harmonized dataset, revealing substantial uncertainty—in some cases, with credible intervals spanning over 100%. Given the dearth of migration flow statistics available to monitor many major migration corridors between developing countries, this approach does not easily generalize to a global environment. Labour migration data represent another important source^19^, as migrant workers often make up a substantial share of international movers. However, here too definitions and data standards vary widely between countries^20^, and undocumented migration—by its very nature—remains largely invisible to official statistics.

A recent study attempted to bypass official data sources for monitoring global migration flows by analysing digital traces^21^. By monitoring changes in aggregated, anonymized monthly Facebook location data to estimate bilateral flows among 181 countries between 2019 and 2022, the study captured, for example, the displacement of Ukrainians following the Russian invasion, the Venezuelan migration crisis and altered migration patterns during the pandemic. The digital traces from more than three billion users were weighted to represent population-level migration flows, accounting for differences in Facebook usage and economic development along each corridor, and calibrated against official migration statistics in selected countries. These data provide, for the first time, a near-global direct estimate of migration flows.

One macroindicator that many countries are interested in estimating is the net migration—that is, the balance of immigration and emigration. A small number of countries publish net migration figures, estimated from immigration and border statistics (Supplementary Fig. 1), whereas on a global scale, the UN Department of Economic and Social Affairs (UN DESA) provides figures from 1950 onwards in its World Population Prospects (WPP) reports. These are primarily based on demographic estimates rather than migration statistics. As births and deaths are more widely and consistently tracked than migration figures, in principle the net migration can be estimated by subtracting the natural change (births minus deaths) from the total population change. Although this approach is theoretically sound, in practice it is hindered by irregularities in measuring the total population and its change over time, which are sensitive to inconsistencies in census methodology. Consequently, demographic net migration estimates can differ noticeably from migration-based statistics, even for countries whose population data are of high quality (Fig. 1c).

**Extended Data Fig. 1:**
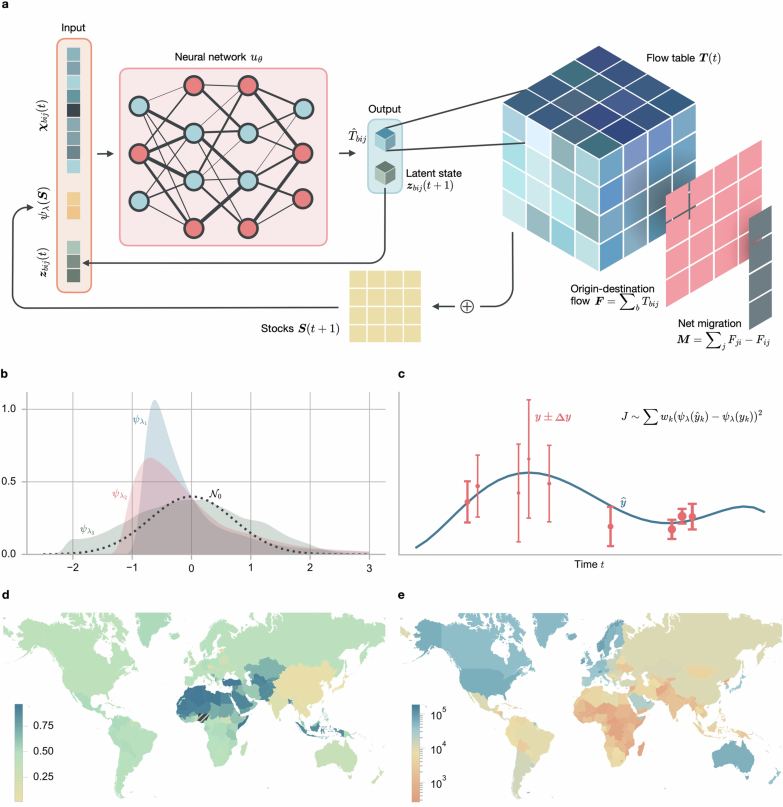
Overview of the training method. **a** For each edge, the covariates ***χ***_*bij*_, the migrant stock (*S*_*bi*_, *S*_*bj*_), and the latent state ***z***_*bij*_ are passed through the neural network *u*_*θ*_ to produce an estimated flow ${\widehat{T}}_{bij}$ and an updated latent variable ***z***_*bij*_(*t* + 1). The complete flow table ***T*** is assembled from all the individual estimates and used to calculate the net migration ***M***, the origin-destination flow ***F***, and the migrant stock ***S***(*t* + 1) of the next year, which is then fed back into the neural network as input. This gives a recursive training procedure. **b** Input and target data are transformed to more closely resemble a normal distribution (dashed line) using a Yeo-Johnson type transformation (eq. (8)). This is done by tuning the parameter *λ* using maximum likelihood estimation. **c** The training loss *J* is calculated from the residuals of the prediction and the observation data. Each term is weighted by the uncertainty on the target value, with smaller uncertainties leading to a higher weight (represented by a thicker bar). **d**/**e** Two covariates used to train the model. The bilateral religious similarity score for Nigeria (**d**), measuring the overlap in adherents of major religions with other countries, and real GDP per capita, in constant 2015 USD, as of 2019 (**e**).

Here we combine deep learning with a mechanistic flow model to estimate annual bilateral migration flows in the 1990–2023 period between all 230 countries and regions recognized by the UN. Our data are disaggregated by country of birth, meaning that, aside from the flows and the net migration for each country, we also obtain a complete dataset of annual migrant stocks, that is, the number of migrants *S*_*bj*_(*t*) born in country *b* residing in country *j* in year *t*. A deep neural network is trained on an extensive set of socio-economic and cultural covariates for each country (Extended Data Table 1), allowing us to disentangle the drivers of migration and opening the door to future forecasting of migration flows. The network is trained to match a set of target data, comprising the UN DESA migrant stocks^22^, Facebook data, as well as a small number of predominantly European bilateral flows and net migration data. The target data are used to construct a loss function, which is iteratively minimized during training^23,24^. The loss function quantifies the mismatch between predictions and targets, and is an objective that the neural network seeks to minimize by following the loss gradient, or direction of steepest descent. Once trained, the neural network acts as a function mapping input covariates to migration flows (Extended Data Fig. 1). By training a family of neural networks and further ‘pushing’ the uncertainty on the input data through the network, our approach also enables uncertainty quantification, allowing us to pinpoint the countries in which data are inconsistent and collection should be improved.

This marks a paradigm shift for the computational toolset hitherto used to model global migration. Most past techniques have relied solely on migrant stock data published by UN DESA, which provides estimates at five-year intervals from 1990 (Fig. 1d). The simplest estimation techniques are based on stock differencing^9^ and assume that the bilateral flow *F*_*ij*_ is equal to the difference in stocks *S*_*bj*_(*t* + 1) − *S*_*bj*_(*t*) with *b* = *i*. Negative differences are either dropped (meaning zero flow)^25,26^ or counted towards flows in the opposite direction^27^. The simplifying assumption here is that bilateral migration flows only take place from a person’s country of birth to a destination; that is, the stock of Swedes in the UK changes only due to Swedish people arriving from and returning to Sweden; but not due to Swedish people arriving from, say, Norway. To account for this, a more sophisticated array of so-called demographic accounting methods were proposed^12–14^. These attempt to infer a three-dimensional flow matrix *T*_*bij*_, with each entry modelling the flow of people born in *b* moving from *i* to *j*, allowing for greater flexibility, but also greatly increasing the number of parameters to be estimated. The flow table is constrained such that its estimates reproduce the stock differences. These are typically first adjusted to account for births and deaths, whereby the estimated flow reproduces only the change in stocks not caused by demographic change.

Stock-based flow estimation approaches all take the stock data at face value; they are also unable to increase the temporal resolution of the estimates, and have thus far only yielded five- or ten-year flows (the resolution of the UN DESA or World Bank data). An alternative is the use of gravity models^28^, broadly taken to refer to any type of regression-based approach that relates the flow to a set of covariates *χ*. These models can, in principle, capture flows at any resolution, provided the covariates are of sufficient quality and are suitably chosen; however, they tend to perform poorly when modelling migration^29^, even with a large and sophisticated set of covariates. The fundamental problem when modelling migration as

$$\log {T}_{bij}(t)=f({\chi }_{bij}(t))$$

is that it represents humans as Markovian, acting only on the basis of the current state of the world with no regard to the past. This may be warranted when considering the response to a sudden, cataclysmic event, but is hardly reasonable when incorporating long-term, macro-level political, economic or social indicators. The decision to leave is, in most cases, not merely predicated on the current economic climate: crises from past years can influence a person’s decision, due to a multitude of delayed effects and complex feedback loops. Any model that does not account for the system’s memory will thus fail to accurately reproduce, let alone explain, the temporal and spatial variance in human migration. Here we use a recurrent neural network^30,31^, which implements a form of ‘memory’ by maintaining a ‘hidden’ or ‘latent’ state ***z***(*t*) that changes over time. This allows the network to selectively retain past information using a dynamic filter and learn temporal correlation patterns of varying length. The latent state incorporates past dynamics to inform the flows of today without assuming temporal stationarity in migration flows, which are typically unstable^32^.

In recent years there has been a steep increase in the application of machine learning methods to predict and explain human migration and mobility patterns^33–35^. Studies have applied machine learning methods, including deep learning approaches, in a multitude of settings. Most applications have been developed to address commuting and mobility patterns within cities, regions and countries^36–40^. Modelling efforts in migration research have largely focused on internal moves within countries^41–43^, including analyses of climatic and environmental drivers of mobility^44–47^, as well as forecasting asylum seeking and irregular international migration into predominantly high-income Western states^48,49^. Unlike in the global migration data setting, movement response variables in this recent literature have been derived from a single source, where the challenges of combining measures and the problems of missing or inconsistent data across multiple origin-destination corridors are absent. Furthermore, rather than quantifying the scale and patterns of international migration at the global level, the focus of these studies has been on providing superior extrapolatory predictions to classic modelling approaches or on helping detect possible linkages between covariate factors and mobility or migration in data-rich settings.

The article is structured as follows: first, we present the estimation results, showcasing the data on a selection of case studies. We validate our method’s performance on test data of unseen flows and compare it with a selection of standard methods discussed above. The inference method is presented in detail in the Methods. We denote the stock estimates as *S*, the flows disaggregated by birth as *T*, the total origin-destination flows as *F* and the net migration as **M**. For notational clarity, we will omit the time argument wherever possible. Estimated quantities will be denoted by a hat, for example, $\hat{{\bf{M}}}$.

## A global map of migration

**Fig. 2:**
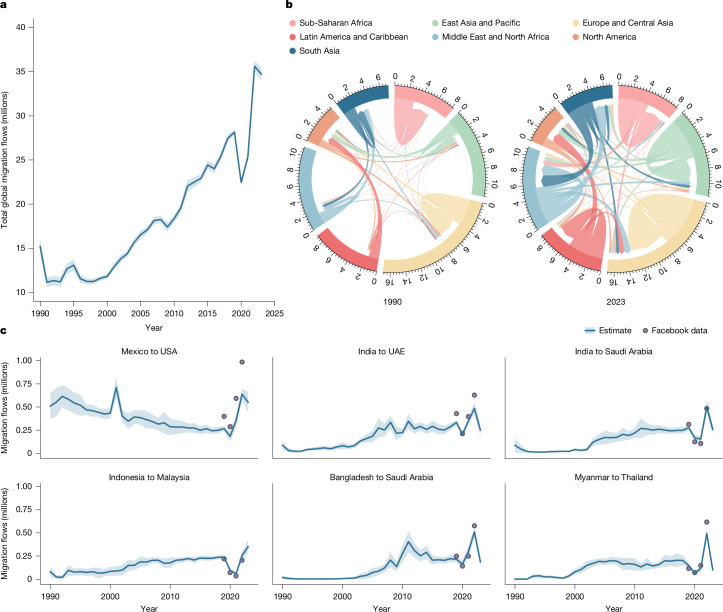
Global bilateral, annual migration flows, disaggregated by country of birth, for all countries and territories from 1990 to 2023. Error bands indicate the mean and one s.d. over *n* = 1,500 samples from the neural network ensemble. Regions in this and the following panels have been selected to cover a diversity of country sizes, income levels and geographies. **a**, Total global flows, in millions. The increase cannot be explained by the rising global population, as the per-capita figures show a similar trend (Extended Data Figs. 2 and 3). **b**, Chord diagrams of regional flow patterns for 1990 and 2023, in millions. The arrow head indicates the direction of the estimated migration flow. The width of the arrow at its base indicates the size of the migrant flows. Numbers on the outer section axis indicate the size of the migration flows, in millions. The axes are fixed on the scale of the sum of the regional immigration and emigration flows in 2023 for direct comparisons between years. Colours correspond to the countries’ region of origin. **c**, The six largest country-level flow corridors of the past 35 years, measured by total flow in millions. Facebook data are also shown.

**Extended Data Fig. 2:**
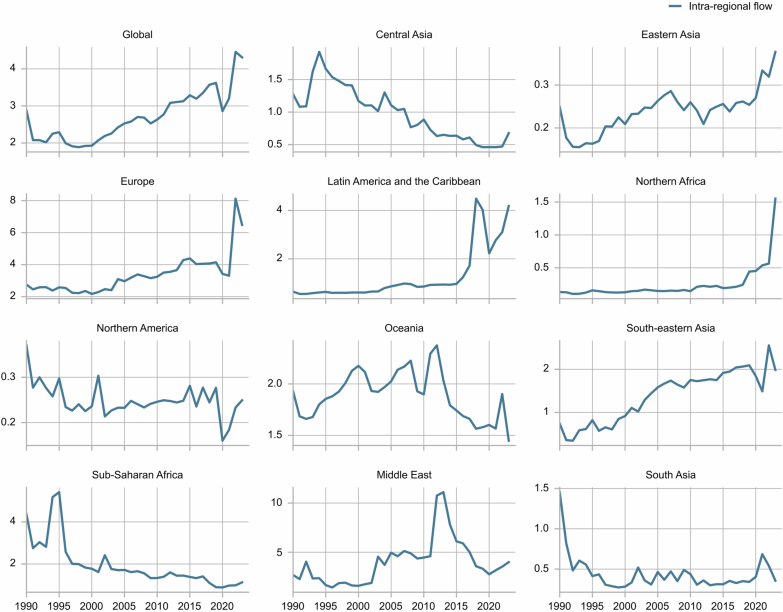
Per-capita intra-regional migration flows. Values are per 1000 population. Also shown are the total global flows (top left). The trend matches that shown in Fig. 2a in the main manuscript.

Our estimates reveal that, since 2000, global migration movements have risen from 13 million people annually to around 35 million in 2023 (Fig. 2a). This trend is not explained by a rising global population, as per-capita migration saw a similarly steady increase from 0.2% in 2000 to 0.45% in 2023 (Extended Data Fig. 2). Since the turn of the millennium, total global migration has only seen two periods of sustained decrease: during the Great Recession in 2008 to 2009, and during the COVID-19 pandemic in 2020. The largest single-year event we registered is the 1994 movement of people from Rwanda to the Democratic Republic of the Congo, totalling almost 950,000. Globally, the Middle East experienced the highest total inflow of migrants, chiefly from South Asia and the Philippines, with immigration from Bangladesh to Saudi Arabia alone averaging around 300,000 people per year from 2010 onwards (Fig. 2c). We estimate that, since 2010, a total of 19 million people, averaging 1.35 million per year, migrated from India, Pakistan and Bangladesh to Saudi Arabia, Qatar, Bahrain and the UAE—this compares to 13.6 million movements from Mexico to the USA over the entire period since 1990.

**Fig. 3:**
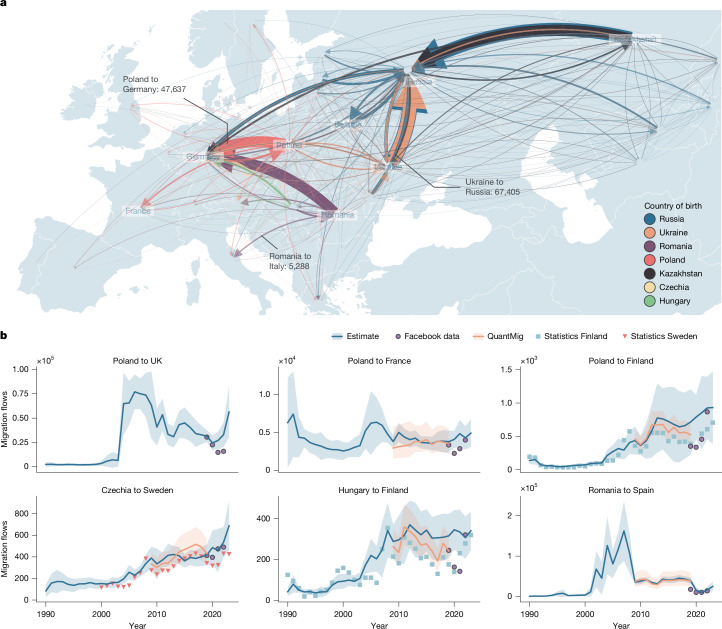
Migration in Europe. **a**, Intra-European flows in 1991, colour-coded by country of birth. Some reference flows are indicated for scale. **b**, Total bilateral flows for selected European corridors. The estimates from the various target datasets used to train the model are also shown (Methods). Error bands represent the mean and one s.d. over *n* = 1,500 samples from the neural network ensemble.

**Extended Data Fig. 3:**
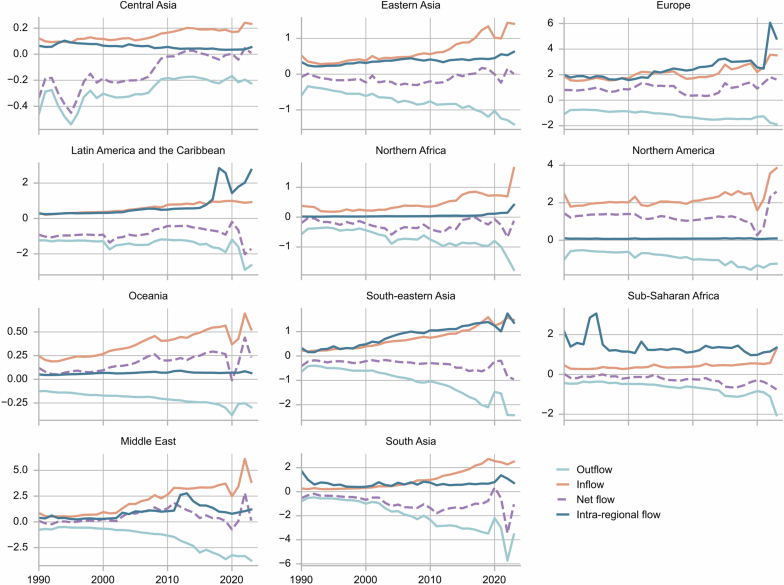
Region-specific migration flows. Inflows, outflows, and net flows are shown in millions.

Europe consistently ranks as the region with the highest volume of intraregional migration, surpassed only once by sub-Saharan Africa in the early 1990s during the Rwandan civil war (Extended Data Fig. 3). Pre-2020, gross flows in Europe reached around three million people annually, having steadily increased during the 2000s and 2010s following the eastward expansion of the EU and the Schengen region. Flows from Eastern to Western Europe since 1990 total around 20 million, or 600,000 per year. Figure 3 shows a snapshot of intra-European flows in 1991, following the collapse of the Soviet Union, colour-coded by country of birth. In that year, by our estimates, intra-European flows reached about 2.02 million people, of which 807,000 alone were of people born in Poland, Russia, Ukraine and Romania. The largest movements took place between Ukraine and Russia, Kazakhstan and Russia, and into Germany. During this time, we see high levels of return migration (bidirectional movement), as some sought to return to their country of birth, whereas others relocated abroad in search of economic opportunity. Figure 3b shows the flow estimates $\hat{F}$ for a selection of corridors, alongside values from the various datasets used to train the neural network. Our estimates match not only the data, but also the uncertainty on the QuantMig values exceedingly well (refer below the discussion on uncertainty quantification).

## Migration in the Global South

**Fig. 4:**
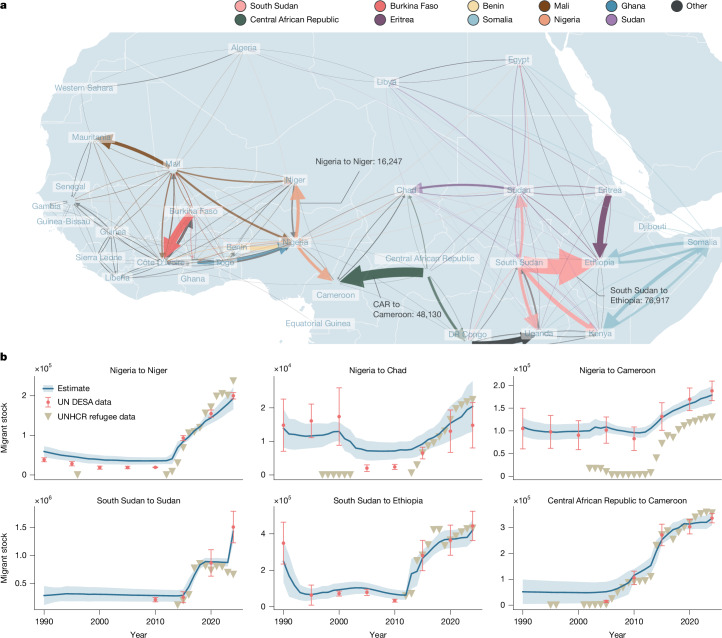
Migration in sub-Saharan Africa. **a**, Flows in 2014, colour-coded by country of birth. Some reference flows are indicated for scale. **b**, Migrant stocks for selected country pairs. Error bands indicate the mean and one s.d. over *n* = 1,500 samples from the neural network ensemble. Refugee figures from the UNHCR, and UN DESA stock data with estimated uncertainties, are also shown (Supplementary Fig. 3).

Europe is perhaps the region with the least need for a detailed analysis of migratory patterns, given that data are (relatively) plentiful. The value of our dataset lies primarily in what it tells us about movements in other parts of the world, especially the Global South. In the mid-2010s, for instance, sub-Saharan Africa saw several large-scale migration events. Civil war raged in the newly independent country of South Sudan from 2013 onwards, causing a large exodus into neighbouring Ethiopia (Fig. 4). The UN High Commissioner for Refugees (UNHCR) classifies the entire migrant population of South Sudanese in Ethiopia as refugees. Violence also erupted in West Africa, with the jihadist group Boko Haram starting an armed insurgency against the Nigerian government in 2009, and dramatically escalating its attacks in 2014, including by abducting nearly 300 young women from a school^50,51^. In 2013 to 2014 alone, we estimate that around 79,000 persons born in Nigeria moved or fled to neighbouring Chad, Niger, Cameroon—the majority of whom moved (45,000) to Niger. From 2009 to 2019, we estimate an outflow of Nigerian-born persons to these three countries of 250,000 with a s.d. of 31,000. This figure is dwarfed by the International Organization for Migration (IOM) estimate of around 2.4 million internally displaced people as a consequence of the violence^52^. Meanwhile, the ongoing civil war in the Central African Republic led to a continuous outflow to neighbouring Cameroon, Democratic Republic of the Congo and Chad.

## Revising the UN figures

**Fig. 5:**
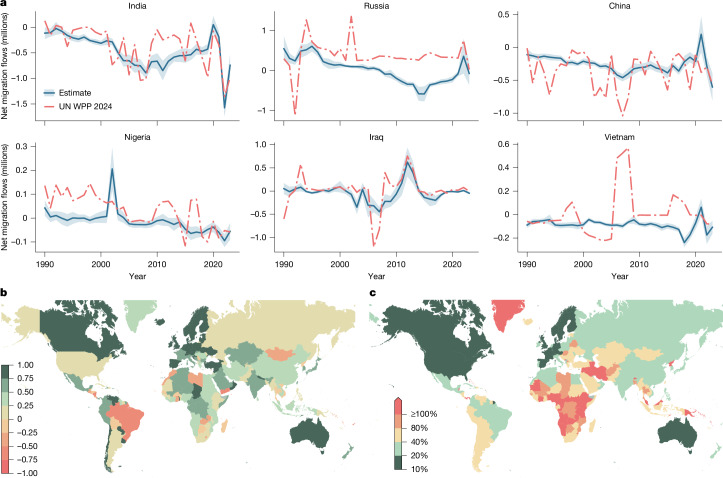
Net migration estimates and comparison with UN WPP data. **a**, Net migration figures for selected countries, alongside the WPP estimate, in millions. Error bands represent the mean and one s.d. over *n* = 1,500 samples from the neural network ensemble. **b**, Correlation coefficient of our estimates with WPP figures. **c**, Median relative uncertainty (s.d. over mean estimate) on our estimates.

In Fig. 5a we show the net migration figures $\hat{{\bf{M}}}$ for selected countries alongside the estimates **M**^WPP^ from the 2024 WPP report^53^. Our dataset provides a valuable correction to these figures, which, as mentioned in the introduction, are calculated from demographic residuals rather than migration statistics:

$${{\bf{M}}}^{{\rm{W}}{\rm{P}}{\rm{P}}}(t)=\Delta {\bf{P}}(t)-({\boldsymbol{\beta }}(t)-{\boldsymbol{\gamma }}(t)){\bf{P}}(t),$$

with **P**(*t*) the total population, and **β** and **γ** the crude birth and death rates, respectively. The variation in the WPP figures is often caused by anomalies in the population figures, which strongly affect the change in population Δ**P** and cause, for instance, Vietnam’s net migration to spike at approximately 2008, only to then fall back to zero in 2010. Although the UN figures would suggest positive migrant inflow to Russia since 1995, our estimates show that, in fact, Russian net migration turned negative around 2005—a trend only reversed by the displacement of Ukrainians in 2022.

## Meaningful uncertainty quantification

In Fig. 5b we show the correlation between our net migration figures and the most recent WPP estimates^53^. We see a strong positive correlation across the The Organisation for Economic Co-operation and Development (OECD; this is unsurprising as these countries make up much of the target data), as well as across parts of the African continent and Central Asia. Our estimates of Indian net migration broadly follow the WPP trend, but are less erratic; the exodus of workers to the Gulf states, commencing in 2003, is clearly visible. The net migration estimates for Nigeria, meanwhile, are among the most uncertain of our model predictions: in Fig. 5c we show the median relative error (s.d. over mean estimate) for all countries, noting that for Africa, especially sub-Saharan Africa, the uncertainty on the net migration is among the highest in the world. By contrast, uncertainty is relatively lower for European and other rich Western countries, owing to greater availability and higher quality of data as well as more stable migration regimes. The pronounced regional heterogeneity in uncertainty highlights the importance of improving data collection in under-resourced settings as a prerequisite for more precise migration estimates (Supplementary Fig. 6).

## Testing and validation

**Fig. 6:**
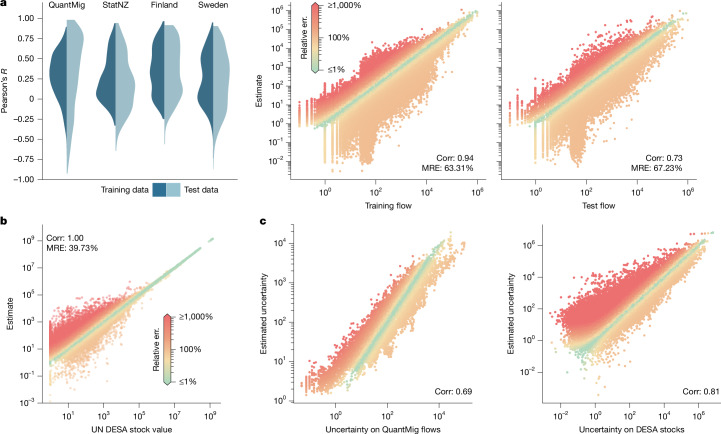
Performance evaluation. **a**, Performance on training and test data. We test the prediction performance using fivefold cross-validation on the target flows. The left-most panel shows the distribution of correlations along flow corridors on the training points (left half of each violin) and test corridors (right half). The distributions for the various flow datasets making up the flow target data over all folds are shown. The two panels to the right show the true and estimated flow values on both training and test sets, with the colour indicating the relative error. We achieve a Pearson *R* correlation of 94% on the training flows, and 73% on the test flows. The median relative error (MRE) is also indicated (Supplementary Fig. 22). **b**, The estimated stock values against the DESA stocks. The Pearson *R* correlation and MRE are also shown. **c**, Comparison of the uncertainties on the estimates (*y*-axes) and the QuantMig flows (left) and DESA stocks (right). The uncertainties on the DESA stocks are themselves estimated via demographic accounting and scaling, as described above (Supplementary Fig. 3). The Pearson *R* correlation is also shown.

We validate our approach by testing how well the neural network can reproduce unseen data (the test data) using fivefold cross-validation: we split the flow corridors into five equally large sets, and train five randomly initialized networks on each set of four folds, using the last fold as the test set. Following a previous work^9^, we chiefly assess performance through correlation metrics rather than mean errors. This allows for meaningful comparisons across datasets with inconsistent migration definitions and accommodates possible constant biases in our estimates. Figure 6a shows that the neural network achieves 94% correlation on the training data, and 73% correlation on the test flows, with only a 4% increase in median relative error (recall that many flows come with considerable uncertainty, and can be small in magnitude, so such a high relative error is not surprising: after all, an estimate of ten for a flow value of five represents a 100% relative error). Although this is the correlation across the entire dataset, we can also examine the distribution of correlations along each corridor (Fig. 6b), finding that the neural network generally matches the correlation distributions of the training data on the test set. In Fig. 6c we compare the estimated uncertainty of our model with that on the QuantMig data for Europe, as well as our estimates of the stock uncertainty with global coverage. The predicted uncertainty on the flows matches the QuantMig values well, while producing generally higher levels of uncertainty on the stocks than obtained through the demographic accounting procedure outlined in the Methods.

We conducted further experiments to assess whether extensive migration data from high-income countries bias the inference of global flows (refer to Supplementary Fig. 23 and the discussion from there onwards). Approximately 20% of the training data consist of flows originating from or directed to Europe or New Zealand (Supplementary Fig. 6). When this subset was withheld, predictions for other regions remained stable, indicating that the model does not transfer dynamics specific to highly developed regions to the rest of the world. To examine whether temporal coverage induces region-specific path dependencies, we withheld all observations after 2015; the predictions in developing regions showed no significant change (Supplementary Fig. 23E).

**Extended Data Fig. 4:**
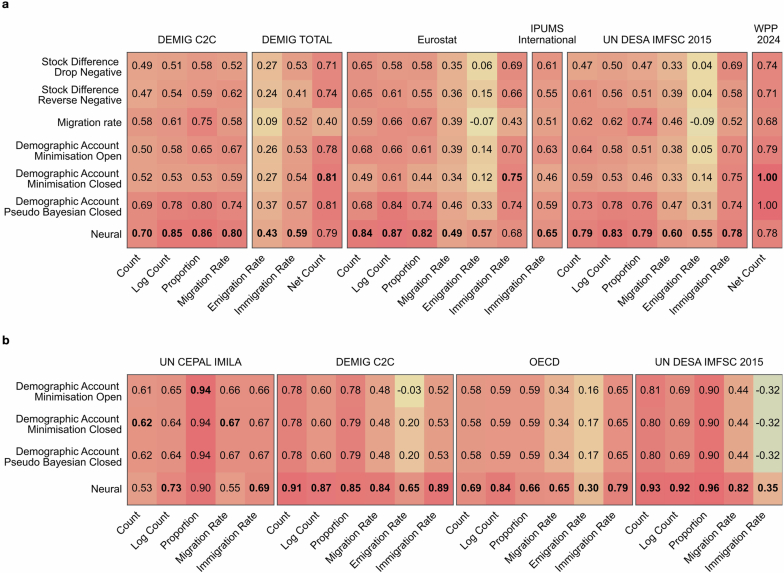
Comparison of bilateral flow estimation methods. All values shown are Pearson correlation coefficients on unseen flow datasets. Since all methods except our own only produce five-year flows, we aggregate the neural estimates to five-year intervals for comparison. The evaluation metrics are as in^9^. See Extended Data Figs. 5–6 for statistical significance values, and Fig. S22 in the SI for cross-fold validation metrics. **a** Correlation on origin-destination flows. **b** Correlation on birth-destination flows.

**Extended Data Fig. 5:**
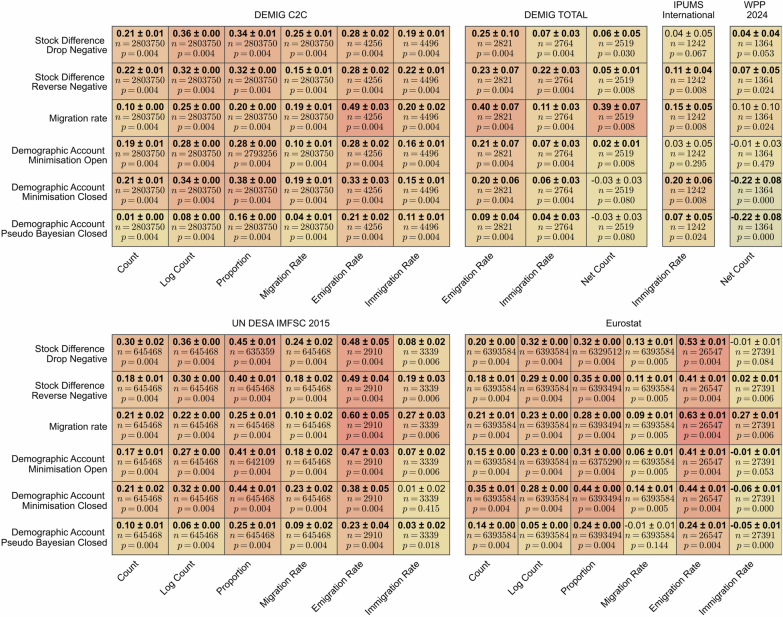
Pairwise comparison of validation metrics for origin-destination flows. For each data collection and validation metric, we report the difference in Pearson correlation coefficient *Δr* between our method and each baseline, with 95% bootstrap confidence intervals (1,000 resamples). *n* denotes the number of origin-destination corridors (bilateral metrics) or country-year observations (total migration metrics) used in each comparison. *p*-values are two-sided bootstrap tests, corrected for multiple comparisons using the Benjamini-Hochberg procedure. A positive *Δr* indicates that the neural network estimates achieve higher correlation with reported data than the competing method. Statistically significant differences are marked in bold.

**Extended Data Fig. 6:**
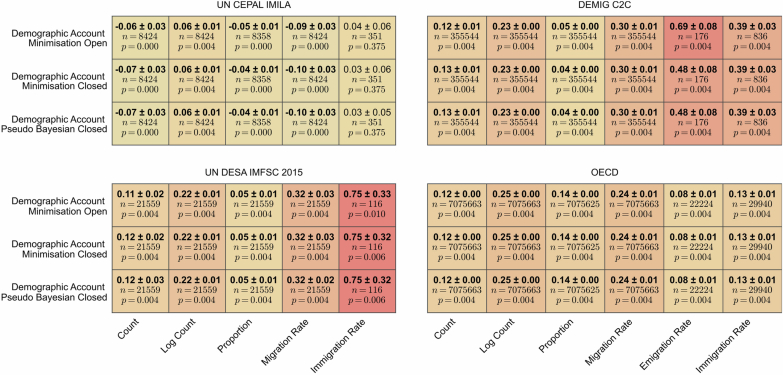
Pairwise comparison of validation metrics for birth-destination flows. Same as Extended Data Fig. 5, but for birth-destination flows (Extended Data Fig. 4b).

We further validate the neural predictions on an additional dataset of unseen bilateral flows and compare their performance with those of the various stock-based approaches outlined in the introduction. The datasets and evaluation metrics are summarized in the Methods and refs. ^9,54^, and broadly comprised bilateral origin- or birth place-destination flows for a small number of (mostly Western) countries. The neural network estimates significantly outperform all other stock-based methods (Extended Data Figs. 4–6); the only exceptions are the UN WPP net migration estimates, where the demographic accounting methods, by design, show a perfect correlation of 1; however, given the methodological issues related to the UN WPP net migration estimates, this is not necessarily desirable.

Finally, we are interested in gauging how sensitive the model is to the various input covariates. To this end, we calculate the elasticity *ν* of each neural network in the ensemble along every covariate dimension, that is, the relative change induced in *T*_*bij*_ ≡ *T* by a relative change in the *k*th covariate:

1 $${\nu }_{k}=| \frac{{\chi }_{k}}{T}\frac{\partial T}{\partial {\chi }_{k}}| =| \frac{\partial \log T}{\partial {\chi }_{k}}| | {\chi }_{k}| .$$

**Extended Data Fig. 7:**
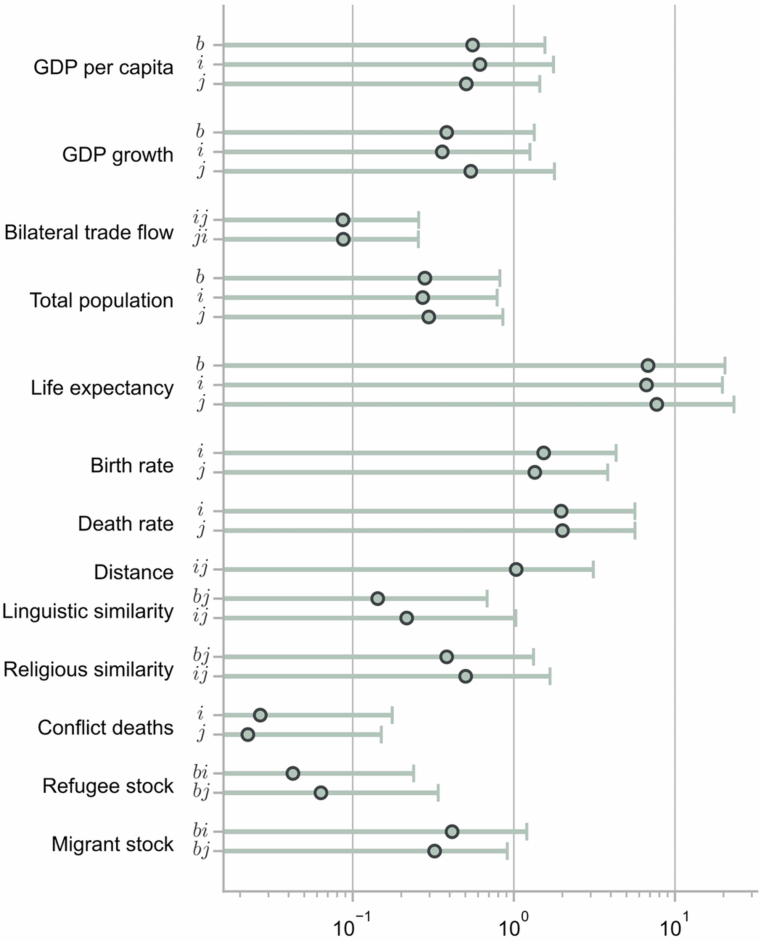
Model elasticities *ν* for each continuous covariate. The marker indicates the mean, with the lines indicating one standard deviation (note the log-scaled x-axis). The elasticity is calculated as an average over each ensemble member and 500,000 randomly sampled year-corridors, giving *n* = 15 × 500,000 = 7.5 m. Higher elasticities indicate that the model is more sensitive to changes in that covariate. See also Extended Data Table 1.

Extended Data Fig. 7 shows that, on average, the model predictions are most sensitive to changes in the life expectancy and mortality rates, presumably as these act as proxies for a country’s (relative) quality of life. Of the economic covariates, gross domestic product (GDP) per capita is the most determinant; religious similarity seems more determinant than linguistic similarity, and data on conflict and refugee stocks are the least determinant.

## Discussion

Data on human movement are notoriously unreliable, noisy and—crucially—absent for large parts of the world. Our work substantially increases both the spatial and temporal resolution of existing migration estimates. We provide a complete set of annual migrant stock values, net migration figures for each country, and bilateral flows disaggregated by country of birth. Reliable migration data are essential to social scientists seeking to establish links between migration and economic or societal outcomes; to epidemiologists, for tracing the dynamics of infectious disease spread; and to demographers, for producing robust population projections. Our dataset—alongside the supplementary training data, including real GDP estimates for 230 countries and territories—offers a valuable new resource. More importantly, this study lays the groundwork for tackling the next frontiers in global migration modelling. One emerging direction is the shift from national-level flows to more geographically granular models. Many of the largest population movements occur in countries rather than across borders^55^. Replacing countries with a finer spatial grid as the unit of analysis would represent a major advance in statistical demography. Our framework is well suited to support this shift. A recent high-resolution demographic dataset at a 10-km^2^ scale was released^56^ and could be used to train such a model. A similar recurrent architecture would be needed to capture spatial correlations, allowing outflows from one cell to influence movements in distant neighbouring areas. As demonstrated, deep neural networks are well equipped to learn such long-range dependencies in both space and time—including via more advanced transformer and graph neural network architectures^57,58^. These models may further enhance our method’s capacity in settings with complex spatial structure.

As we have shown, machine learning allows for the integration of multiple migration data sources and indicators when estimating global migration. Combining data improves the robustness of migration estimates by leveraging the strengths of both traditional and digital-trace-based sources. Covariate information helps to supplement estimates, particularly where migration data are sparse. Consistent with the migration-development nexus literature, we find our estimates to be more sensitive to slower-moving development measures shaping long-term migration opportunities and constraints^59,60^. By contrast, more rapidly varying factors such as refugee numbers and conflict indicators seem less determinant at the global scale, probably because their effects are concentrated in a limited number of corridors rather than exerting broad influence across countries. Deep learning models also capture complex nonlinearities and threshold effects, which can incorporate human system responses to as-yet-unforeseen events, such as climate shocks or emerging conflicts in previously stable regions. Our annual estimates provide a richer empirical basis for global migration than currently available, align with the UN’s move to single-year demographic estimates^61^, and respond to calls for more temporally granular migration data suitable for integration into climate-modelling frameworks^62^ and to support evidence-based policymaking^63^. We note that all estimates presented here are synthetic outputs of a neural network ensemble, and are never derived by direct aggregation of individual-level records. As such, they do not carry identifying information about any individual, including persons with irregular migration status, even for small flow values where aggregate statistics can in principle raise disclosure concerns. Our method learns broad statistical relationships between macroeconomic, demographic and geographic covariates and observed migration patterns; it cannot be used to infer the status, identity, or location of any individual. We nonetheless recognize that improved estimates of migration flows could in principle inform enforcement or border policy, and encourage users of this data to engage with established ethical frameworks for migration data use, including the Inter-Agency Standing Committee (IASC) Operational Guidance on Data Responsibility in Humanitarian Action^64^ and IOM’s Framework for the Ethical Use of Advanced Data Science Methods in the Humanitarian Sector^65^.

## Methods

### Quantifying global migration

Current methods for estimating global migration rely on relatively straightforward techniques compared with the advanced computational approaches adopted in recent years for predicting and explaining human migration and mobility. Estimates of global migrant population stocks, by country of birth and country of residence, are derived from official statistics on foreign-born or foreign populations, with simple interpolation across census years and imputation when data are missing or inconsistent, using regional averages, demographic assumptions or alignment with changes in the population totals^22^.

The availability of migration flow data is much more limited than that on migrant stocks. Countries that publish migration statistics tend to have well-developed statistical infrastructure for monitoring population movements located in rich, developed settings. The scale of migration flows occurring between developing countries and to and from some of the world’s most populous nations is often unknown. Consequently, to estimate origin-destination migration flows at the global level, indirect methods have been developed based on changes in global migrant stock estimates. These methods were reviewed in a previous work^9^, alongside a systematic comparison. They identified six methods, grouping them into three classes.

The first class comprises two stock-differencing approaches, which treat changes in bilateral migrant stocks between census rounds as flows. Negative differences are either set to zero or interpreted as return migration. The second class is a migration-rate approach, which derives transition rates directly from a single stock table by dividing each off-diagonal stock count by the global foreign-born population. These rates are then scaled by an approximation of the total number of global flows, calculated as the sum of absolute net migration flows.

The third class includes three demographic accounting methods, which reconcile changes in birth-place-destination stocks, total population, births and deaths with estimated origin-destination flows. In this framework, adjusted stock tables at the beginning and end of the period are used to define outflow and inflow margins. These margins are then arranged into a three-way array of origin, destination and birthplace flows. Missing values in this array are imputed so that the reconstructed flow table matches the stock-based margin totals. To achieve this, an iterative proportional fitting algorithm, adapted from a past work^73^, is applied to adjust the cell values until the row, column and diagonal constraints are satisfied. Variants of this framework differ in whether inconsistencies in inflow and outflow margins are absorbed into an open demographic system by introducing a residual category^12^ or resolved in a closed demographic system by scaling adjusted stock tables to enforce consistency^13^. A further extension combines two imputation strategies within the closed demographic accounting system by weighting alternative treatments of the diagonal cells in the array that represent non-migrants^14^. The first imputation sets the diagonals to their maximum feasible values, whereas the second applies an independent log-linear fit that relaxes this constraint. The final flow estimates are obtained as a weighted average of the two imputations, with weights calibrated against harmonized European migration flow data. Although each method has trade-offs, the weighted demographic accounting approach produced estimates that were most consistent with reported flow statistics in countries with official data^9^.

All applications of these indirect methods are constrained by the temporal spacing of the available migrant stock data, typically five-year intervals, and by errors or inconsistencies in the underlying stock statistics. As a result, the estimated flows inherit the limitations of the stock data, including inaccuracies in imputations by UN DESA or other agencies, which can affect both the precision and comparability of global migration flow estimates. Moreover, these methods make use of very limited covariate information—only allowing information via a single variable for the seed values of the iterative proportional fitting procedure, which has minimal impact—further restricting their ability to capture corridor-specific dynamics or explanatory factors.

More recently, direct estimates of global migration flows have been produced using large-scale online data sources^21^ (discussed above). The estimates represent a substantial advance over indirect methods, as they are based on observed movements, provide higher temporal resolution, and avoid relying solely on changes in migrant stock data. However, the data cover only a limited number of years, omit several important countries, and will not be updated, restricting their long-term utility.

### Recurrent neural network approach to quantifying global migration

#### Demographic account for global migration estimation

UN DESA provides estimates of global migrant stock *S*_*bi*_(*t*), that is, the number of people born in country *b* living in country *i* at time *t* (ref. ^22^). These data are given at five-year intervals from 1990 to 2020, as well as a recent estimate for 2024. The stocks evolve deterministically according to the equation

2 $${\partial }_{t}{S}_{bi}(t)\,=\,\mathop{\underbrace{{\delta }_{bi}{B}_{i}(t)}}\limits_{{\rm{births}}}\,-\,\mathop{\underbrace{{\gamma }_{i}(t){S}_{bi}(t)}}\limits_{{\rm{deaths}}}\,+\,\mathop{\underbrace{{\sum }_{j}({T}_{bji}\,-\,{T}_{bij})(t)}}\limits_{{\rm{migration}}},$$

where *B*_*i*_ and *γ*_*i*_ are, respectively, the total number of births and the mortality rate of the country of residence, and *δ*_*bi*_ is the Kronecker matrix

3 $${\delta }_{bi}=\left\{\begin{array}{ll}1, & {\rm{if}}\,b=i,\\ 0, & {\rm{e}}{\rm{l}}{\rm{s}}{\rm{e}}.\end{array}\right.$$

The first term in equation (2) simply means that all births in a country increase the native-born stock *S*_*ii*_; the second models population decrease due to deaths; the third models the change in stocks due to migration. *T*_*bij*_ is the flow of migrants born in *b* from country *i* to *j*, and is the quantity we wish to infer. The total flow of people from *i* to *j*, regardless of their place of birth, is of course then given by

4 $${F}_{ij}=\sum _{b}{T}_{bij},$$

while a country’s net migration (arrivals minus departures) is

5 $${{\bf{M}}}_{i}=\sum _{j}{F}_{ji}\,-\,{F}_{ij}.$$

#### Target data

Aside from the stock data, there are numerous datasets of partial observations of the flow *F* and the net migration ${\bf{M}}$ to which we could constrain our estimate $\hat{T}$, although, as mentioned in the introduction, these do not always use consistent definitions of a migration event. The UN DESA World Population Prospects dataset^53^ and the US Census Bureau International Database^70^ both provide estimates of annual net migration for all countries from 1950 to 2024. These figures are mainly calculated as the residual between the total change in population and natural growth (births minus deaths), and for most countries, they are not derived from immigration statistics; we thus do not include them as target variables. Instead, we use net annual migration statistics from around 30 countries and territories in Europe, North America, Oceania and East Asia, sourced from national statistical bureaus (Supplementary Information).

Observations of total origin-destination flows *F* are taken mainly from five sources, which all use a one-year definition of migration flows:

- Harmonized intra-European flows: the QuantMig database^10,11^ provides probabilistic estimates of migration flows between 30 countries in Europe from 2009 to 2019, and is based on publicly available Eurostat data. These have been harmonized to use a common definition of migration, and also provide uncertainty estimates, which we use to weight the target data points in the loss function used to train the neural network (see below).
- National immigration statistics from Sweden, New Zealand and Finland^74–76^: all three countries report total annual in- and out-flows by origin and destination.
- Facebook data^21^: estimates of annual bilateral migration flows between 181 countries from 2019 to 2022 from an analysis of online social media data. We only include annual flows of at least 25 people, as noise was added by the authors to prevent data disclosure, which distorts small values.

Target values are prioritized in this order, meaning that if two datasets both contain values for the same origin–destination pair, we use the source furthest up in the list.

#### Input covariates

Each value *T*_*bij*_ is a flow through a network multi-edge connecting the birth country *b*, the origin *i* and destination *j*. We train a deep neural network to learn a mapping ${{\boldsymbol{\chi }}}_{bij}(t)\mapsto {\hat{T}}_{bij}(t)$, where *χ* is a vector of economic, social and geographic covariates pertaining to the three connected countries (Extended Data Fig. 1a). In the following we give a summary of the covariates used (refer to Extended Data Table 1 for an overview, and the Supplementary Information for further details).

*Demographic covariates*. For each country *b*, *i*, *j*, we include the total population and life expectancies; for the origin and destination countries, we also include birth and death rates, all taken from the UN WPP dataset^53^.

Economic covariates. For each country *b*, *i*, *j*, we include annual real GDP per capita (in constant 2015 US dollars) and annual GDP growth rate (in per cent). Data are taken from the World Bank, UN Conference on Trade and Development, the International Monetary Fund (IMF)^77–80^, as well as national statistical bureaus. Missing values are calculated by deflating nominal to real GDP using the World Bank deflator; where the deflator is not given, we estimate the deflator from neighbouring or similar nations. Gaps are also filled by calculating the GDP growth rate from the Maddison Project dataset^81,82^, which provides GDP in constant 2011 purchasing power parity. The growth rates are then used to extrapolate GDP back or forward in time. We also input bilateral trade flows between origin and destination, given in real 2015 US dollars. These are mostly taken from the harmonized BACI dataset^83,84^, and missing values are again extrapolated using the growth rates from the UN Comtrade and IMF Direction of Trade datasets^85^.

Geographic, cultural, and political covariates. Religious and linguistic proximity—measuring the extent of overlap in religious affiliation and common spoken languages—are taken from the Correlates of War World Religion Data, CIA World Factbook, and USITC Domestic and International Common Language Database datasets^86–88^. Religious similarity measures the overlap in the number of adherents of major religions; for two countries with religious makeup **α**_*i*_ and **α**_*j*_, the similarity score is simply the dot product **α**_*i*_⋅**α**_*j*_. The linguistic similarity score is given by the ‘common spoken language’ index; missing entries are filled with the average of linguistic proximity and common native language^88^. We also include the population-weighted geodesic distance^85^, as well as a number of binary covariates: EU membership of the three indexed nations; binary variables colony_*bj*_ and colony_*ij*_, which are 1 if the first indexed country was ever a colony of the second^89^; as well as the two binary variables *δ*_*bi*_ and *δ*_*bj*_, with *δ* the Kronecker delta (equation (3)), which indicate whether an individual is a native of the origin or the destination.

*Conflict deaths, refugee and migrant stock*. To model short-term, disaster-driven migration, we include data on wars and other shocks that trigger large population movements. We include the total number of deaths related to organized violence in the origin and destination countries, given by the Uppsala Conflict Data programme’s Georeferenced Event Dataset^90,91^. We include the total number of refugees, asylum seekers, and others in need of international protection, as given by the UNHCR’s refugee statistics^72^, and also include the annual change in the figures. Finally, we also input the total migrant stock both in the origin and the destination, *S*_*bi*_(*t*) and *S*_*bj*_(*t*), for each year. As the UN only provides these data points at most every five years, intermediate values are taken from the neural network prediction $\hat{S}$ itself. Where initial values are missing, we extrapolate the stocks back to 1990 using a weighted average of similar countries; the weights are calculated by considering correlations across time and space.

#### Training

We apply the training methodology broadly outlined in a past work^23^. We wish to not only incorporate information on the current state of the world at time *t*, contained in χ_*bij*_(*t*), but also information on the past. To do this, we use a recurrent neural network *u*_*θ*_, which takes as input the covariates (including stocks) as well as a *Z*-dimensional hidden or latent state *z*_*bij*_(*t*). This latent variable represents a ‘memory’ of past changes and their effects on the present flow estimate. The neural network outputs a (log-scaled) estimate of the flow *T*_*bij*_, as well as the updated latent state *z*_*bij*_(*t* + 1), which is then input to the neural network to predict the next point in time:

6 $${u}_{\theta }({{\boldsymbol{\chi }}}_{bij},{{z}}_{bij})=(\log {T}_{bij},{{z}}_{bij}(t+1))\in {{\mathbb{R}}}^{1+{Z}}.$$

Note that the estimates ${\widehat{T}}_{bij}$ and all their derived quantities will be real-valued, despite integer target data. This gives a recursive training procedure, where each output is fed back into the neural network to inform the next estimate (Extended Data Fig. 1a). The latent state *z*_*bij*_ is initialized at zero and can take any value in ${{\mathbb{R}}}^{Z}$.

The neural network consists of a set of trainable parameters ***θ*** that are optimized using the gradient of the loss function, *J*, which is designed to ensure that predicted and observed stocks, net migration values and flows *F*_*ij*_ agree, and is structurally an *L*^2^-loss of all of the different values {*y*_*k*_}. We make two important modifications to this basic loss function: first, we scale the data to make the errors $\widehat{y}-y$ more normal, ensuring the loss function is not dominated by the largest values (this will be addressed below); and second, we weight each term in the loss function by its uncertainty to bias the loss towards values in which we have greater confidence (Extended Data Fig. 1c):

7 $${J}_{\theta }\approx \sum _{k}{w}_{k}{({\widehat{y}}_{k}-{y}_{k})}^{2},$$

with the index *k* ranging over all of the target values in a single batch. The weights *w*_*k*_ are constructed from the relative uncertainty on each point, clamped to the interval [0.5, 2], and normalized such that the mean weight is 1. The QuantMig dataset provides standard errors on the estimates which we use to populate the weights for the flow targets; for all other flow targets, we set the weight to the average weight of the QuantMig weights or 1. For the stocks, we apply the demographic accounting scheme presented in past works^9,12^: given the stock tables for two successive years *S*(*t*_1_), *S*(*t*_2_), we add births and deaths, and constrain the resulting tables to match their midpoint marginals using iterative proportional fitting. Subsequently subtracting births and deaths again gives two demographically balanced stock estimates for each year, from which we can estimate the uncertainty on each value *S*_*bi*_. For the net migration targets, we set the weights to 1 (refer to the Supplementary Information for details).

#### Scaling the input and target data

Much of the input and target data are heavily skewed Poisson distributions, with long tails caused by a small number of strong outliers; to improve learning, it is common practice to transform data to make it more normal. To this end we use a symmetrized Yeo–Johnson transform:

8 $${\psi }_{\lambda }(x)={\rm{s}}{\rm{g}}{\rm{n}}(x)\times \left\{\begin{array}{l}\frac{{(| x| +1)}^{\lambda }\,-\,1}{\lambda }\,{\rm{i}}{\rm{f}}\,\lambda \ne 0,\\ \log (| x| +1)\,{\rm{e}}{\rm{l}}{\rm{s}}{\rm{e}}\end{array}\right.$$

with ${\rm{s}}{\rm{g}}{\rm{n}}(x)$ the sign function. Compared with the standard transformation^92^, the symmetrization ensures that negative values are transformed more evenly. The parameter *λ* can be chosen to move the distribution of *ψ*_*λ*_(*x*) closer towards a normal distribution (Extended Data Fig. 1b); for *λ* = 1, *ψ* is simply the identity. The transformed input data are further normalized to have zero mean and unit variance. Note that the transformation equation (8) is invertible, with inverse ${\psi }_{\lambda }^{-1}$; we can thus always reverse any transformation to obtain the original data. We rescale all non-binary covariates except the religious and linguistic similarity indices to be approximately normal (refer to the Supplementary Information for the *λ* values used for each).

To improve prediction accuracy on edges with smaller flows, we also transform the target data using the above function *ψ*; the loss function then reads

9 $$\begin{array}{c}{J}_{\theta }\,=\,\langle {w}_{bi}^{s}{({\psi }_{{\lambda }_{1}}(\Delta {\hat{S}}_{bi})-{\psi }_{{\lambda }_{1}}(\Delta {S}_{bi}))}^{2}\rangle \\ \,+\,\langle {w}_{i}^{m}{({\psi }_{{\lambda }_{2}}({\hat{{\bf{M}}}}_{i})-{\psi }_{{\lambda }_{2}}({{\bf{M}}}_{i}))}^{2}\rangle \\ \,+\,\langle {w}_{ij}^{f}{({\psi }_{{\lambda }_{3}}({\hat{F}}_{ij})-{\psi }_{{\lambda }_{3}}({F}_{ij}))}^{2}\rangle .\end{array}$$

Here, ⟨⋅⟩ denotes the average over all target values. Observe that we are not matching stock values directly, but rather the difference in stocks over five-year intervals. This is to avoid conditioning the stock value on (possibly erroneous) initial values, and ensure independence of the stock estimates. An optimal initial stock value can be estimated after training using a least squares approach, to fit the time series ${\widehat{S}}_{bi}(t)$ to the data (see below).

#### Model selection and validation

To select the architecture of the neural network, that is, the number of neurons and layers, the activation functions, and the latent dimension *Z*, we use hyperparameter tuning on synthetic data (Supplementary Information). We use a deep network with 7 layers, 60 neurons per layer, and the hyperbolic tangent as the activation function on each layer except the last, where we use the CeLU function^93^

$$\sigma (x)=\max (0,x)+\min (0,\alpha (\exp (x/\alpha )\,-\,1))$$

with *α* = −12. The latent dimension *Z* was set to 100. Also by using a hyperparameter sweep on synthetic data, the scaling parameters for the target data *λ*_*i*_ were all set to 0.7.

### Uncertainty quantification

Uncertainty on the estimates stems from two sources: first, the degree to which the inference problem is ill-posed, meaning the number of possible solutions that fit the data; and second, the uncertainty on the input covariates themselves^23^. The uncertainty arising from the (potential) ill-posedness of the problem can be estimated by training an ensemble of neural networks, thereby generating a distribution on the parameters ***θ*** (ref. ^24^). This is computationally costly, as a family of neural networks need to be trained in parallel. Meanwhile, in theory the uncertainty on the input data can be ‘pushed through’ the trained neural network, as in a previous work^94^. Given a prior distribution *π*_0_ on the input and neglecting the uncertainty on ***θ***, the posterior is simply

10 $$p(T)=({{u}_{\theta }}_{{\rm{\#}}}{\pi }_{0})({\boldsymbol{\chi }}),$$

where *#* indicates a pushforward. For our estimates, we do not know the uncertainty on the inputs except for the initial stock estimate. We train an ensemble of 15 neural networks in parallel to solve the inference problem, and for each draw 100 samples of the initial stocks to estimate overall uncertainty. This gives *n* = 1,500 samples of the flow table $\hat{T}$. In this Article and the accompanying datasets^95^, we provide the average value $\langle \hat{T}\rangle$ and one s.d.

Our uncertainty quantification is designed for the global setting, where estimates cover migrant flows and stocks simultaneously across all countries, often with limited metadata. Past uncertainty quantification work in migration estimation has focused primarily on flows, most notably the Integrated Modelling of European Migration (IMEM) model^96^, which implemented a measurement model with a Bayesian hierarchical framework to explicitly reconcile definitional mismatches, timing criteria, and measurement error for different European countries’ flow data, drawing on expert opinions^97^. IMEM was a direct predecessor to the QuantMig project^11^ and has since been extended to incorporate further data sources^98–100^. Further adaptations have also been made to the IMEM model to estimate European bilateral stocks with uncertainty, independent of flow data^101^, again relying on richer metadata on input migration measures than are available globally. By contrast, our approach explicitly links changes in migrant stock data to their estimated flows. More direct measures of measurement uncertainty for the flow data are not explicitly included as we include only relatively high-quality flow measures with a similar definition, unlike previous models of European migration flows, where data quality varied considerably between countries. Note that, as we use the QuantMig estimates and their uncertainties as target data, these explicit measures are fed into our model.

### Calculating the elasticity

The elasticity equation (1) is further given by

$${\nu }_{k}=\left|\frac{\partial {u}_{\theta }}{\partial {x}_{k}}\right||{x}_{k}|,$$

with *x*_*k*_ the untransformed *k*th covariate. As the neural network takes *ψ*_*λ*_-transformed covariates as input, we can apply the chain rule to *χ*_*k*_ = *ψ*_*λ*_(*x*_*k*_) to obtain

$${\nu }_{k}=|\frac{\partial {u}_{\theta }}{\partial {\chi }_{k}}\frac{\partial {\psi }_{\lambda }}{\partial {x}_{k}}||{x}_{k}|.$$

### Calibrating the initial stock value

To generate a time series of migrant stocks ${\widehat{S}}_{bi}(t)$, an initial condition *S*_*bi*_(*t*_0_ = 1990) is required to solve the stock evolution equation (2) forward in time. This initial value may be taken from existing sources such as UN DESA where available; however, rather than conditioning on a potentially noisy or inconsistent baseline, we instead calculate a constant offset that best aligns modelled and observed values while accounting for demographic dynamics. Let *S*_*bi*_(*t*) be the observed migrant stock, ${\widehat{S}}_{bi}(t)$ the corresponding model prediction generated from an arbitrary initial value *S*_*bi*_(*t*_0_), ${w}_{bi}^{s}(t)$ the weight on each observation, and *γ*_*i*_(*t*) the mortality rate of country *i*. Define the survival fraction ${\widetilde{\gamma }}_{i}(t)$ as the proportion of individuals alive in 1990 who are still alive in year *t*:

$${\widetilde{\gamma }}_{i}(t)=\prod _{{t}_{0} < \tau \le t}(1\,-\,{\gamma }_{i}(\tau ))\ge 0$$

and $\widetilde{\gamma }({t}_{0})=1$. The optimal offset ${\rho }_{bi}\in {\mathbb{R}}$ of the initial stock value ${\widehat{S}}_{bi}({t}_{0})$ is then computed by minimizing the weighted squared error between observed and predicted stocks:

11 $${\rho }_{bi}=\frac{{\sum }_{t}{\widetilde{\gamma }}_{i}{w}_{bi}^{s}({S}_{bi}(t)\,-\,{\widehat{S}}_{bi}(t))}{{\sum }_{t}{w}_{bi}{\widetilde{\gamma }}_{i}^{2}}\in {\mathbb{R}},$$

and the baseline-shifted stock then given by ${\widehat{S}}_{bi}(t)+{\widetilde{\gamma }}_{i}(t){\rho }_{bi}$. *ρ*_*bi*_ is further constrained to ensure that all resulting stocks remain non-negative.

### Comparison and validation

We further validate our approach on a dataset of unseen bilateral origin- and birth-destination flows. As the datasets do not use a temporally consistent definition of migration (or include many different measures of migration), we do not calculate prediction errors directly, but rather use a series of correlation metrics similar to those presented in a previous work^9^. Unlike in Fig. 6a, we calculate correlations between the entire dataset *Y*:

- Count: Pearson correlation coefficient on *Y*.
- Log count: Pearson correlation coefficient on $\log (Y+1)$.
- Proportion: Pearson correlation on *y*_*ij*_/∑_*i*_*y*_*ij*_ if the observation *y*_*ij*_ was reported by the destination country, else *y*_*ij*_/∑_*j*_*y*_*ij*_.
- Migration rate: Pearson correlation on *y*_*ij*_/*P*_*i*_, with *P*_*i*_ the total population of the origin.
- Emigration rate: Pearson correlation on ∑_*j*_*y*_*ij*_/*P*_*i*_.
- Immigration rate: Pearson correlation on ∑_*i*_*y*_*ij*_/*P*_*j*_.
- Net count: Pearson correlation on the net count ∑_*i*_(*y*_*ij*_ − *y*_*ji*_).

Note that in Extended Data Fig. 4 we show correlations on both the total origin-destination flow (∑_*b*_*T*_*bij*_) as well as the total birth-destination flow (∑_*i*_*T*_*bij*_). We use the following validation datasets:

- DEMIG C2C^7^: bilateral flow data for 34 reporting (mostly European) countries from 1990–2011; this dataset contains both origin- and birth-destination flows.
- DEMIG TOTAL^102^: total immigration and emigration flows, as well as net counts, from 1990–2011.
- Eurostat^67^: bilateral origin-destination flows, mostly within Europe, from 1998–2019.
- IPUMS International^103^: immigration totals from census data covering the period 1990–2016.
- UN DESA IMFSC^8^: bilateral origin-destination and birth-destination flows, reported by 45 (mainly European) countries from 1990–2013.
- UN CEPAL IMILA^69^: bilateral birth-destination flow data to and from Latin American countries from 1990–2013. Excludes return migration of native-born emigrants.
- OECD^68^: birth-destination flows for OECD countries, 1995–2013.
- WPP^53^: UN WPP net migration estimates for all countries, 2024 revision, 1990–2020.

Extended Data Fig. 4 shows the metrics for the various stock differencing methods outlined in the introduction, as well as our neural estimates. As all methods except our own produce five-year flows, we aggregate our results up to the five-year level. The closed demographic accounting methods have been adjusted to match the same demographic residuals used to calculate the UN WPP net migration figures, hence their correlation with that dataset is 1. Extended Data Figs. 5 and 6 show the statistical significance of the correlation score differences between our method and each of the ones described above.

### Reporting summary

Further information on research design is available in the Nature Portfolio Reporting Summary linked to this article.

## Online content

Any methods, additional references, Nature Portfolio reporting summaries, source data, extended data, supplementary information, acknowledgements, peer review information; details of author contributions and competing interests; and statements of data and code availability are available at 10.1038/s41586-026-10611-7.

## Supplementary information

Supplementary Information Supplementary Information, including Supplementary Figs. 1–23, Supplementary References, and the following four sections: Target data; Input data; Validation on synthetic data; and Analysing the bias.

Reporting Summary

Peer Review File

## Figures and Tables

**Extended Data Table 1:**
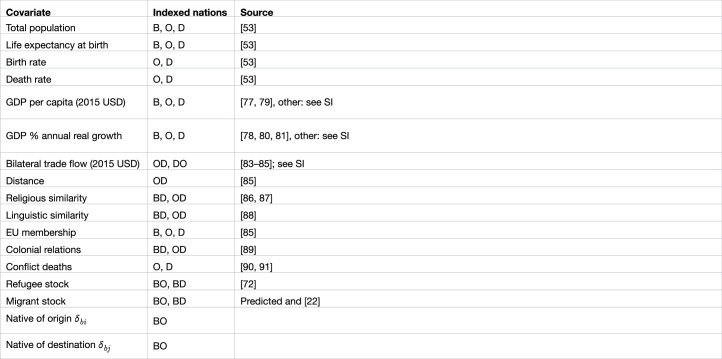
Input covariates *χ*_*bij*_ used to train the model

The ‘indexed nations’ column indicates for which indices data are included, i.e. place of birth (B), origin (O), or destination (D). Bilateral variables are naturally indexed by two places.

## Data Availability

The trained ensemble, training data, and all estimates are available on HuggingFace via https://huggingface.co/datasets/ThGaskin/Migration_flows and 10.57967/hf/8902 (ref. ^95^).

## Code availability

All code used to train the networks, generate samples, and evaluate the trained models is available on GitHub via https://github.com/ThGaskin/Migration_flows and 10.5281/zenodo.19555786 (ref. ^104^). Detailed instructions on installing and running the code are provided in the repository.

## References

[1] Castles, S. Migration, crisis, and the global labour market. *Globalizations* **8**, 311–324 (2011).

[2] Ratha, D., Mohapatra, S. & Scheja, E. *Impact of Migration on Economic and Social Development: A Review of Evidence and Emerging Issues* Policy Research Working Paper 5558 (World Bank, 2011).

[3] Dempster, H. & Hargrave, K.* Understanding Public Attitudes Towards Refugees and Migrants* Working Paper 512 (Overseas Development Institute, 2017).

[4] Skeldon, R. *Migration and Development**Report* UN/POP/EGM-MIG/2008/4 (United Nations Economic and Social Commission for Asia and the Pacific, 2008).

[5] Abel, G. J., Brottrager, M., Crespo Cuaresma, J. & Muttarak, R. Climate, conflict and forced migration. *Glob. Environ. Change* **54**, 239–249 (2019).

[6] Willekens, F., Massey, D., Raymer, J. & Beauchemin, C. International migration under the microscope. *Science* **352**, 897–899 (2016). 27199405 10.1126/science.aaf6545 PMC5508757

[7] DEMIG Project. *DEMIG C2C* v.1.2 (International Migration Institute, 2015).

[8] United Nations Department of Economic and Social Affairs, Population Division. *International Migration Flows to and from Selected Countries: The 2015 Revision* (United Nations, 2015).

[9] Abel, G. J. & Cohen, J. E. Bilateral international migration flow estimates for 200 countries. *Sci. Data* **6**, 82 (2019). 31209218 10.1038/s41597-019-0089-3 PMC6572777

[10] Smith, P. W. F. & Aristotelous, G. *MCMC Simulations from the Posterior Distributions of European Migration Flows* (Zenodo, 2023); 10.5281/zenodo.8224827.

[11] Smith, P. W., Keilman, N., Aristotelous, G. & Bijak, J. in *From Uncertainty to Policy: A Guide to Migration* Scenarios (ed. Bijak. J.) 53–64 (Edward Elgar, 2024).

[12] Abel, G. Estimating global migration flow tables using place of birth data. *Demographic Res.* **28**, 505–546 (2013).

[13] Abel, G. J. & Sander, N. Quantifying global international migration flows. *Science* **343**, 1520–1522 (2014). 24675962 10.1126/science.1248676

[14] Azose, J. J. & Raftery, A. E. Estimation of emigration, return migration, and transit migration between all pairs of countries. *Proc. Natl Acad. Sci. USA* **116**, 116–122 (2018). 30584106 10.1073/pnas.1722334116 PMC6320531

[15] Carrasco, J. I., Akbaritabar, A., Godin, M. & Vargas-Silva, C. Prioritizing global equity in migration research. *Humanities Soc. Sci. Commun.* **13**, 16 (2025).

[16] Willekens, F. Monitoring international migration flows in Europe. *Eur. J. Pop. Rev. Europ. Dém.* **10**, 1–42 (1994). 10.1007/BF01268210 12318848

[17] Willekens, F. Evidence-based monitoring of international migration flows in Europe. *J. Official Stat.* **35**, 231–277 (2019).

[18] United Nations Department of Economic and Social Affairs, Population Division. *Recommendations on Statistics of International Migration, Revision 1* (United Nations, 1998).

[19] International Labour Organization. *Statistics on Migrant Workers* (International Labour Organization, 2025); https://ilostat.ilo.org/topics/labour-migration/.

[20] International Organization for Migration. *Labour Migration* (International Organization for Migration, 2024); https://www.migrationdataportal.org/themes/labour-migration-old.

[21] Chi, G., Abel, G. J., Johnston, D., Giraudy, E. & Bailey, M. Measuring global migration flows using online data. *Proc. Natl Acad. Sci. USA* **122**, e2409418122 (2025). 40299700 10.1073/pnas.2409418122 PMC12067262

[22] United Nations Department of Economic and Social Affairs, Population Division. *International Migrant Stock* (United Nations, 2025); https://www.un.org/development/desa/pd/content/international-migrant-stock.

[23] Gaskin, T., Pavliotis, G. A. & Girolami, M. Neural parameter calibration for large-scale multi-agent models. *Proc. Natl Acad. Sci. USA* **120**, e2216415120 (2023). 10.1073/pnas.2216415120 PMC9963791 36763529

[24] Gaskin, T., Pavliotis, G. A. & Girolami, M. Inferring networks from time series: a neural approach. *PNAS Nexus* **3**, pgae063 (2024). 38560526 10.1093/pnasnexus/pgae063 PMC10978060

[25] Beine, M., Docquier, F. & Özden, Ç Diasporas. *J. Dev. Econ.* **95**, 30–41 (2011).

[26] Bertoli, S. & Fernández-Huertas Moraga, J. The size of the cliff at the border. *Reg. Sci. Urban Econ.* **51**, 1–6 (2015).

[27] Beine, M. & Parsons, C. Climatic factors as determinants of international migration. *Scandinavian J. Econ.* **117**, 723–767 (2015).

[28] Poot, J., Alimi, O., Cameron, M. P. & Maré, D. C.* The Gravity Model of Migration: The Successful Comeback of an Ageing Superstar in Regional Science* (The Institute for the Study of Labor, 2016).

[29] Beyer, R. M., Schewe, J. & Lotze-Campen, H. Gravity models do not explain, and cannot predict, international migration dynamics. *Human. Social Sci. Commun.* **9**, 56 (2022).

[30] Cho, K. et al. Learning phrase representations using RNN encoder–decoder for statistical machine translation. In *Proc. 2014 Conference on Empirical Methods in Natural Language Processing (EMNLP)* (eds. Moschitti, A., Pang, B. & Daelemans, W.) 1724–1734 (Association for Computational Linguistics, 2014).

[31] Sutskever, I., Vinyals, O. & Le, Q. V. Sequence to sequence learning with neural networks. In *Proc. 28th International Conference on Neural Information Processing Systems* Vol. 2, 3104–3112 (MIT Press, 2014).

[32] Bijak, J. *Forecasting International Migration in Europe: A Bayesian View* (Springer, 2011).

[33] Pappalardo, L., Manley, E., Sekara, V. & Alessandretti, L. Future directions in human mobility science. *Nat. Comput. Sci.* **3**, 588–600 (2023). 38177737 10.1038/s43588-023-00469-4

[34] Luca, M., Barlacchi, G., Lepri, B. & Pappalardo, L. A survey on deep learning for human mobility. *ACM Comput. Surveys* **55**, 1–44 (2023).

[35] Rong, C., Ding, J. & Li, Y. An interdisciplinary survey on origin-destination flows modeling: Theory and techniques. *ACM Comput. Surveys* **57**, 1–49 (2025).

[36] Simini, F., Barlacchi, G., Luca, M. & Pappalardo, L. A deep gravity model for mobility flows generation. *Nat. Commun.* **12**, 6576 (2021). 34772925 10.1038/s41467-021-26752-4 PMC8589995

[37] Terroso-Sáenz, F. & Muñoz, A. Nation-wide human mobility prediction based on graph neural networks. *Appl. Intell.* **52**, 4144–4160 (2022). 10.1007/s10489-021-02645-3 PMC8288072 34764610

[38] Rong, C., Feng, J. & Ding, J. Goddag: generating origin-destination flow for new cities via domain adversarial training. *IEEE Trans. Knowledge Data Eng.* **35**, 10048–10057 (2023).

[39] Xu, Y. et al. Predicting human mobility flows in cities using deep learning on satellite imagery. *Nat. Commun.* **16**, 10372 (2025). 41285790 10.1038/s41467-025-65373-z PMC12645052

[40] Cabanas-Tirapu, O., Danús, L., Moro, E., Sales-Pardo, M. & Guimerà, R. Human mobility is well described by closed-form gravity-like models learned automatically from data. *Nat. Commun.* **16**, 1336 (2025). 39904994 10.1038/s41467-025-56495-5 PMC11794706

[41] Robinson, C. & Dilkina, B. A machine learning approach to modeling human migration. In *Proc. 1st ACM SIGCAS Conference on Computing and Sustainable Societies*, *‘COMPASS ’18’*10.1145/3209811.3209868 (Association for Computing Machinery, 2018).

[42] Weber, H. How well can the migration component of regional population change be predicted? A machine learning approach applied to German municipalities. *Comparative Pop. Stud.* **45**, 143–178 (2020).

[43] Gu, X., Tang, X., Chen, T. & Liu, X. Predicting the network shift of large urban agglomerations in China using the deep-learning gravity model: a perspective of population migration. *Cities* **145**, 104680 (2024).

[44] Hauer, M. E. Migration induced by sea-level rise could reshape the us population landscape. *Nat. Clim. Change* **7**, 321–325 (2017).

[45] Robinson, C., Dilkina, B. & Moreno-Cruz, J. Modeling migration patterns in the USA under sea level rise. *PloS ONE* **15**, e0227436 (2020). 31968017 10.1371/journal.pone.0227436 PMC6975524

[46] Schutte, S., Vestby, J., Carling, J. & Buhaug, H. Climatic conditions are weak predictors of asylum migration. *Nat. Commun.* **12**, 2067 (2021). 33824306 10.1038/s41467-021-22255-4 PMC8024373

[47] Molina, M. D., Chau, N., Rodewald, A. D. & Garip, F. How to model the weather-migration link: a machine-learning approach to variable selection in the Mexico–U.S. context. *J. Ethnic Migration Stud.* **49**, 465–491 (2023).

[48] Carammia, M., Iacus, S. M. & Wilkin, T. Forecasting asylum-related migration flows with machine learning and data at scale. *Sci. Rep.* **12**, 1457 (2022). 35087096 10.1038/s41598-022-05241-8 PMC8795256

[49] Boss, K., Groeger, A., Heidland, T., Krueger, F. & Zheng, C. Forecasting bilateral asylum seeker flows with high-dimensional data and machine learning techniques. *J. Econ. Geography* **25**, 3–19 (2025).

[50] Council on Foreign Relations. *Nigeria Security Tracker* (Council on Foreign Relations, 2023); https://www.cfr.org/nigeria/nigeria-security-tracker/p29483.

[51] Mark, M. Thousands flee as Boko Haram seizes military base on Nigeria border. *The Guardian* (6 January 2015); https://www.theguardian.com/world/2015/jan/05/boko-haram-key-military-base-nigeria-chad-border.

[52] International Organization for Migration. *IOM Highlights Humanitarian Needs for 2.4 Million Displaced in Northeast Nigeria* (International Organization for Migration, 2024); https://www.iom.int/news/iom-highlights-humanitarian-needs-24-million-displaced-northeast-nigeria.

[53] United Nations Department of Economic and Social Affairs, Population Division. *World Population Prospects 2024* (United Nations Department of Economic and Social Affairs, Population Division, 2024).

[54] Abel, G. J. & Cohen, J. E. Bilateral international migration flow estimates updated and refined by sex. *Sci. Data* **9**, 173 (2022). 35422105 10.1038/s41597-022-01271-z PMC9010437

[55] Rees, P. et al. The impact of internal migration on population redistribution: an international comparison. *Pop. Space Place* **23**, e2036 (2017).

[56] Niva, V. et al. World’s human migration patterns in 2000–2019 unveiled by high-resolution data. *Nat. Human Behav.* **7**, 2023–2037 (2023). 37679443 10.1038/s41562-023-01689-4 PMC10663150

[57] Bronstein, M. M., Bruna, J., LeCun, Y., Szlam, A. & Vandergheynst, P. Geometric deep learning: going beyond Euclidean data. *IEEE Signal Processing Magazine* **34**, 18–42 (2017).

[58] Wu, L., Cui, P., Pei, J. & Zhao, L. *Graph Neural Networks: Foundations, Frontiers, and Applications* (Springer, 2022).

[59] Skeldon, R. *Migration and Development: A Global Perspective* (Longman, 1997).

[60] de Haas, H. Migration and development: a theoretical perspective. *Intl Migration Review* **44**, 227–264 (2010). 10.1111/j.1747-7379.2009.00804.x PMC4744987 26900199

[61] United Nations Department of Economic and Social Affairs, Population Division. *World Population Prospects 2022* (United Nations Department of Economic and Social Affairs, Population Division, 2022).

[62] Beyer, R. M., Schewe, J. & Abel, G. J. Modeling climate migration: dead ends and new avenues. *Front. Clim.* **5**, 1212649 (2023).

[63] United Nations General Assembly. *Global Compact for Safe, Orderly and Regular Migration* (United Nations General Assembly, 2018).

[64] Inter-Agency Standing Committee. *Operational Guidance on Data Responsibility in Humanitarian Action* (Inter-Agency Standing Committee, 2023).

[65] Dodgson, K., Hirani, P., Trigwell, R. & Bueermann, G. *A Framework for the Ethical Use of Advanced Data Science Methods in the Humanitarian Sector* Technical Report (International Organization for Migration, 2020).

[66] International Labour Organization. *International Labour Migration Statistics Database (ASEAN ILMS)* (International Labour Organization, 2023); https://www.ilo.org/resource/other/international-labour-migration-statistics-database-asean-ilms.

[67] Eurostat. *Immigration by Age Group, Sex and Country of Previous Residence* (Eurostat, 2022).

[68] Organisation for Economic Co-operation and Development. *International Migration Database* (Organisation for Economic Co-operation and Development, 2025); https://www.oecd.org/en/data/datasets/oecd-databases-on-migration.html.

[69] The Latin American and Caribbean Demographic Centre (CELADE) of the Economic Commission for Latin America and the Caribbean (ECLAC) of the United Nations. *I**nvestigación de la Migración Internacional en Latinoamérica (IMILA)* (The Latin American and Caribbean Demographic Centre (CELADE) of the Economic Commission for Latin America and the Caribbean (ECLAC) of the United Nations, 2020); https://celade.cepal.org/bdcelade/imila/.

[70] United States Census Bureau. *International Database: World Population Estimates and Projections* (United States Census Bureau, 2024); https://www.census.gov/programs-surveys/international-programs/about/idb.html.

[71] Institut national de la statistique et des études économiques. *Composantes de la Croissance Démographique* (Institut national de la statistique et des études économiques, 2025); https://www.insee.fr/fr/statistiques/2381468.

[72] United Nations High Commissioner for Refugees. *UNHCR Refugee Statistics Database* (United Nations High Commissioner for Refugees, 2024); https://www.unhcr.org/refugee-statistics/download/?v2url=2dfce8.

[73] Deming, W. E. & Stephan, F. F. On a least squares adjustment of a sampled frequency table when the expected marginal totals are known. *Ann. Math. Stat.* **11**, 427–444 (1940).

[74] Statistics Sweden. *Immigrations and Emigrations by Country of Emi-/Immigration, Region of Birth, Age and Sex: Year 2000–2024* (Statistics Sweden 2025); https://www.statistikdatabasen.scb.se/pxweb/en/ssd/START__BE__BE0101__BE0101J/ImmiEmiFlyttN.

[75] Statistics New Zealand. *Permanent and Long-term Migration By Every Country of Residence and Citizenship* (Statistics New Zealand, 2025); https://infoshare.stats.govt.nz/Default.aspx.

[76] Statistics Finland. *Immigration and Emigration by Country of Departure or Arrival, Origin and Region, 1990-2023 (11ab)* (Statistics Finland, 2024); https://pxdata.stat.fi/PxWeb/pxweb/en/StatFin/StatFin__muutl/statfin_muutl_pxt_11ab.px/.

[77] The World Bank. *World Bank Open Data: GDP Per Capita (Constant 2015 US$)* (The World Bank, 2023); https://data.worldbank.org/indicator/NY.GDP.PCAP.KD.

[78] The World Bank. *World Bank Open Data: GDP growth (Annual %)* (The World Bank, 2023); https://data.worldbank.org/indicator/NY.GDP.MKTP.KD.ZG.

[79] United Nations Conference on Trade and Development. *Gross Domestic Product: Total and Per Capita, Current and Constant (2015) Prices, Annual* (United Nations Conference on Trade and Development, 2023); https://unctadstat.unctad.org/datacentre/dataviewer/US.GDPTotal.

[80] International Monetary Fund. *World Economic Outlook Database, October 2024* (International Monetary Fund, 2024); https://www.imf.org/en/Publications/WEO/weo-database/2024/October.

[81] Bolt, J. & van Zanden, J. L. Maddison-style estimates of the evolution of the world economy: a new 2023 update. *J. Econ. Surveys* **39**, 631–671 (2025).

[82] Bolt, J. & van Zanden, J. L. Maddison-style estimates of the evolution of the world economy: a new 2023 update. *J. Econ. Surveys* **39**, 631–671 (2024).

[83] Gaulier, G. & Zignago, S. *BACI: International Trade Database at the Product-Level. The 1994-2007 Version* Working Papers 2010-23 (CEPII, 2010).

[84] Centre d’Études Prospectives et d’Informations Internationales. *BACI Dataset* (Centre d’Études Prospectives et d’Informations Internationales, 2025); https://www.cepii.fr/CEPII/en/bdd_modele/bdd_modele_item.asp?id=37.

[85] Conte, M., Cotterlaz, P. & Mayer, T. *The CEPII Gravity Database* Working Papers 2022-05 (CEPII Research Center, 2022).

[86] Maoz, Z. & Henderson, E. A. The World Religion Dataset, 1945–2010: logic, estimates, and trends. *Intl Interac.* **39**, 265–291 (2013).

[87] Central Intelligence Agency. *The World Factbook 2024* (Central Intelligence Agency, 2024).

[88] Gurevich, T., Herman, P. R., Toubal, F. & Yotov, Y. V. *The Domestic and International Common Language Database* USITC Economics Working Paper 2024–03–A (US International Trade Commission, 2024).

[89] Gurevich, T. & Herman, P. *The Dynamic Gravity Dataset: 1948–2016* Economics Working Paper 2018-02-A (US International Trade Commission, 2018).

[90] Davies, S., Engström, G., Pettersson, T. & Öberg, M. Organized violence 1989–2023, and the prevalence of organized crime groups. *J. Peace Res.* **61**, 673–693 (2024).

[91] Sundberg, R. & Melander, E. Introducing the UCDP georeferenced event dataset. *J. Peace Res.* **50**, 523–532 (2013).

[92] Yeo, I. & Johnson, R. A. A new family of power transformations to improve normality or symmetry. *Biometrika* **87**, 954–959 (2000).

[93] Barron, J. T. Continuously differentiable exponential linear units. Preprint at https://arxiv.org/abs/1704.07483 (2017).

[94] Gaskin, T., Demirel, G., Wolfram, M.-T. & Duncan, A. Modelling global trade with optimal transport. *Nat. Commun.* **17**, 2947 (2026). 41714620 10.1038/s41467-026-69694-5 PMC13031949

[95] Gaskin, T. & Abel, G. Migration flows datasets. *Hugging Face*10.57967/hf/8092 (2026).

[96] Raymer, J., Wiśniowski, A., Forster, J. J., Smith, P. W. & Bijak, J. Integrated Modeling of European Migration. *J. Amer. Stat. Assoc.* **108**, 801–819 (2013).

[97] Wiśniowski, A. et al. Utilising expert opinion to improve the measurement of international migration in Europe. *J. Official Stat.* **29**, 583–607 (2013).

[98] Del Fava, E., Wiśniowski, A. & Zagheni, E. *Modelling International Migration Flows by Integrating Multiple Data Sources* (Center for Open Science, 2019); https://ideas.repec.org/p/osf/socarx/cma5h.html.

[99] Dańko, M. J., Wiśniowski, A., Jasilionis, D., Jdanov, D. A. & Zagheni, E. Assessing the quality of data on international migration flows in Europe: the case of undercounting. *Migration Stud.* **12**, mnae014 (2024).

[100] Dańko, M. J. *The Human Migration Database (HMigD)* (MPIDR, 2025).

[101] Yildiz, D. et al. Integrating traditional and social media data to predict bilateral migrant stocks in the European Union. *Int. Migration Rev.* **59**, 90–118 (2024).

[102] DEMIG. *DEMIG TOTAL* v.1.5 (International Migration Institute, 2015).

[103] Minnesota Population Center. *Integrated Public Use Microdata Series* v.7.3 (Minnesota Population Center, 2020).

[104] Gaskin, T. & Abel, G. Migration Flows Code. *Zenodo*10.5281/zenodo.19555786 (2026).

