# Supplementary material for: Deep learning four decades of human migration
Source: Nature. 2026 Jun 10;655(8121):148–57. doi: 10.1038/s41586-026-10611-7 (PMC13322962; doi:10.1038/s41586-026-10611-7)
Supplement: Supplementary file 1 — Supplementary Information, including Supplementary Figs. 1–23, Supplementary References, and the following four sections: Target data; Input data; Validation on synthetic data; and Analysing the bias. [file 41586_2026_10611_MOESM1_ESM.pdf]

nature portfolio

https://doi.org/10.1038/s41586-026-10611-7

Supplementary information

Deep learning four decades of human migration

In the format provided by the
authors and unedited

Deep learning four decades of human migration
Supporting Information

Thomas Gaskin & Guy J. Abel

Contents

Target data **2**
Net migration data 2
Migrant stocks 5
Flow data 8

Input data **10**
GDP per capita and GDP annual growth 10
Bilateral trade 13
Total population, Life Expectancy, Birth and Death rates 13
Distance 13
Linguistic similarity 14
Religious similarity 14
EU Membership 14
Colonial ties 14

Validation on synthetic data **14**

Analysing the bias **19**

In the following we detail collection and assembly of the input and target data used to train the neural network, as well the validation process used to select the neural network architecture.

Target data

Net migration data

To train the neural network, we use target net migration statistics for a small number of countries. This data are mostly taken from National Statistics Bureaus, as well as the UN 2024 World Population Prospects for a handful of additional countries and periods. In figures S1 we show national figures alongside the UN WPP 2024 and WPP 2022 Revision data for some selected countries. UN WPP 2024 figures generally agree well with national estimates for periods pre-2010; larger discrepancies typically occur around 2020 due to the pandemic, the war in Ukraine, and a probable lack or lag in new demographic data reaching demographers at UN DESA.

- Australia: data from 2004 onwards provided by the Australian Bureau of Statistics [1]. Pre-2004 we use UN WPP 2024 data.
- Austria: data from 2002 onwards provided by Statistik Austria [2]. Pre-2002 we use UN WPP 2024 data.
- Belgium: data from 2000 onwards provided by StatBEL [3]. Pre-2000 we use UN WPP 2024 data.
- Bulgaria: data from 2010 onwards provided by the National Statistical Office of Bulgaria [4].
- Canada: data provided by Statistics Canada [5]. Since figures are given for years ending in the summer, we use the midpoint average as an approximation for calendar-year net migration figures.
- Czech Republic: data provided by the Czech Statistical Office [6].
- Denmark: data provided by Statistics Denmark [7].
- Estonia: data from 2000 onwards provided by Statistics Estonia [8]; since the figures agree with the UN WPP 2024 data, pre-2000 we use UN WPP 2024 data.
- Finland: data provided by Statistics Finland [9].
- France: data are provided by INSEE (Institut National de la statistique et des études économiques) [10].
- Germany: data are provided by the Statistisches Bundesamt [11].
- Iceland: data provided by Statistics Iceland [12].
- Ireland: data provided by the Central Statistics Office [13]; these are counted from April to April and thus need to be shifted back and interpolated accordingly.
- Italy: data from Istat [14, 15].
- Japan: data are provided by the Japan Immigration Services Agency [16].
- Latvia: data are provided by the Statistics Agency of Latvia [17].
- Lithuania: data are provided by Statistics Lithuania [18].

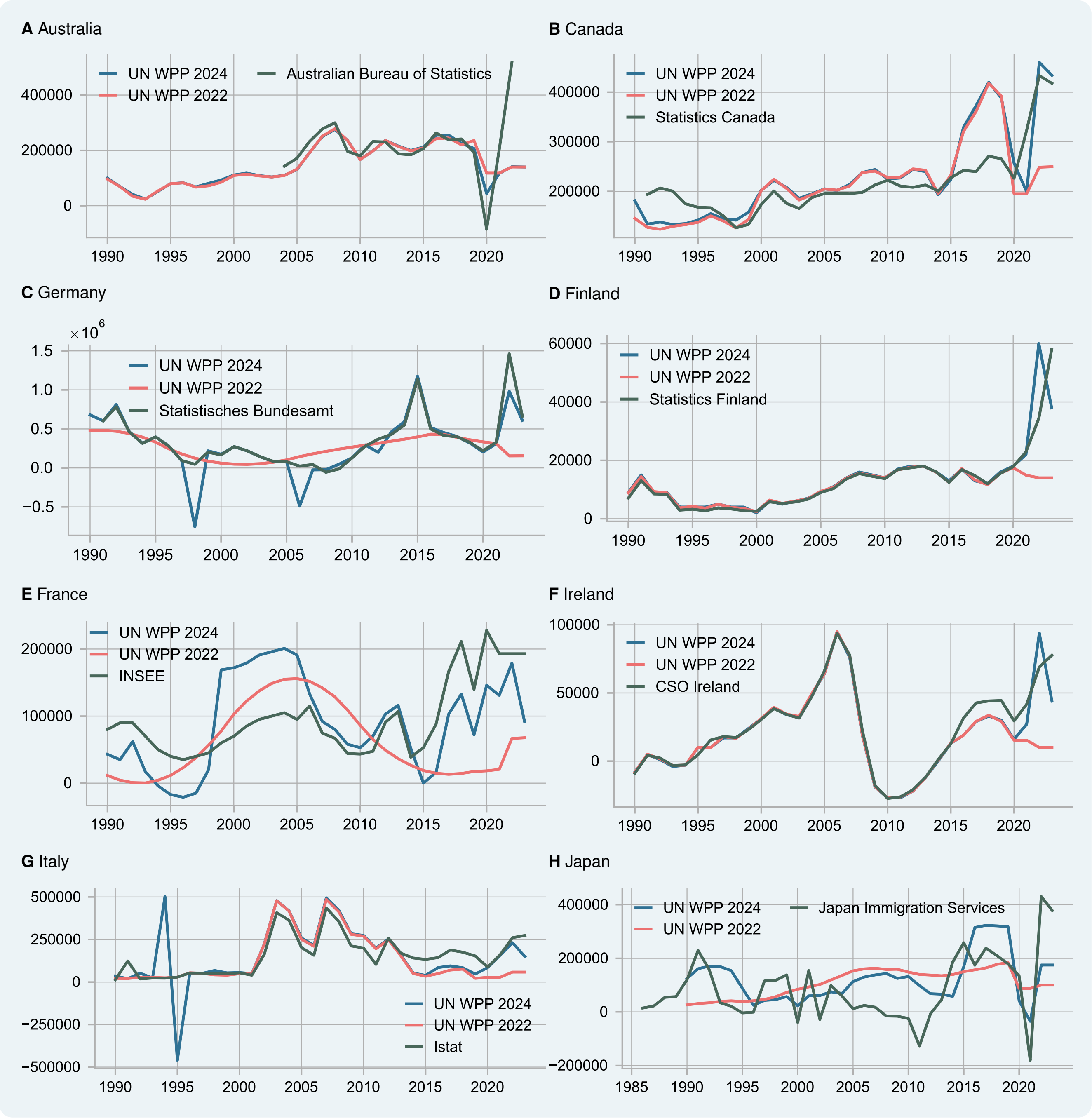

- Netherlands: data from 2003 onwards is provided by Statistics Netherlands [19] agree with the UN WPP 2024 figures, and these are thus used for the remaining years.
- New Zealand: data from 2002 onwards is provided by Statistics New Zealand [20]. Pre-2002 we use UN WPP data.
- Norway: data are provided by Statistics Norway [21].
- Portugal: data from 2002 onwards provided by Statistics Portugal [22]. Pre-2002 we use UN WPP 2024 data.

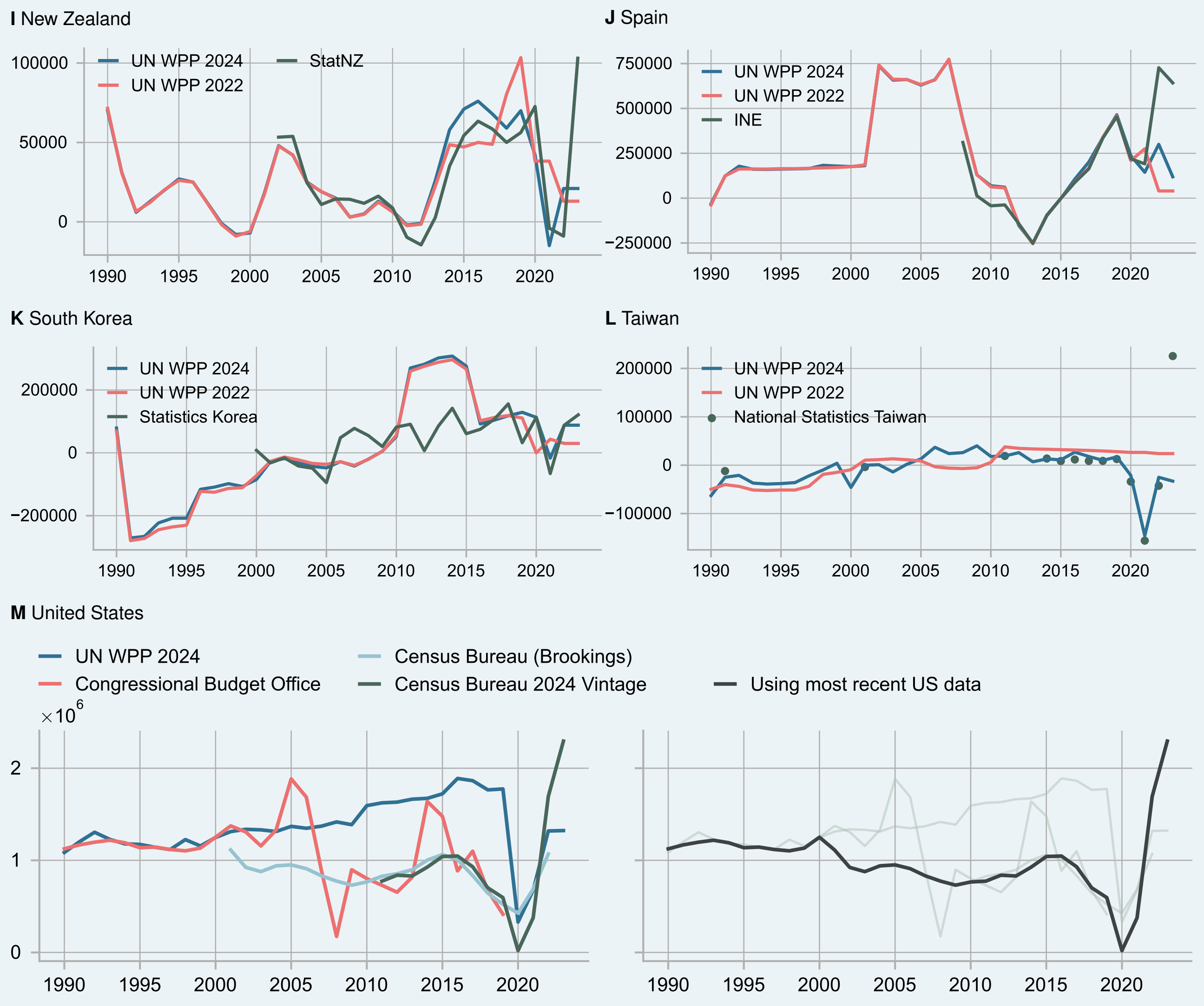

Figure S1: Selected net migration statistics from national statistical offices alongside UN WPP 2024 and UN WPP 2022 figures.

- Slovenia: data are provided by the Statistical Office of Slovenia [23].
- South Korea: Statistics Korea [24] publishes net migration figures from 2000 onwards. Pre-2000 values are not used.
- Spain: data from 2008 onwards is provided by INE (Institudo Nacional de Estadística) [25]. Pre-2008 we use UN WPP 2024 data.
- Sweden: data provided by Statistics Sweden [26].
- Switzerland: data are provided by the Bundesamt für Statistik [27] from 1991; for 1990, we use the UN WPP 2024 figure.
- Taiwan: data for some years is provided by National Statistics Taiwan [28]. Missing years are filled using the UN WPP 2024 data.

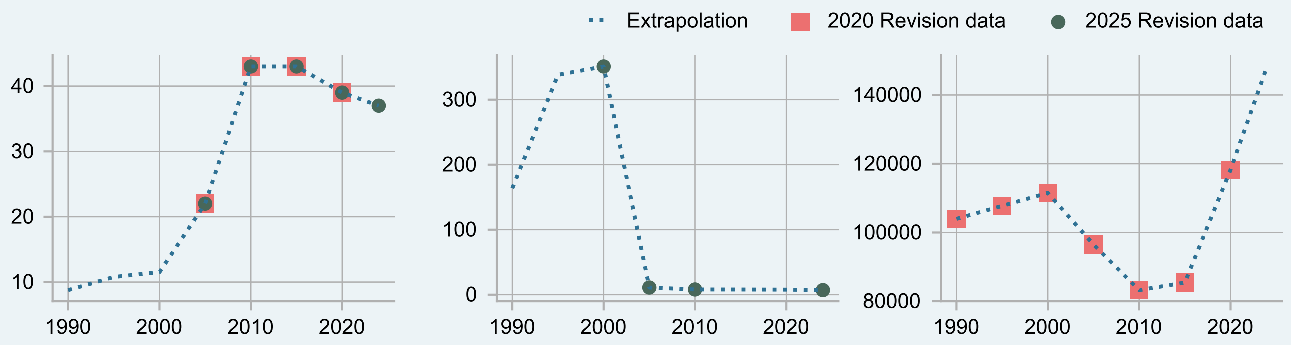

Figure S2: Three examples of stock data interpolation and extrapolation, in cases where we extrapolate back (left), forwards (right), and also interpolate intermediate values (centre). Stocks are interpolated using the weighted average growth rates of similar countries, see text. Shown are the original mid-year estimates.

- United Kingdom: data are provided by the Office for National Statistics [29].
- United States: figures from the US Census Bureau [30, 31], the Congressional Budget Office [32], and the UN WPPs offer inconsistent pictures (see Fig. S1**M**). We collate the data by always using the most recent figures from US bureaus, shown on the right.

Migrant stocks

Interpolation and Extrapolation The stocks are both a target value and an input to the neural network. We use the UN DESA 2025 Revision data where available, and fill gaps using values from the 2020 Revision. For edges (b, i, j) where stocks $S_{bi}(t)$ or $S_{bj}(t)$ are missing for *all* time points t, we set the flow $T_{bij}(t) = T_{bji}(t) = 0$ for all t if $b \neq i$ or $b \neq j$, and set the initial stock value to 0. This is problematic as it underestimates some fairly major edges (e.g. Argentina or Brazil to India), but there is no stock data available from which an inference could be made. However, there are many series $S_{bi}(t)$ where data are partially observed (i.e. values are missing for some, but not all time points t). For these series, we extrapolate missing stocks in the following way: let $c_{ij} = \mathrm{corr}(S_{bi}, S_{bj})$ and $\rho_{ij} = \exp(-d_{ij})$, where d_{ij} is the geodesic distance between country i and j. We want to extrapolate missing stock values by comparing them to 'similar' countries with complete data. 'Similarity' here means that (1) the stock time series correlate strongly, and (2) the countries are geographically close. We thus build a weight measure for a country pair (i, j) using

$$w_{ij} = c_{ij} \times \frac{\rho_{ij}}{\sum_j \rho_{ij}}.$$

Then, given a matrix of stock growth values $S_{bi}(t)/S_{bi}(t-1) - 1$, we define an average growth rate for the missing series as

$$\bar{g}_{bi} = \frac{\sum_j g_{bj} w_{ij}}{\sum_j w_{ij}}.$$

The missing stocks are then extrapolated and interpolated using this growth rate. This can be done even in the case of only a single datapoint (see Fig. S2).

After interpolation, the native-born population S_{bb} is then estimated as

$$S_{bb}(t) = P_b(t) - \sum_{i \neq b} S_{ib},$$

where $P_b(t)$ is the total population (in July of each year), taken from the UN WPP dataset. The native-born population naturally makes up the upper tail of the migrant stock distribution (see Fig. S17**H**).

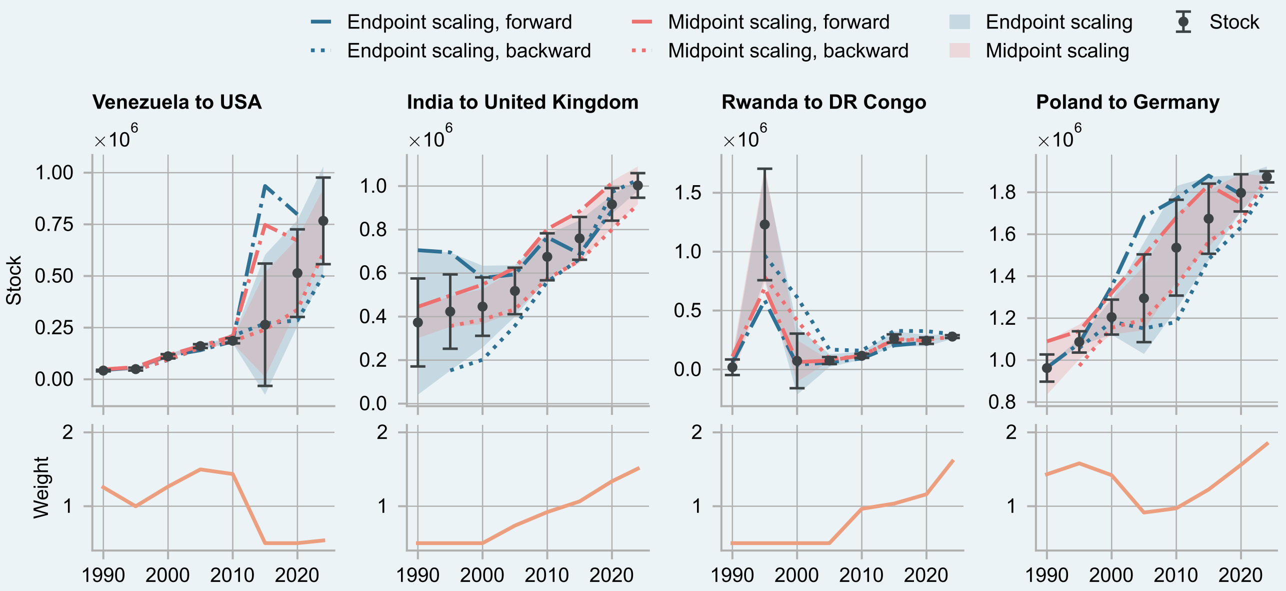

Figure S3: Calculating the weights for the stock target values. Using demographic accounting and iterative proportional fitting, between two and four new stock values can be obtained for each year ('endpoint scaling' and 'midpoint scaling'). Shaded areas indicate the average distance for each type of scaling (midpoint vs. endpoint), and the errorbars show the UN DESA value with the resulting overall error ($n = 2$ for 1990 and 2024, $n = 4$ for all other years). The average distance to the UN DESA value (at the start of year) is then used to calculate a weight (Eq. (2), bottom row) used in the loss function during training.

Estimating beginning-of-year stocks The UN DESA stocks are mid-year values, but in order to align the data with the net migration and flow values, we need to estimate the stocks at the beginning of each year. We do this using iterative procedural fitting: let $\boldsymbol{S}$ be a stock table at mid-year, $\boldsymbol{B}(t)$ the vector of total births in year t, and $\boldsymbol{\gamma}(t)$ the annual death rate in year t. To estimate beginning-of-year stocks from mid-year values, we need to account for deaths occurring in the first half of the year. Assuming deaths occur continuously and uniformly throughout the year with annual death rate γ, the probability of surviving from January to mid-year (6 months) is $(1-\gamma)^{1/2} = \sqrt{1-\gamma}$. This follows from the standard exponential survival model: if the annual survival probability is $(1-\gamma)$, then the half-year survival probability is the square root of this value. We can scale $\boldsymbol{S}$ to match the start-of-year row marginal

$$\sum_b \frac{S_{bi}}{\sqrt{1-\gamma_i}} - \frac{B_i}{2\sqrt{1-\gamma_i}},$$

where the factor of 2 is a result of assuming that births are distributed evenly throughout the year, i.e. half the births occur in the first six months of each year. The column marginals represent the total population in January of each year, as given by the UN WPP dataset [33]. Note that using a linear population decay model would lead to almost identical results, due to the equality (up to first order) of the Taylor expansions of $\sqrt{1-\gamma}^{-1}$ and $(1-\gamma/2)^{-1}$ around $\gamma = 0$.

Calculating the weights We generate demographically closed stock matrices using Iterative Proportional Fitting (IPF). Let $\boldsymbol{S}_1 = \boldsymbol{S}(t_1)$, $\boldsymbol{S}_2 = \boldsymbol{S}(t_2)$ be two successive stock tables, i.e. $t_2 = t_1 + 5$. In principle, adding the total number of births in the period $[t_1, t_2]$ to $\boldsymbol{S}_1$ and the total deaths in $[t_1, t_2]$ to $\boldsymbol{S}_2$ should result in stock matrices with the same marginals $\mu = \sum_b S_{bi}$, $\nu = \sum_i S_{bi}$. In practice, this is not the case. We can thus calculate the marginals μ_1, ν_1, μ_2, ν_2 for each stock matrix with births and deaths added, and scale each to the mid-point $\frac{1}{2}(\mu_1 + \mu_2)$, $\frac{1}{2}(\nu_1 + \nu_2)$ using IPF ('midpoint scaling'). After scaling, we subtract births and deaths again, obtaining new estimates of the stock table for each year. Doing this for each pair of stock tables, we obtain two estimates of the stock for each year except the years at the

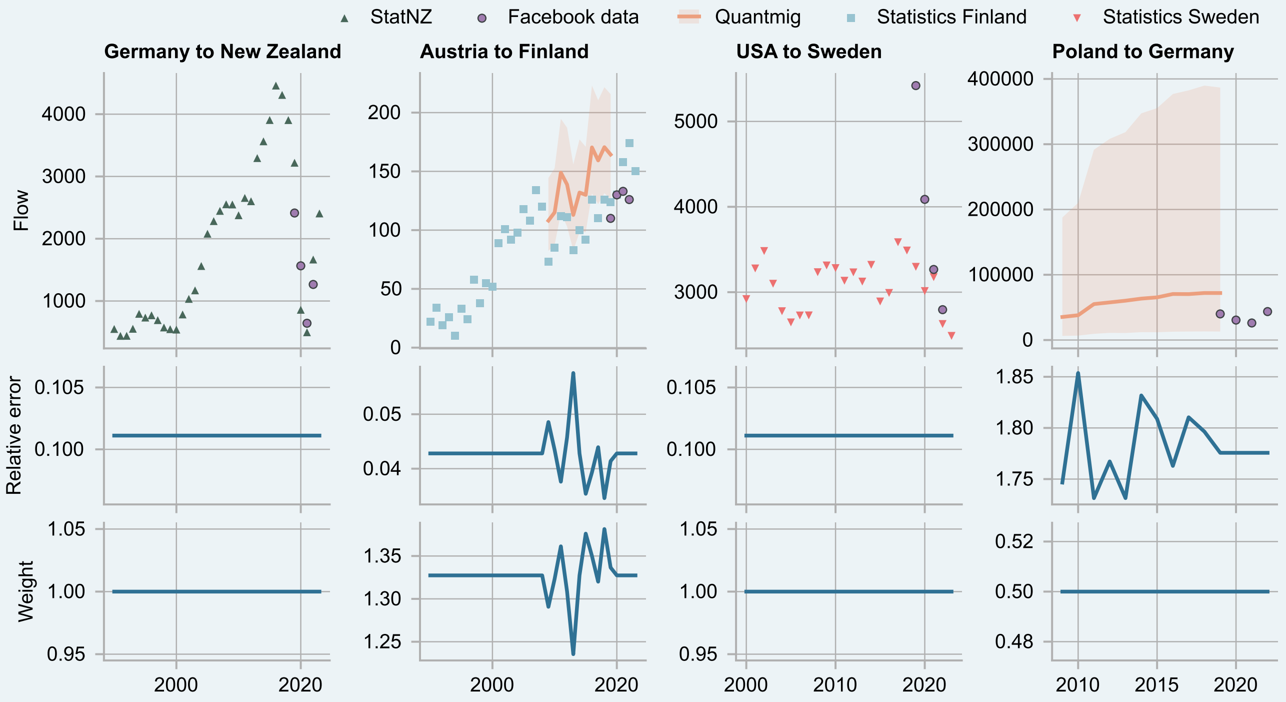

Figure S4: Calculating the weights for the flow targets. Where given, the weights are calculated from the relative error on the flow data—this is only the case for the QuantMig dataset (orange). Where uncertainty is at least partially observed, we set the weight on the remainder of the flow corridor to the median weight of the QuantMig data points (see e.g. the Austria to Finland corridor). Where the uncertainty on a flow corridor is wholly unobserved (e.g. Germany to New Zealand), the weight is set to 1. The weights are clipped to the interval $[0.5, 2]$ to ensure that no data are entirely lost or overly dominates the loss function (see e.g. the Poland to Germany corridor, which comes with significant uncertainty).

boundaries (1990 and 2024), for which we only have one; one value comes from comparison with the previous stock table ('backward') and one from comparison with the next table ('forward'). Additionally, we can also add births *and* subtract deaths *only* from S_1, and repeat the same procedure, obtaining two more estimates ('endpoint scaling'). In total, we thus obtain four additional estimates for each year except the boundary years, for which we have two (see Fig. S3). The error on each stock value is then given by

$$\sigma_{bi} = \langle |\hat{S}^k_{bi} - S_{bi}| \rangle_k, \tag{1}$$

where $\langle \cdot \rangle_k$ is the average over the various estimates obtained from IPF. The weights are calculated based on the relative error $\rho_{bi} = \sigma_{bi}/S_{bi}$, where we first normalise ρ_{bi} to have mean 0 and variance 1; this ensures that the weights are balanced around 1. We also want to avoid excessive distortion of the loss function due to extremely large or extremely small weights, and hence truncate the weights to $[0.5, 2]$. The weights w^s_{bi} used in the loss function J (Eq. (9) in the main manuscript) are thus given by

$$w^s_{bi} = \min\left(2, \max\left(0.5, \exp(-(\rho_{bi} - \langle \rho_{bi} \rangle)/\langle \rho^2_{bi} \rangle^{1/2})\right)\right), \tag{2}$$

see Fig. S5**A**. We estimate the error on a stock difference $S_{bi}(t_2) - S_{bi}(t_2)$ by assuming the errors are independent, setting $\sigma(\Delta S) = \sqrt{\sigma_1^2 + \sigma_2^2}$, and calculating the weights as above.

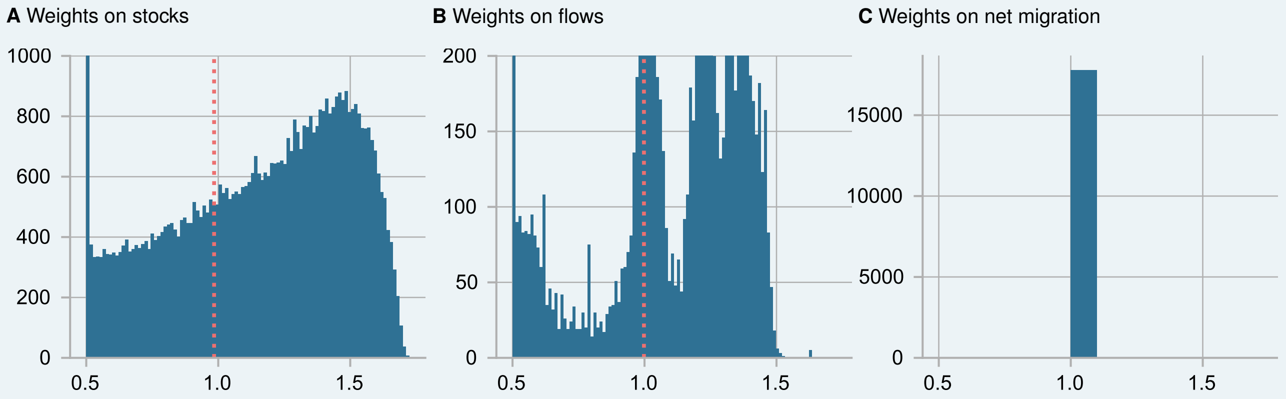

Figure S5: Distribution of the weights on the stock, flow, and net migration target values. The distributions are clipped to $[0.5, 2]$, and each have mean 1 (red dotted lines). For the net migration, we currently set all weights to 1.

Flow data

The uncertainty on the flow targets is based on the uncertainty from the QuantMig estimates, which come with standard errors. As with the stocks, the weights are then given by

$$w_{ij} = \min\left(2, \max\left(0.5, \exp(-(\rho_{ij} - \langle\rho_{ij}\rangle)/\langle\rho_{ij}^2\rangle^{1/2})\right)\right), \tag{3}$$

with ρ_{ij} the relative error (standard error divided by central estimate). For all flow corridors along which we partially know the median errors, we set the relative error to the median relative error on that corridor, and calculate the weights as above. Finally, for flow corridors with entirely unobserved errors, we set the relative error to the mean of the QuantMig dataset and the weight to 1 (see Fig. S4). Since only the QuantMig dataset provides any uncertainty estimates, the flow weights on all corridors present in the QuantMig data are essentially either 1 (meaning no information) or determined by the QuantMig flows. This approach prevents giving artificially large weights to non-QuantMig values. QuantMig values would then in effect be "punished" for coming with uncertainty, and given less weight in the dataset purely by virtue of being weighted. The downside to this approach is that projects much of the (Europe-centered) QuantMig methodology onto other values.

In Fig. S6 we show the geographic distribution of the target data, as well as the proportion of each source. Shown are total numbers of data values, not the actual sum of the values themselves—a single target value thus counting as one. Stock and flow data have global coverage; the former are markedly more dense across Europe, North America, and Oceania, partly due to better coverage and more efforts by UN DESA to obtain data for these regions, and partly due to a higher diversity of migrant populations. Flow data is primarily given by the Facebook flows—about 45% of all targets and more than half of all flow data—with QuantMig and the three national statistics making up around 20% of all targets, see Fig. S6**F**. In panel S6**C** we see the distribution of the net migration targets (totalling around 1,000, or 0.4% of the total target dataset), and panel S6**D** gives the distribution of the number of all target data values. As can be seen, Europe is the region with the overall highest number of target values—almost double that of the other regions. In total, about 198,000 datapoints relate to the global North (Europe, North America, Oceania, Japan, South Korea), and 276,000 to the global South. About half of stocks and flow values are North-South flows, i.e. connecting a country of the global South and a country of the global North; a third a South-South flows and stocks, and fewer than a fifth are North-North stocks or flows.

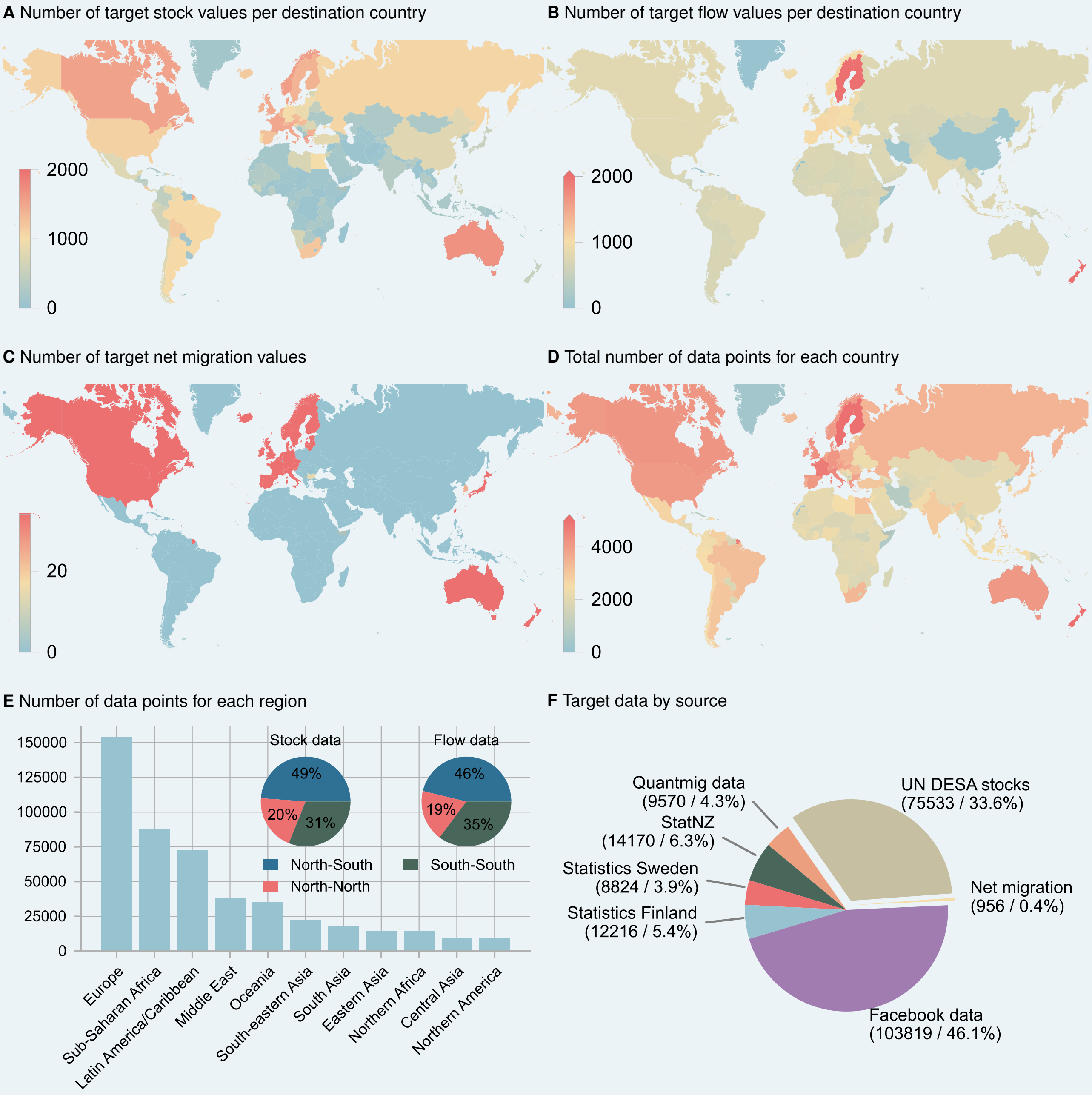

Figure S6: Geographic distribution of the target data, as well as the proportion of each dataset in the target data. Shown are statistics for the number of individual data points for each country, regardless of their absolute value. **A** Total number of target stock values for each destination country. **B** Total number of flow target values for each destination country; note the logarithmic scale, since Sweden, New Zealand, and Finland are disproportionately represented. **C** Total number of target net migration values for each country. **D** Total number of data points for each country; the bilateral stock and flow is counted towards both origin and destination countries. **E** The data shown in panel D, aggregated for each geographic region. Shown in the inset is the categorisation of bilateral stock and flow data by development level; North-North data points only connect regions of the global North (Europe, North America, South Korea, Japan, Oceania); South-South data points only regions of the global South. **F** The makeup of the target data by source; shown are the total number of data points used from each source, as well as the proportion of the total.

Input data

GDP per capita and GDP annual growth

In this section we detail the assembly of the economic covariates used to train the neural network: GDP per capita, in real 2015 USD; and GDP % annual growth, given by

$$\partial_t \mathrm{GDP}(t) = 100 \times \left(\frac{\mathrm{GDP}(t)}{\mathrm{GDP}(t-1)} - 1 \right). \tag{4}$$

GDP data are sourced from the World Bank [34, 35] as well as UNCTAD [36]. However, these datasets are not complete for all countries and years required. Historical GDP growth rates can be calculated from the Maddison dataset [37] (giving GDP/capita in 2011 PPP) and the IMF dataset [38] (GDP/capita in 2021 PPP). Note that GDP growth can be calculated from any measure of real GDP (e.g. constant USD or constant PPP). GDP growth, when available, can be used to calculate GDP/capita for missing years. At times, only nominal GDP figures are available for a given year and country. Converting to real GDP requires the *deflator* for that country,

$$\text{GDP deflator}(t) = \frac{\text{nominal GDP}(t)}{\text{real GDP}(t)}. \tag{5}$$

Where the deflator is unavailable, deflators from economically, geographically, or socially similar countries can be used to estimate real GDP. We also fill missing values using figures from national statistical bureaus and additional sources, as below.

GDP data are scaled using a Yeo-Johnson transform (see Fig. S17**A**–**B**).

Caribbean Netherlands The Netherlands Antilles were a constituent country of the Kingdom of the Netherlands, consisting of the islands of Bonaire, St. Eustatius, and Saba (the BES islands), Aruba, Curaçao, and Sint Marteen. Aruba became an independent constituent country in 1986, and in 2010, Curaçao and Sint Marteen followed suit, at which point the Netherlands Antilles were dissolved and the remaining BES islands became a special municipality within the Kingdom. The World Bank and UNCTAD provide real GDP figures for Aruba from 1986, for Curacao from 2000, and for Sint Marteen from 2009. Statistics Netherlands [39] provides nominal GDP for the BES islands from 2012–2022, while UN DESA provides nominal GDP for the entire Netherlands Antilles from 1970–2009 [40], which agrees with the World Bank data for Curaçao, the largest of the remaining Antilles islands after 1986 (see Fig. S7**A**). Since the deflators are not available, we convert the UN DESA data to real GDP using the deflator for Aruba, and then extrapolate back to 1986 using the growth rate for Aruba (see Fig. S7**B**).

French overseas territories Statista [41] provides real GDP in constant 2014 Euros for the period 2000–2022. We convert to 2015 USD and extrapolate back to 1989 using the average growth rate for each territory, calculated over the period from 2000–2008. We extrapolate forward to 2023 using the average growth rate for each country in the period 2017–2022 (see Fig. S8).

Eritrea Eritrea formally achieved independence from Ethiopia in 1991. UNCTAD provides data from 1992, and we extrapolate back to 1989 using the growth rate from Ethiopia (see Fig. S9).

Small Pacific Islands The World Bank and UNCTAD datasets have missing data for American Samoa, Niue, Tokelau, Wallis and Futuna, Guam, and the Northern Mariana Islands. The Pacific Community provides nominal GDP figures [42], as well as country-specific inflation rates [43], which we use as a proxy for the GDP deflator. We then extrapolate back using growth rates of neighbouring countries:

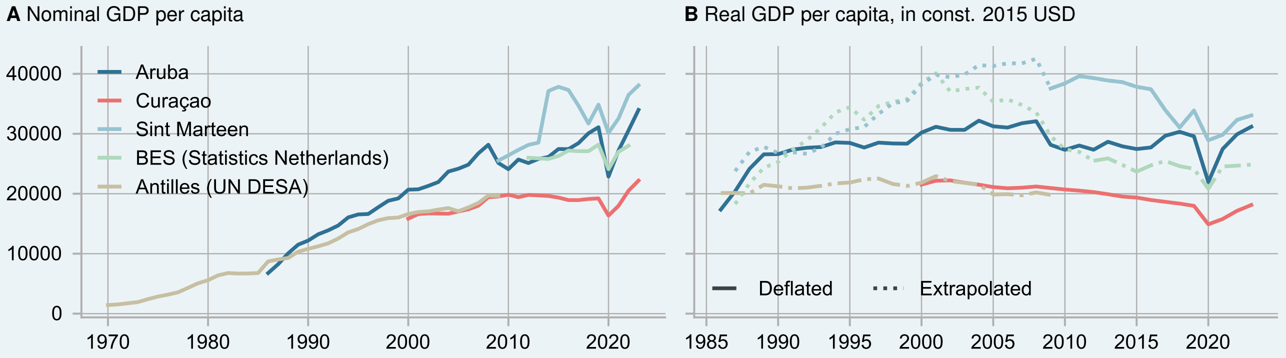

Figure S7: Caribbean Netherlands. **A** Available nominal GDP per capita figures for the constituent islands. **B** We fill missing years by deflating nominal GDP figures using the deflator for Aruba, and then extrapolate back using the growth rate from Aruba.

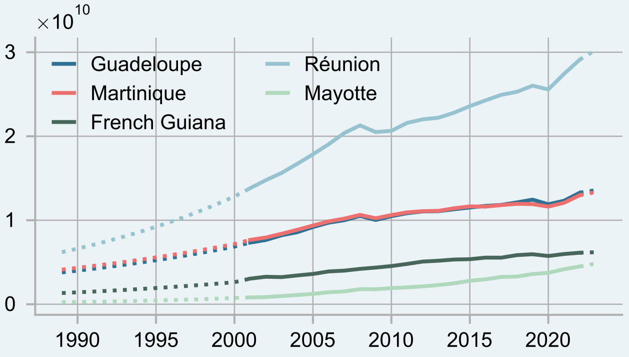

Figure S8: French overseas territories real GDP, in 2015 USD. The available data from Statista is extrapolated to the entire period 1989–2023 using the average growth rates for each country.

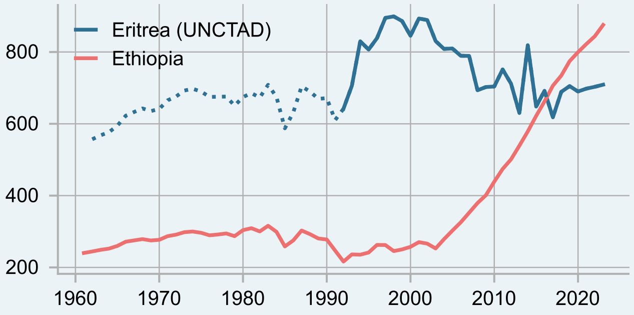

Figure S9: Eritrea. Since the country did not formerly achieve independence from Ethiopia until 1991, we extrapolate the available data from UNCTAD back to 1989 using the Ethiopian growth rate (dotted line).

1. American Samoa, Niue, and Wallis-Futuna: average growth rate of New Caledonia, the Cook Islands, and Fiji,
2. Tokelau: growth rate of Fiji.

US overseas territories Guam and the Northern Marianas are US overseas territories that to a large part rely on US funding for their economies. We extrapolate both Guam and the Marianas using the growth rate from Puerto Rico. The US Virgin Islands we extrapolate back using the average growth rate of Puerto Rico, St. Kitts and Nevis, and Antigua and Barbuda.

Serbia and Montenegro The state of Serbia and Montenegro existed from 1992 until 2006, at which point the two constituent republics separated and became independent nations. Growth rates for these two countries are estimated in the Maddison project dataset, and are used to extrapolate GDP figures back to 1960 (see Fig. S12).

South Sudan South Sudan ceded from Sudan in 2011. UNCTAD gives real GDP figures from 2011 onwards, and we extrapolate back in time using the growth figures from Sudan (see Fig. S13).

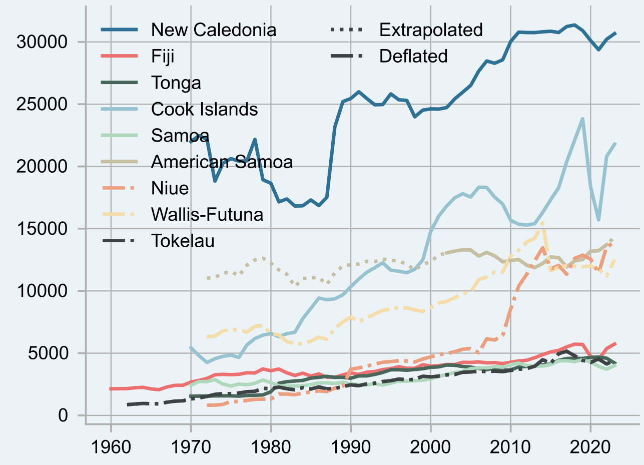

Figure S10: Small Pacific Islands GDP per capita, in const 2015 USD. Nominal GDP data from the Pacific Community data are deflated using the inflation rate as a proxy (dash-dotted lines). We then extrapolate back (dotted lines) using growth rates from neighbouring countries with similar GDP/capita.

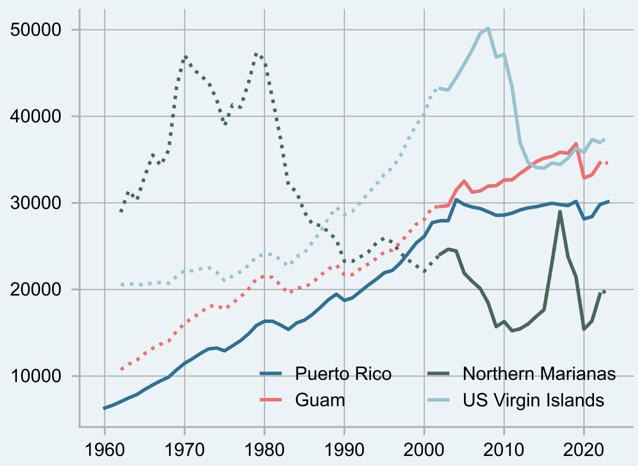

Figure S11: US overseas territories GDP per capita, in const 2015 USD. Missing values for Guam, the Northern Marianas, and the US Virgin Islands are extrapolated back using the growth rate of Puerto Rico (dotted lines).

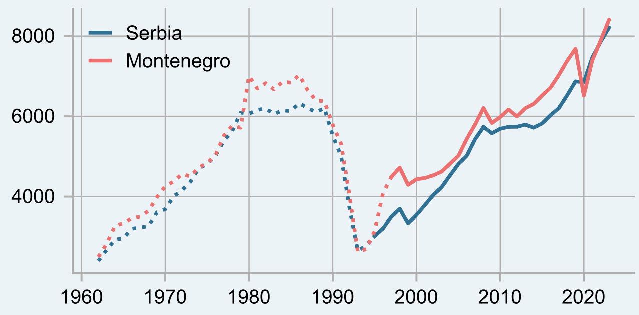

Figure S12: Serbia and Montenegro, GDP per capita in const 2015 USD. We extrapolate back to 1960 using the growth rates given by the Maddison project dataset (dotted lines).

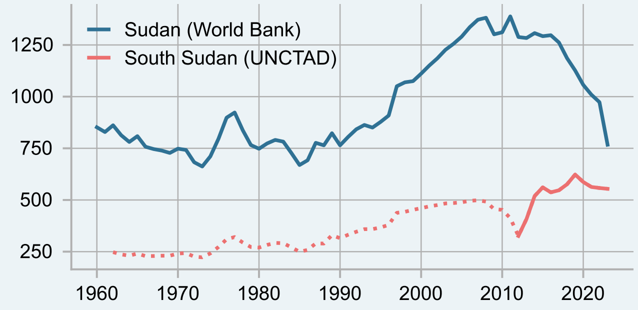

Figure S13: Sudan and South Sudan GDP per capita in const 2015 USD. We extrapolate the UNCTAD data for South Sudan back from 2011, the year of its independence, using the growth rate of Sudan (dotted line).

East Timor The only missing data point is the GDP growth rate for 1990. A report by the Australian government [44] suggests an average 10% economic growth rate from 1987–1997, due to heavy investment in construction. Given the data we already have, this implies GDP growth from 1987-1990 must have averaged 11.7%.

Western Sahara, Saint Pierre Miquelon No data are available for these territories. Western Sahara is under Moroccan control, and Saint Pierre Miquelon is a small French territory located near the Canadian province of Newfoundland. We thus simply use Moroccan and Canadian GDP per capita figures respectively.

British overseas territories and Crown Dependencies Four British territories have missing data: the Isle of Man, Gibralatar, the Falkland Islands, and Saint Helena, Ascension and Tristan da Cunha (in the following simply referred to as St. Helena), a small island territory in the Atlantic ocean. The Isle of Man government [45] provides the missing real GDP growth figures for 2022 and 2023, from which we can infer the missing real GDP/capita figures for those years. The government of Gibralatar [46] provides nominal data from 2009; we deflate using the British deflator as a proxy, and extrap-

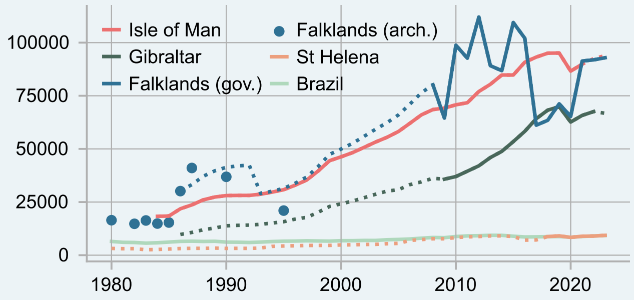

Figure S14: British territories real GDP per capita in const 2015 USD. Missing data for Gibraltar and Falklands is extrapolated back from Government data using the Isle of Man growth rate (dotted lines). The Falklands extrapolation agrees broadly with archival data (blue dots). Government data for St. Helena is extrapolated back using the growth rate for Brazil, also shown for comparison purposes.

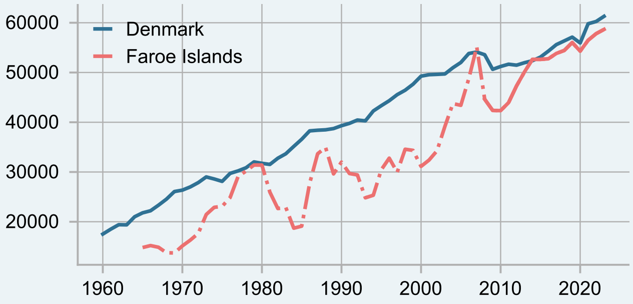

Figure S15: Faroe Islands real GDP per capita in const 2015 USD. Missing data for the islands is calculated by deflating the available nominal figures using the Danish deflator as a proxy (dash-dotted line).

olate back using the Isle of Man growth rate. Data for the Falklands real GDP is available from the government website, given at constant 2012 Falklands pounds (pegged to the British pound at an exchange rate of 1:1) [47]. We extrapolate back using the Isle of Man growth rates. Some historical data from 1980–1995 is available from the archives [48], which, when converted to real 2015 USD, agrees well with this extrapolation (see Fig. S14). Lastly, nominal GDP data are provided by the St. Helena government website up to 2017. We use the British deflator to convert to real GDP and extrapolate back using the GDP growth rate for Brazil, a nearest neighbour with a similar GDP per capita.

Faroe Islands The World Bank provides real GDP/capita from 2008 onwards, and nominal GDP/capita from 1965 onwards, which we deflate using the Danish deflator to obtain a complete time series, see Fig. S15.

Bilateral trade

Trade flows are taken from the BACI harmonised tradeflow dataset [49, 50], and extrapolated back using the real growth rates calculated from the UN Comtrade and IMF Direction of trade statistics as given in the CEPII gravity dataset [51]. Since each dataset contains both origin- and destination-reported trade flows, we take the average growth rate (see Fig. S16). Where values are missing, we simply set the trade flow to the last known value. If all values are NaN, we set the flow to 0.

Total population, Life Expectancy, Birth and Death rates

We include the total population of the country of birth, country of origin, and country of destination for each edge (b, i, j). These figures are taken from UN WPP dataset and scaled using a Yeo-Johnson transform (see Fig. S17**C**–**F**).

Distance

We use the `distw_harmonic` distance covariate from the CEPII dataset as a measure of distance. Since values for some years are missing, we take the average over the period 1990–2023. Values for the British Virgin Islands and the Isle of Man are missing; we approximate these by using the values for the neighbouring US Virgin Islands, and inserting a value of 80km for the distance US Virgin Islands–British Virgin Islands. For the Isle of Man, we use distance values for the United Kingdom and insert a value of 300km for the distance United Kingdom–Isle of Man. The covariate is then scaled using a Yeo-Johnson transform, see Fig. S17**G**.

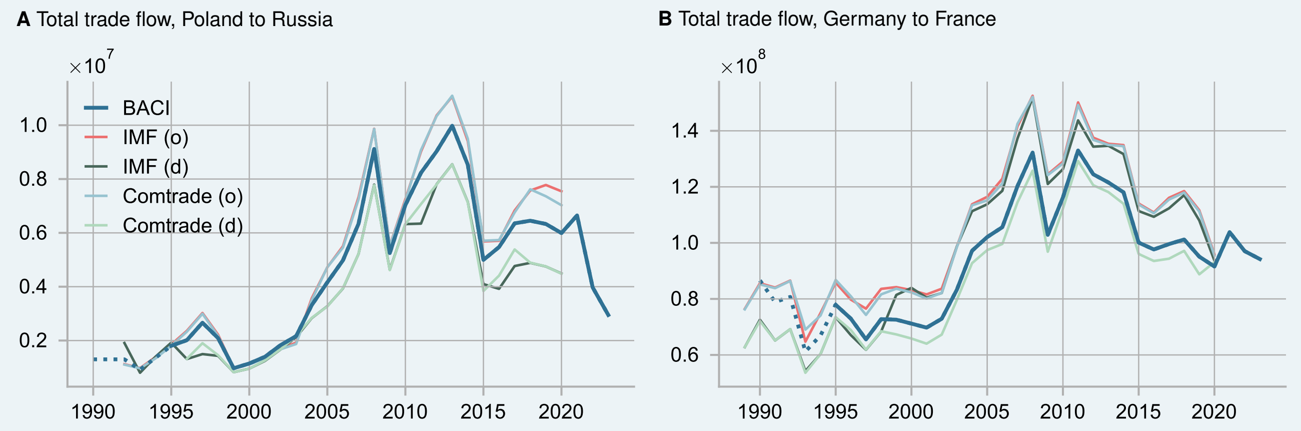

Figure S16: Two tradeflow examples, showing total flow in constant 2015 USD. Shown are the BACI figures (solid blue line), as well as the Comtrade and IMF figures. Missing figures in the BACI dataset are extrapolated back using average growth rates (dotted lines).

Linguistic similarity

Linguistic similarity is taken the USITC Domestic and International Common Language Database [52]. We use the `csl` (common spoken language) index. As detailed in the dataset description, values for 46 countries are missing. Following the authors, for these cases we use the average of the linguistic proximity `lpn` and common native language `cnl` instead.

Religious similarity

The Correlates of War database [53] details the religious composition $\boldsymbol{\alpha}$ of each country, that is the share of adherents to major religions in each country. Missing values are taken from the CIA World Factbook [54]. The religious similarity between two countries is then simply $\langle \boldsymbol{\alpha}_i, \boldsymbol{\alpha}_j \rangle$, where we exclude the 'other' (`othrgenpct`) category. This gives a more accurate picture of religious proximity than the CEPII `comrelig` covariate, which only uses the share of Catholics, Protestants, and Muslims to construct a similarity score.

EU Membership

This is taken from the CEPII gravity dataset. We construct two covariates, EU_{ij} and EU_{jk}, which are 1 if the indexed countries are both EU members, 0 else. As a binary covariate, it is left unscaled.

Colonial ties

Colonial relations between two countries are taken from the USITC gravity dataset (`colony_of_destination_ever`). We include two covariates, COL_{ij} and COL_{bj}, which are 1 if i (or b) was ever a colony of the destination j, and 0 else. As a binary covariate, it is left unscaled.

Validation on synthetic data

We validate our approach on a small synthetic dataset of migration flows between 30 destination and origin countries over 10 years. This is both to ensure our model is able to in principle infer the full flow table from observations of the stocks and flows, as well as to tune the hyperparameters. To do this, we select 30 random countries from the period 2010–2020,

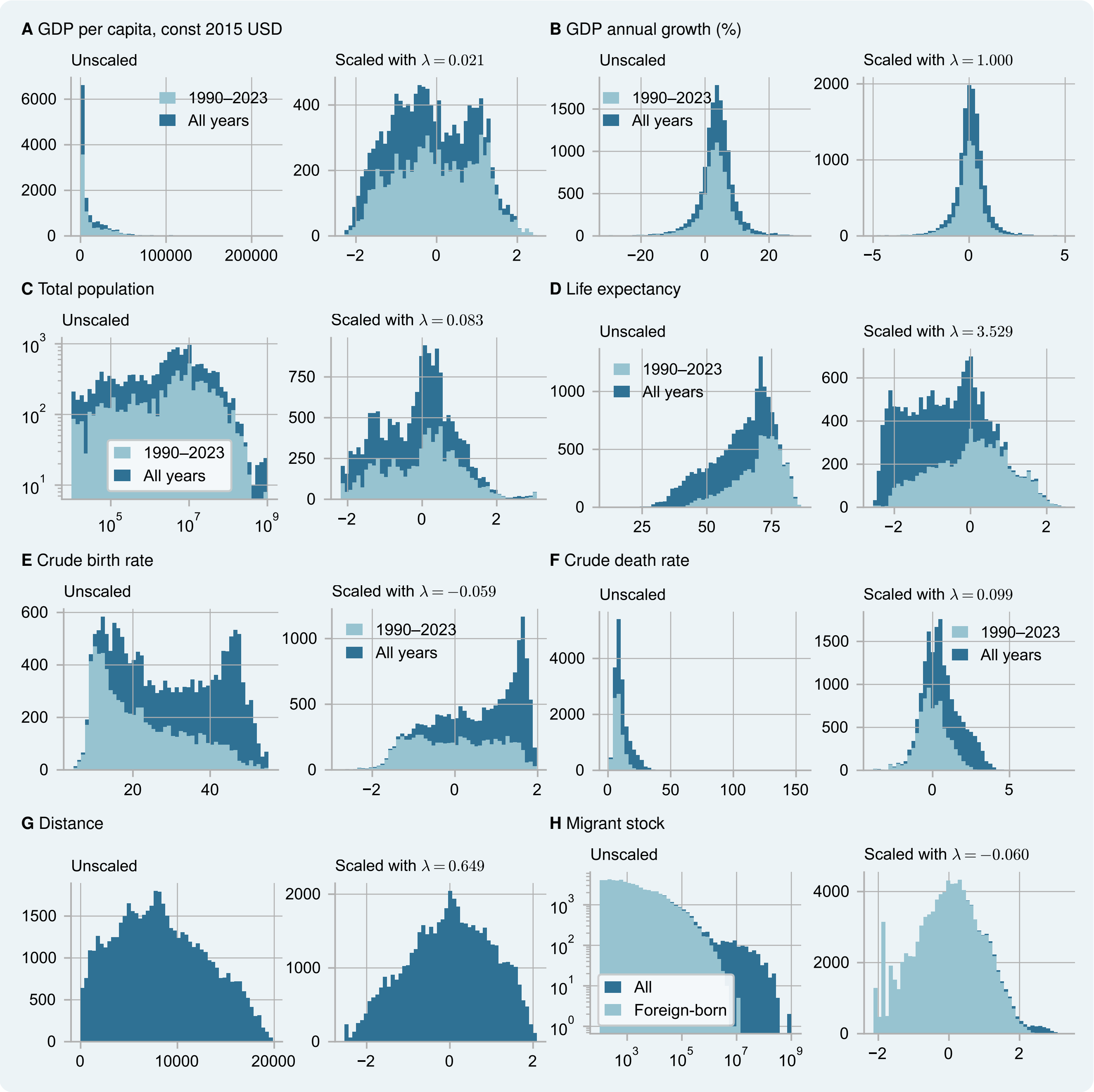

generate a random initial stock table $\boldsymbol{S}(t_0)$, and generate flows from

$$T_{bij} = \eta \exp(\langle \boldsymbol{\chi}_{bij}, \boldsymbol{\alpha} \rangle), \tag{6}$$

where $\boldsymbol{\chi}$ are the Yeo-Johnson-transformed covariate vectors, $\eta = 100$ a scalar, and $\boldsymbol{\alpha}$ a vector of random coefficients drawn from an i.i.d uniform distribution, $\alpha_i \sim \mathcal{U}[0, 0.5]$. Note that the vector $\boldsymbol{\chi}$ contains the stocks S_{bi} and S_{bj} themselves, as in the full model; these are calculated recursively from $\boldsymbol{T}$. This produces a full synthetic dataset of stocks, flows, and net migration vectors on which we can validate the approach. We first assume all flow, net migration, and stock values are observed, and train the neural network for 10,000 epochs. We use a 3-layer neural network with 20 neurons in the hidden

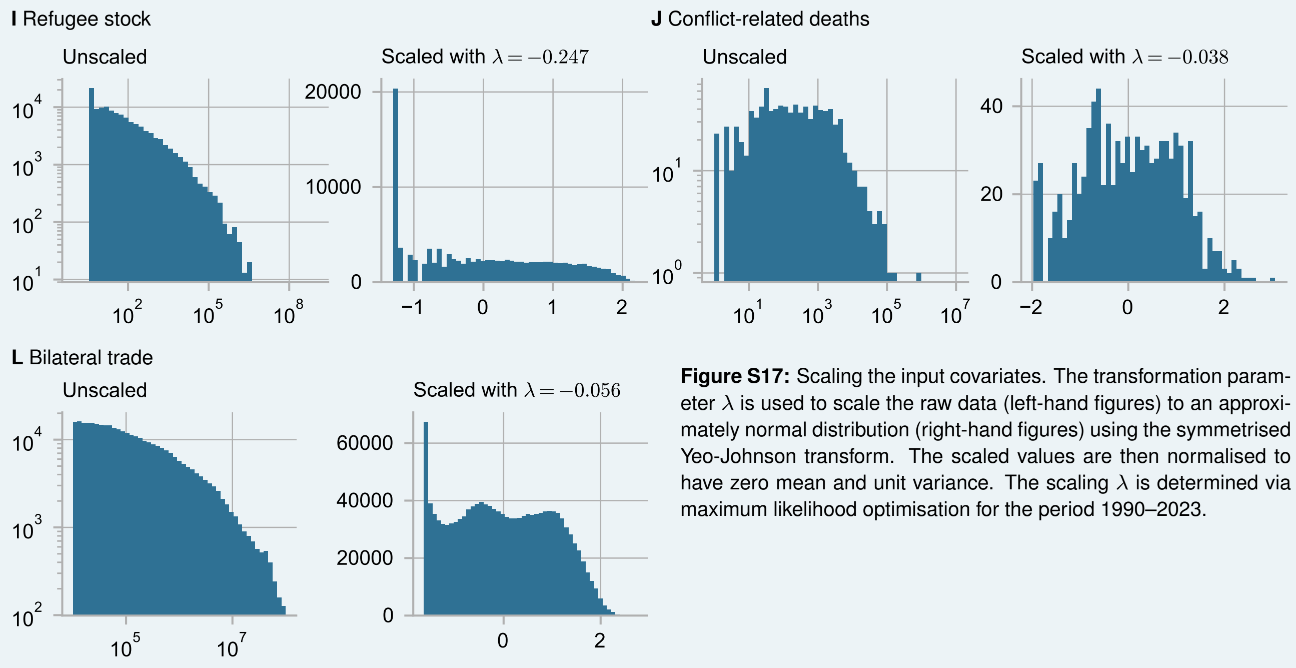

Figure S17: Scaling the input covariates. The transformation parameter λ is used to scale the raw data (left-hand figures) to an approximately normal distribution (right-hand figures) using the symmetrised Yeo-Johnson transform. The scaled values are then normalised to have zero mean and unit variance. The scaling λ is determined via maximum likelihood optimisation for the period 1990–2023.

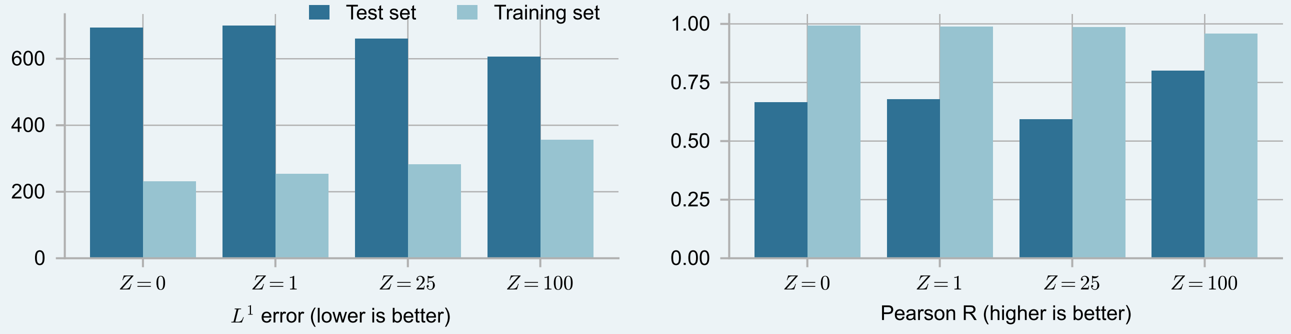

Figure S18: Adjusting the dimension Z of the latent state $\boldsymbol{z}$. $Z = 0$ indicates a regular feed-forward architecture with no recurrent structure. The far left, the average L^1 error on the test and training set. Right: average correlation test and training set.

layers, and the same CELU (continuously differentiable exponential linear unit) activation function on the final layer as in the main manuscript [55]. We vary the choice of activation function in the hidden layer. As shown in Fig. S19, the full flow table $\boldsymbol{T}$ is then uniquely inferred from the data, though accuracy varies with the choice of activation function. Naturally, since we are observing the full flow table $\boldsymbol{F}$, the net migration data are fully determined by $\boldsymbol{F}$ and thus redundant.

To generate a more realistic dataset, we repeat the above process, adding 10% (multiplicative) noise to the stocks, 20% noise to flows, and 5% noise to the net migration data. We then randomly mask out 80% of flow corridors (that is, entire origin-destination corridors), 80% of net migration corridors, and 10% of individual stock values (not corridors). To tune the hyperparameters of the model, we perform a grid search on the depth, width, and activation functions of the network, and compare prediction accuracy on the true flow table $\boldsymbol{T}$ (uncorrupted by noise) after 10,000 training epochs. Results are shown in Fig. S20, indicating that the hyperbolic tangent activation function, together with a high number of layers, gives best prediction results.

To select the dimension of the latent space, we select 20% of flow corridors uniformly at random, and include only the

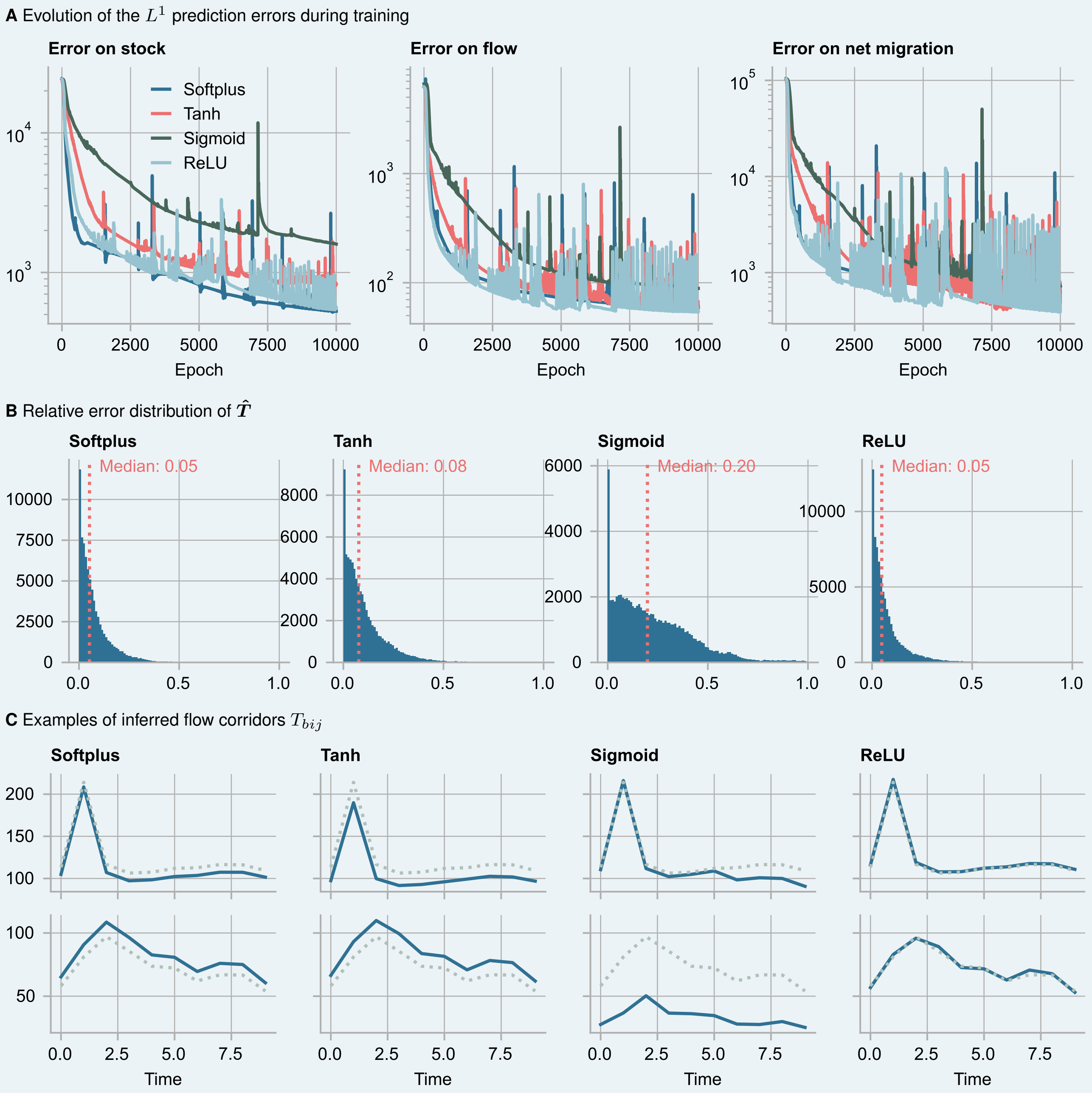

Figure S19: Validation of the neural network approach on noiseless, fully observed synthetic data. In all cases, we use a 3-layer neural network with 20 neurons per layer, but vary the activation function on the hidden layers. **A** The evolution of the error on the stocks, flows, and net migration as the models train. All models converge to the true target data, though at different rates. **B** The distribution of the relative error on the full table $\hat{\boldsymbol{T}}$ after 10,000 epochs. The median error is indicated. **C** Predicted and true (dashed) values T_{bij} on two randomly selected edges. After 10,000 epochs (roughly 2 minutes of training on a GPU), the best-performing models have largely already converged to the true solution $\boldsymbol{T}$.

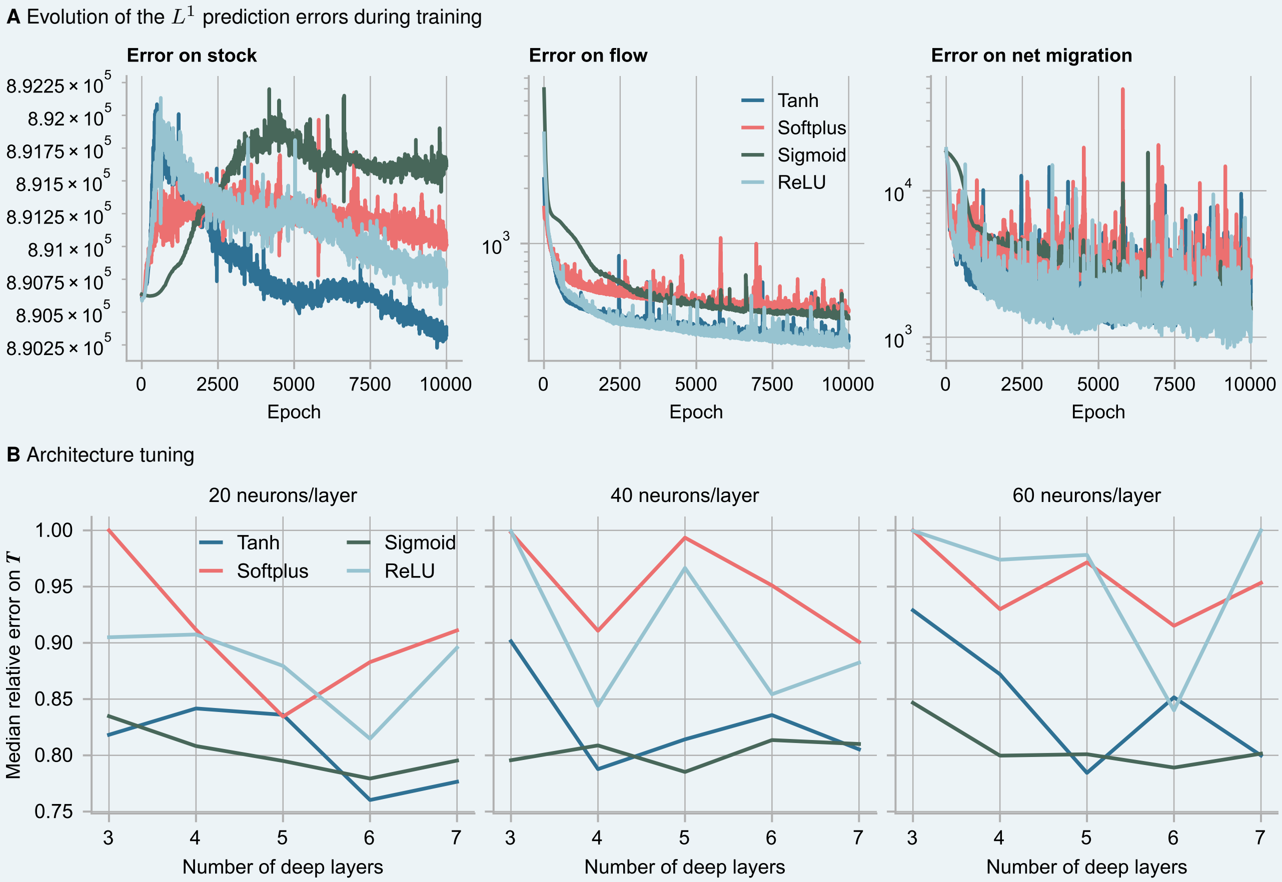

Figure S20: Tuning of the neural network hyperparameters on noisy, partially observed synthetic data. **A** The evolution of the error on the stocks, flows, and net migration is now noticeably slower and more jagged than in the noiseless case. **B** Comparison of the median relative error on $\hat{\boldsymbol{T}}$ for various architectures.

remaining 80% in the training data; then we train an ensemble of neural networks with different latent space dimensions Z and compare performance. Fig. S18 shows the L^1 error on the test set, the training loss, the average correlation on the test set, and the distribution of correlations on the flow corridors contained in the test set. As can be seen, a high latent space of dimension of 100 performs best of all the dimensions considered.

Finally, we set the Yeo-Johnson scaling parameters to a value of $\lambda = 0.5$ for all target datasets, based on the following heuristic. The 0.5 value, intuitively, lies 'half-way' between the identity transform $\lambda = 1$, and the logarithmic transform $\lambda = 0.0$. In Fig. S21 we show the learned flow values on the noiseless datasets for different values of the transformation parameters, suggesting that values between 0.5 and 0.7 are optimal. Small values of λ penalise all relative errors more equally, meaning a relative error of 50% on a flow of 100 contributes more to the loss than an error of 10% on a flow of 1 million—though in absolute terms these errors are of course orders of magnitude apart. For small λ, the model tries to optimise all targets equally well (or, in practice, equally badly). Large values of λ, conversely, mean that small flows are disregarded, and only the large values optimised. Centre values around $\lambda = 0.5$ balances these considerations.

Figure S22 shows the correlation metrics from Extended Data Fig 4 for the five folds used for cross-validation.

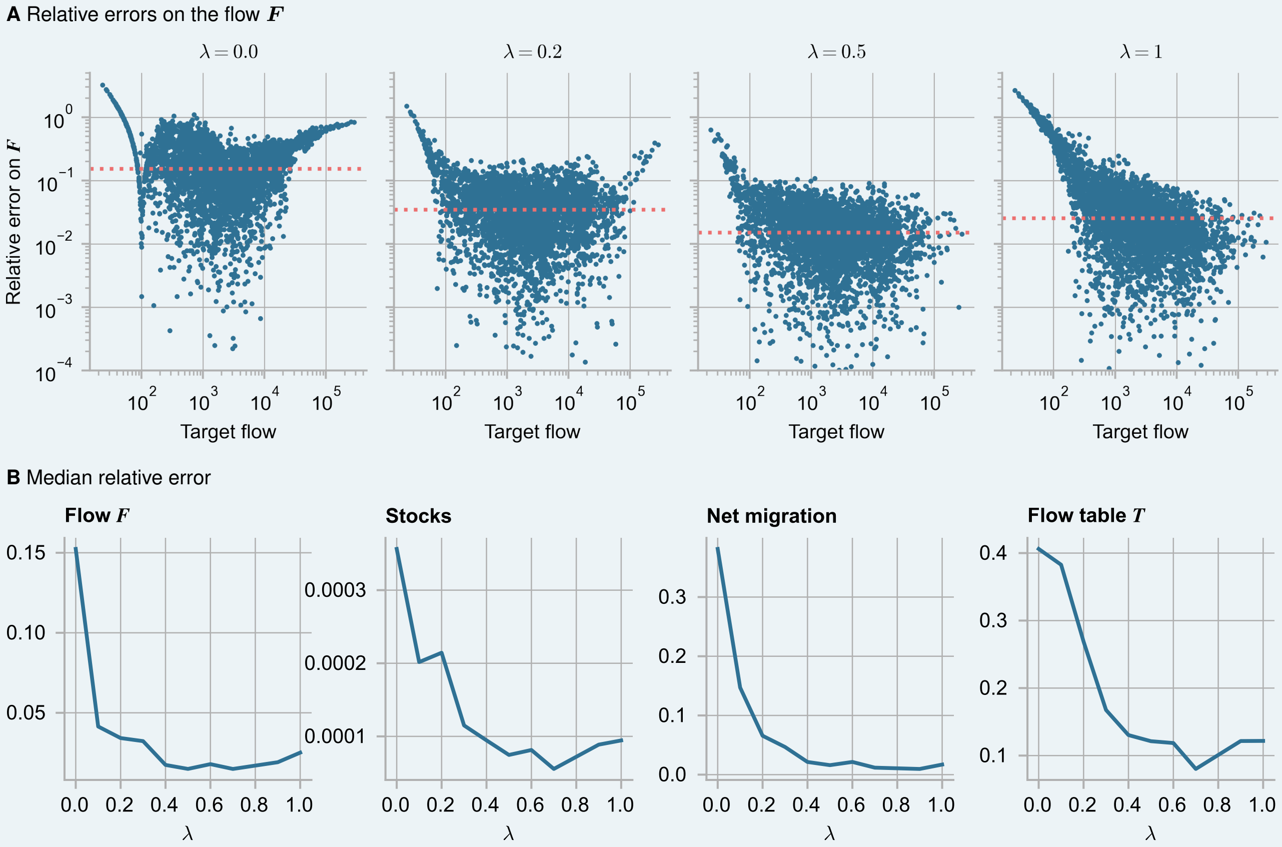

Figure S21: Tuning the Yeo-Johnson scaling parameter λ. **A** A value of $\lambda = 0.5$ balances the relative errors across different orders of magnitude of the target value. If λ is too small, all values are penalised equally, leading to poor performance across all orders of magnitude. A large value of λ leads to poor performance on small flows. The median relative error (red dotted line) is optimal for values around $\lambda = 0.5$. **B** Median relative error on all four datasets as a function of λ.

Analysing the bias

In this section, we present a number of additional numerical experiments we performed in order to assess data leakage of migration patterns across developmental regions. Fig. S6 shows that a significant portion of training data originates from high-income countries, and it can further be assumed that those data are of generally higher quality than for low-income regions. Even though the Finnish, Swedish, New Zealand, and QuantMig datasets only make up around 20% of target values, Europe remains the region with the highest number of data points (around 150,000)—almost twice as many as the second-largest region (Sub-Saharan Africa, 87,000).

In Figure S23**A** we show the variance on the net migration estimate $\hat{M}$ as a function of the number of data points available for each country. Since $M_j = \sum_{b,i} T_{bij} - T_{bji}$ and T_{bij} can be assumed i.i.d. Poisson distributed with variance λ_{bij}, we have

$$\text{var } M_j = \sum_{b,i} \lambda_{bij} + \lambda_{bji},$$

hence it is reasonable to expect the variance on the net migration to also reflect the uncertainty on the flows. As shown, the relative uncertainty on $\hat{M}$ drops by about 0.02% per data point added, demonstrating that the uncertainty on our estimator reflects the range of possible migration flows that plausibly fit the observations.

In order to assess the degree of Northern migration patterns being projected onto the South, we compared the test

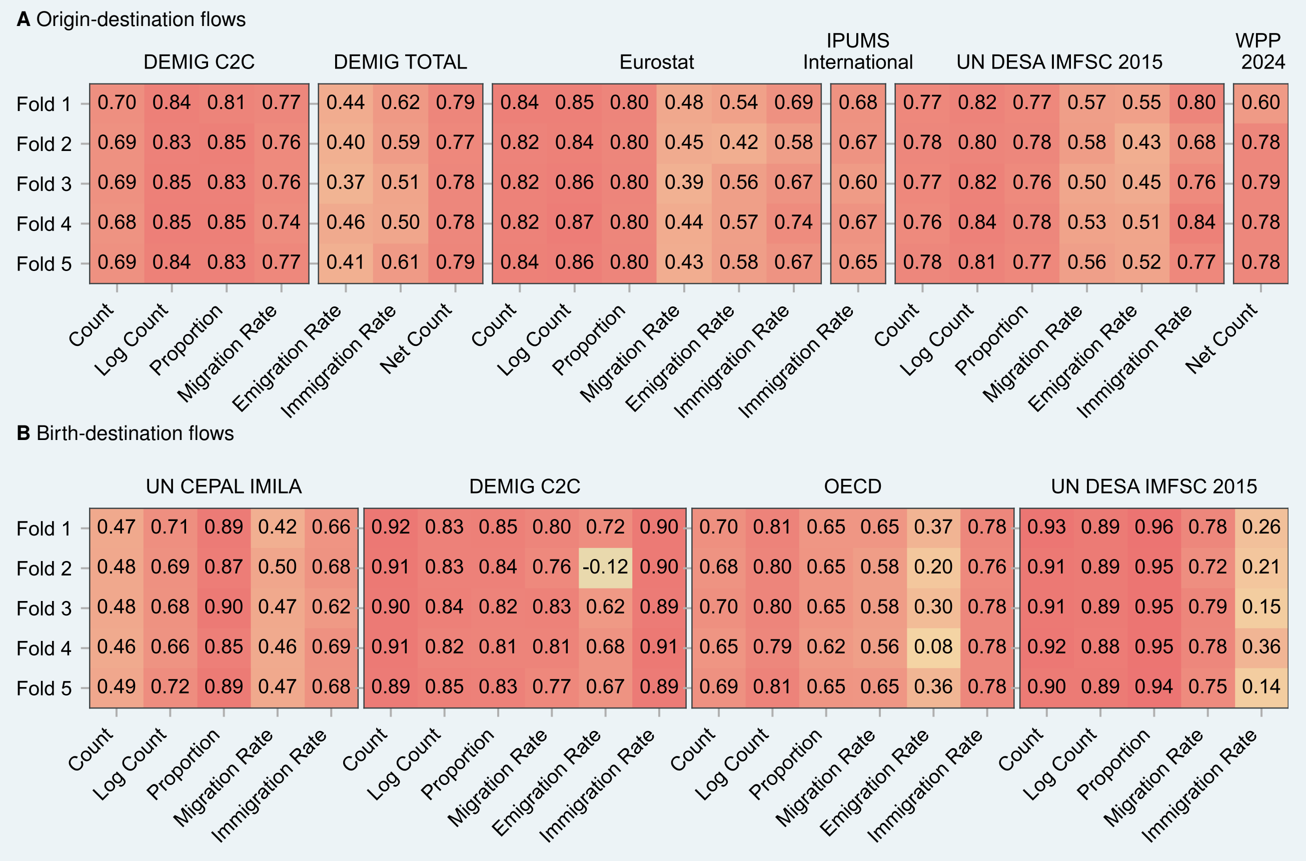

Figure S22: The same plots as in Extended Data Fig. 4 for the five folds used for validation.

performance of our model on the different developmental regions. In Fig. S23**B**, we show the prediction performance on each fold used to validate the model by developmental region. North-South flows are reproduced the most accurately, followed by South-South flows. While it at first glance may appear surprising that North-North flows are significantly *less* accurate, there is in fact a simple explanation: this is the region for which we have several conflicting data sources, and the neural networks must therefore 'balance' more information than for the global South, for which we are broadly relying on stocks and Facebook flows. Performance on South-South flows is typically around 15-20% worse than for North-South flows, which may be a reflection of the lower training data quality, but could also indicate data leakage.

To further investigate this question, we trained a new neural network and withheld the QuantMig, Swedish, Finnish, and New Zealand (QSFN) flow data. The goal is to understand the dependence of the various regional estimates on Northern migration data. In Fig. S23**C** we plot the distribution over the change in flow estimate relative to the uncertainty,

$$\Delta_{ij} = \left\langle \frac{\left| \hat{F}_{ij}^{\text{original}} - \hat{F}_{ij}^{\text{no QSFN}} \right|}{\text{std}(\hat{F}_{ij})} \right\rangle_t \qquad (7)$$

where $\langle \cdot \rangle_t$ represents the time-average. Dividing by the uncertainty is necessary since we are comparing a single realisation to a distribution. We see that South-South flows are disrupted the least, with a median of shift of 1.4σ; North-South flows and North-North flows are disrupted by 3.4σ and 2.7σ, respectively. The geographic distribution of the change is given in Fig. S23**D**, with the largest deviations occurring across Scandinavia and New Zealand (unsurprisingly), as well as

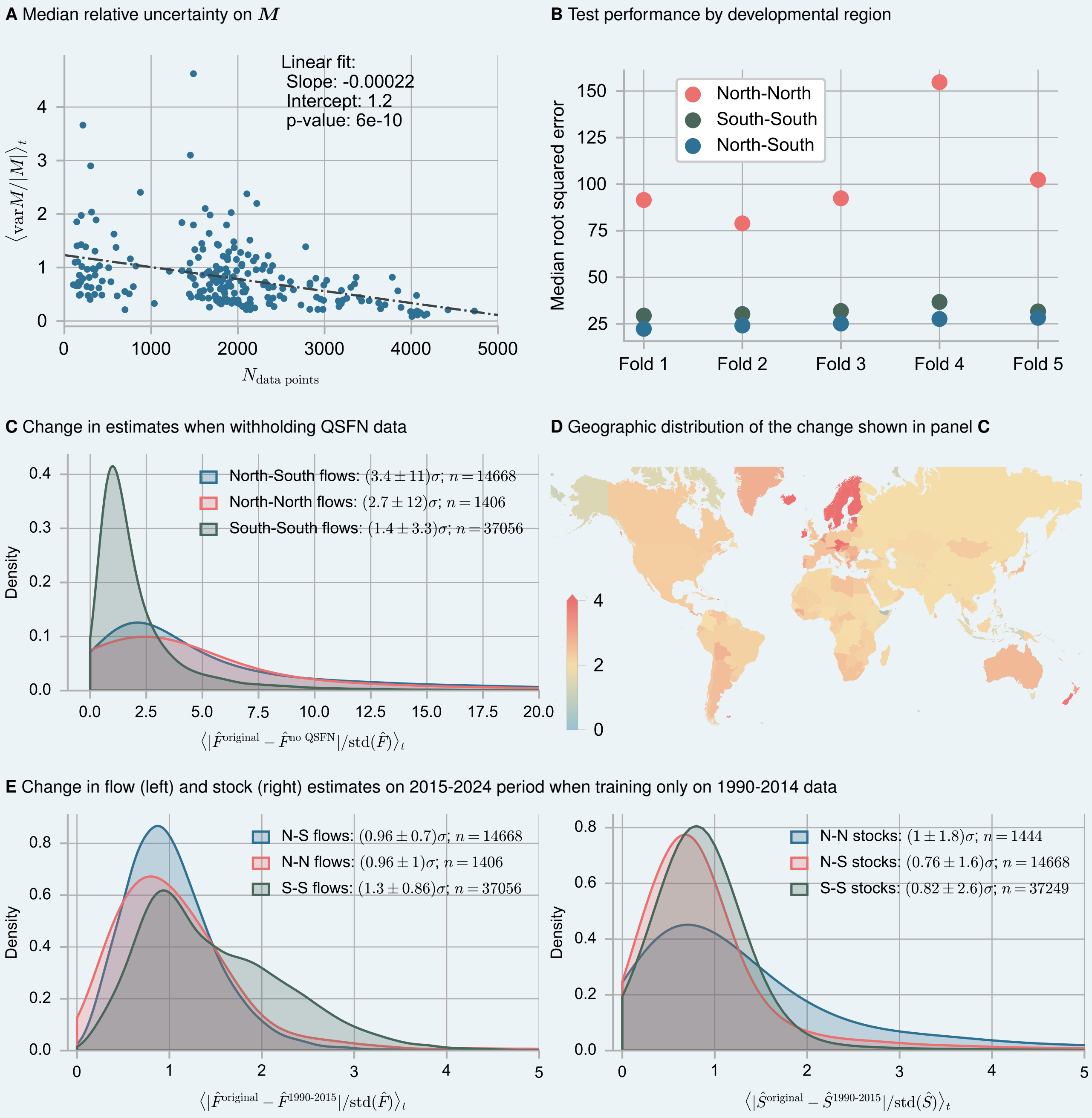

Figure S23: Investigating bias in the estimates with regard to development level. **A** Relative uncertainty on the net migration estimate M (y-axis) as a function of the number of target data points available for each country (stocks, flows, and net migration values). Sweden, Finland, and New Zealand are excluded—they each have around 15,000 data points with a median relative uncertainty of between 10% and 20% (see Fig. 5**C**, main manuscript). Also shown is a linear fit and its parameters. **B** Median root squared error on flow corridors for all five test folds, by developmental region. **C** The change in estimates when withholding QSFN data. Shown are densities over the median absolute difference for each corridor (median taken over time), in units of the standard deviation. **D** The same figures as in panel **C**, this time additionally taking the median over both the destination and origin country and averaging to obtain a single value for each country. **E** The same figures as in panel **C**, this time when training without data from 2015-2024. Shown are the densities on both the flow estimates (left) and the stock estimates (right).

continental Europe. In this panel, we show

$$\frac{1}{2}\left[\text{Median}_i(\Delta_{ij}) + \text{Median}_j(\Delta_{ij})\right], \tag{8}$$

though showing mean values for just the origin or destination yield much the same plot.

Lastly, we also trained a new neural network only on data from 1990–2015, withholding the entire period from 2015 as a validation period. This is to to understand whether the data produces any noticeable differences in forecasting ability across regions. This experiment means withholding the Facebook data, causing the target flows to be made up entirely of North-South and North-North flows. The results are given in Figures S23**E**: we again show the change Eq. (7) to the flow and the stock estimates for the period 2015–2024. Note that this period contains the Covid drop, but we found that restricting ourselves to only using the years 2015–2020 as a validation period did not change the result. The plots indicate that the network's forecasting ability is roughly the same across developmental regions—slightly worse for the global North than the global South for the stocks, slightly better for the flows.

References

1. Australian Bureau of Statistics. *Overseas Migration* https://www.abs.gov.au/statistics/people/population/overseas-migration/latest-release#data-downloads. Accessed: 4 February 2025. Dec. 2024.
2. Statistik Austria. *Wanderungen mit dem Ausland* http://statcube.at/statcube/opendatabase?id=debevwan010. Accessed: 2 March 2025. May 2024.
3. StatBEL. *Migration* https://statbel.fgov.be/en/themes/population/population-movement/migration. Accessed: 2 March 2025. June 2024.
4. Republic of Bulgaria National Statistical Institute. *International migration by age and sex* https://www.nsi.bg/en/content/3072/international-migration-age-and-sex. Accessed: 13 March 2025. Apr. 2024.
5. Statistics Canada. *Estimates of the components of demographic growth, annual* https://www150.statcan.gc.ca/t1/tbl1/en/tv.action?pid=1710000801. Accessed: 19 November 2024. Sept. 2024. https://www150.statcan.gc.ca/t1/tbl1/en/tv.action?pid=1710000801.
6. Czech Statistical Office. *Population and population change of the Czech Lands (annual data)* https://vdb.czso.cz/vdbvo2/faces/en/index.jsf?page=statistiky#katalog=33157. Accessed: 13 March 2025. Feb. 2025.
7. Statistics Denmark. *Immigration and emigration* https://www.dst.dk/en/Statistik/emner/borgere/flytninger/ind-og-udvandring. Accessed: 2 March 2025. Feb. 2025.
8. Statistics Estonia. *RVR03: Migration by sex, age group and type of migration* https://andmed.stat.ee/en/stat/rahvastik__rahvastikusundmused__ranne/RVR03. May 2024.
9. Statistics Finland. *11ab–Immigration and emigration by country of departure or arrival, origin and region, 1990–2023* https://pxdata.stat.fi/PxWeb/pxweb/en/StatFin/StatFin__muutl/statfin_muutl_pxt_11ab.px/. Accessed: 16 February 2025. Dec. 2024.
10. Institut national de la statistique et des études économiques (Insee). *Composantes de la croissance démographique* https://www.insee.fr/fr/statistiques/2381468. Accessed: 23 November 2024. 2025.
11. Statistisches Bundesamt (Destatis). *Wanderungen zwischen Deutschland und dem Ausland (Jahr)* https://www.destatis.de/DE/Themen/Gesellschaft-Umwelt/Bevoelkerung/Wanderungen/Tabellen/wanderungen-zwischen-deutschland-und-dem-ausland-jahr-02.html. Accessed: 19 November 2024. June 2024.

12. Statistics Iceland. *External migration by sex and citizenship 1961-2023* https://px.hagstofa.is/pxen/pxweb/en/Ibuar/Ibuar__buferlaflutningar__buferlaflmillilanda/MAN01400.px/. Accessed: 2 March 2025. July 2024.

13. Central Statistics Office Ireland. *PEA03 - Estimated Migration (Persons in April)* https://data.cso.ie. Accessed: 2 March 2025. Aug. 2024.

14. Istat. *Resident population by sex, live births, deaths, natural increasing, net migration, total balance and birth rates, mortality rate, natural growth rate and total migration rate - Years 1862-2014 at current borders* https://seriestoriche.istat.it/fileadmin/documenti/Table_2.3.xls.

15. Istat. *Migrazioni interne e internazionali della popolazione residente anni 2022-2023* https://www.istat.it/it/files/2024/05/Migrazioni-interne-e-internazionali-della-popolazione-residente.pdf. May 2024.

16. Japan Immigration Services Agency. *Immigration control statistical table: Number of people entering and leaving Japan, Annual Report* https://www.moj.go.jp/isa/policies/statistics/toukei_ichiran_nyukan.html?hl=en. Accessed: 10 March 2025. July 2024.

17. Offical Statistics Portal Latvia. *Long-term international migration by country group 1990–2023* https://data.stat.gov.lv/pxweb/en/OSP_PUB/START__POP__IB__IBE/?tablelist=true. Accessed: 13 March 2025. Mar. 2025.

18. Official Statistics Portal Lithuania. *International migration flows* https://osp.stat.gov.lt/en_GB/gyventoju-migracija. Accessed: 13 March 2025. Jan. 2025.

19. Statistics Netherlands. *How many people immigrate to the Netherlands?* Accessed: 2 March 2025. Dec. 2024.

20. Stats NZ. *International Migration: September 2024* https://www.stats.govt.nz/information-releases/international-migration-september-2024/#annual. Accessed: 19 November 2024. Nov. 2024.

21. Statistics Norway. *05426: Immigration, emigration and net immigration* https://www.ssb.no/en/statbank/table/05426/. Accessed: 11 March 2025. Mar. 2024.

22. Statistics Portugal. *Net migration by place of residence, annual* https://www.ine.pt/xportal/xmain?xpid=INE&xpgid=ine_base_dados. Accessed: 13 March 2025. June 2024.

23. Republic of Slovenia Statistical Office. *International migration by sex, Slovenia, annually* https://pxweb.stat.si/SiStatData/pxweb/en/Data/Data/05N1002S.px/. Accessed: 13 March 2025. Feb. 2024.

24. Statistics Korea. *International Migration Statistics* https://kostat.go.kr/board.es?mid=a20108050000&bid=11745&act=view&list_no=431900. Accessed: 3 March 2025. July 2024.

25. Instituto Nacional de Estadística (INE). *INEbase: International Migratory Balance* https://www.ine.es/dyngs/INEbase/en/operacion.htm?c=Estadistica_C&cid=1254736177000&menu=ultiDatos&idp=1254735573002. Accessed: 4 February 2025. 2023.

26. Statistics Sweden. *Immigrations and emigrations by country of emi-/immigration, region of birth, age and sex.* https://www.statistikdatabasen.scb.se/pxweb/en/ssd/START__BE__BE0101__BE0101J/ImmiEmiFlyttN/. Accessed: 19 November 2024. 2024.

27. Bundesamt für Statistik. *Internationale Wanderungen der ständigen Wohnbevölkerung nach Staatsangehörigkeit, Geschlecht und Alter, 1991-2023* https://www.bfs.admin.ch/bfs/de/home/statistiken/bevoelkerung/migration-integration/internationale-wanderung.assetdetail.32229097.html. Accessed: 2 March 2025. Aug. 2024.

28. National Statistics R.O.C. (Taiwan). *Population and Housing: Number and rates of births, deaths, immigrants and emigrants, marriages and divorces* https://eng.stat.gov.tw/News_Content.aspx?n=4302&s=232173. Accessed: 4 February 2025. 2023.

29. Office for National Statistics. *Long-term international immigration, emigration and net migration flows (provisional)* https://www.ons.gov.uk/peoplepopulationandcommunity/populationandmigration/internationalmigration/datasets/longterminternationalimmigrationemigrationandnetmigrationflowsprovisional. Accessed: 19 November 2024. 2023.
30. Frey, W. H. *Immigration is Driving the Nation's Modest Post-pandemic Population Growth, New Census Data Shows* https://www.brookings.edu/articles/immigration-is-driving-the-nations-modest-post-pandemic-population-growth-new-census-data-shows/. Accessed: 17 February 2025. 2024.
31. U.S. Census Bureau. *International Migration in Population Estimates* Random Samplings blog post. U.S. Department of Commerce, Dec. 2024. https://www.census.gov/newsroom/blogs/random-samplings/2024/12/international-migration-population-estimates.html (2024).
32. Congressional Budget Office. *The Demographic Outlook: 2023 to 2053* tech. rep. 59683 (U.S. Congress, Jan. 2023). https://www.cbo.gov/publication/59683 (2024).
33. United Nations Department of Economic and Social Affairs, Population Division. *World Population Prospects 2024* https://population.un.org/wpp/. New York, 2024.
34. The World Bank. *World Bank Open Data: GDP growth (annual %)* https://data.worldbank.org/indicator/NY.GDP.MKTP.KD.ZG. 2023. https://data.worldbank.org/indicator/NY.GDP.MKTP.KD.ZG.
35. The World Bank. *World Bank Open Data: GDP per capita (constant 2015 US$)* https://data.worldbank.org/indicator/NY.GDP.PCAP.KD. Accessed: 6 February 2025. 2023.
36. United Nations Conference on Trade and Development (UNCTAD). *Gross domestic product: Total and per capita, current and constant (2015) prices, annual* https://unctadstat.unctad.org/datacentre/dataviewer/US.GDPTotal. Accessed: 7 February 2025. 2023.
37. Maddison Project. *Maddison Project Database 2023* https://www.rug.nl/ggdc/historicaldevelopment/maddison/releases/maddison-project-database-2023. Accessed: 6 February 2025. 2023.
38. International Monetary Fund (IMF). *World Economic Outlook Database, October 2024* https://www.imf.org/en/Publications/WEO/weo-database/2024/October. Accessed: 6 February 2025. 2024.
39. Statistics Netherlands (CBS). *Caribbean Netherlands; gross domestic product (GDP) per capita* https://www.cbs.nl/en-gb/figures/detail/85251ENG. Accessed: 7 February 2025. 2023. https://www.cbs.nl/en-gb/figures/detail/85251ENG.
40. United Nations. *National Accounts Main Aggregates Database* https://data.un.org/Data.aspx?d=SNAAMA&f=grID:101;currID:USD;pcFlag:1&c=2,3,5,6&s=_crEngNameOrderBy:asc,yr:desc&v=1. Accessed: 7 February 2025. 2023.
41. Statistia. *GDP of French Overseas Regions* https://www.statista.com/statistics/1075036/gdp-french-overseas-regions/. Accessed: 4 February 2025. 2024.
42. Pacific Data Hub. *National Accounts Dataset* https://sdd.spc.int/dataset/df_national_accounts. Accessed: 8 February 2025. 2023.
43. Pacific Data Hub. *Inflation rates* https://sdd.spc.int/dataset/df_cpi. Accessed: 8 February 2025. 2023.
44. Senate Foreign Affairs, Defence and Trade References Committee. in *East Timor* Accessed: 8 February 2025 (Commonwealth of Australia, 1999).
45. Moody's Ratings. *Credit Opinion: Government of Isle of Man* https://www.gov.im/media/1386794/moodys-isle-of-man-credit-rating-report-december-2024.pdf. Accessed: 10 Feburary 2025. Dec. 2024.

46. HM Government of Gibraltar. *National Income, 2009/10–2023/2024* https://www.gibraltar.gov.gi/uploads/statistics/2024/National%20Income/2024.01.02%20Revised%202024.07.01%20National%20Income%20for%20Website%20BB24.png. Accessed: 10 Feburary 2025. Jan. 2024.

47. Falklands Directorate of Policy, Economy & Corporate Services. *National Accounts* https://www.falklands.gov.fk/policy/statistics/national-accounts. Accessed: 10 Feburary 2025. Nov. 2024.

48. Falklands Jane Cameron National Archives. *Economy of the Falkland Islands* https://www.nationalarchives.gov.fk/jdownloads/Trade%20and%20Industry/R-TRA-ECO-2-2.%20Economy%20of%20the%20Falkland%20Islands.pdf. Accessed: 11 February 2025. 1989.

49. Gaulier, G. & Zignago, S. *BACI: International Trade Database at the Product-Level. The 1994-2007 Version* Working Papers 2010-23 (CEPII, Oct. 2010). https://www.cepii.fr/CEPII/fr/publications/wp/abstract.asp?NoDoc=2726.

50. Centre d'Études Prospectives et d'Informations Internationales (CEPII). *BACI Dataset* https://www.cepii.fr/CEPII/en/bdd_modele/bdd_modele_item.asp?id=37. Accessed: 26 March 2025. Jan. 2025.

51. Conte, M., Cotterlaz, P. & Mayer, T. *The CEPII Gravity Database* Working Papers 2022-05 (CEPII research center, July 2022). https://www.cepii.fr/CEPII/en/bdd_modele/bdd_modele_item.asp?id=8.

52. Gurevich, T., Herman, P. R., Toubal, F. & Yotov, Y. V. *The Domestic and International Common Language (DICL) Database* USITC Economics Working Paper 2024–03–A (U.S. International Trade Commission, 2024).

53. Maoz, Z. & Henderson, E. A. The World Religion Dataset, 1945–2010: Logic, Estimates, and Trends. *International Interactions* **39,** 265–291 (2013).

54. Central Intelligence Agency. *The World Factbook 2024* (Central Intelligence Agency, Washington, DC, 2024).

55. Barron, J. T. *Continuously Differentiable Exponential Linear Units* 2017. arXiv: 1704.07483 [cs.LG]. https://arxiv.org/abs/1704.07483.
