# Supplementary material for: Deep learning four decades of human migration
Source: Nature. 2026 Jun 10;655(8121):148–57. doi: 10.1038/s41586-026-10611-7 (PMC13322962; doi:10.1038/s41586-026-10611-7)
Supplement: Supplementary file 3 — Peer Review File [file 41586_2026_10611_MOESM3_ESM.pdf]

nature portfolio

Peer Review File

Deep learning four decades of human migration

Corresponding Author: Dr Thomas Gaskin

Any redactions in this file are there to maintain patient confidentiality, the confidentiality of unpublished data, or to remove third-party material.

This file contains all reviewer reports in order by version, followed by all author rebuttals in order by version.

Version 0:

Reviewer comments:

Referee #1

(Remarks to the Author)
This article advances a new approach to estimating annual global migration stocks and flows across 230 countries from 1990 to the present. This approach relies on estimates obtained from training of a "deep recurrent neural network" that examines flow patterns based on a set of "target data, comprising the UN DESA migrant stocks, Facebook data, as well as a small number of predominantly European bilateral flows and net migration data."

This proposed methodology makes the following contributions. First, it addresses shortcomings in data availability in the country of destination and differentiates flows by country of birth and country of origin. This approach is most helpful for countries in the Global South, where the data is not as comprehensive as in the Global North. Second, this approach triangulates across different data sources, incorporating 18 covariates across all countries that capture geographic, economic, cultural, social, and political information.

That said, we have a few reservations.

While the approach is novel, it raises several questions about the method, the data, the accuracy of prediction, and other aspects that limit its potential contributions in its current form. First, how do current data limitations shape these estimates? The bulk of the learning is occurring using data from a small subset of locations, primarily in Europe and the Global North, which is more reliable than in other areas. This feature generates more uncertainty about the migration estimates in regions of the Global South. That is not a weakness of the proposed algorithm, given that the data estimates the authors compare have high uncertainty. Our concern is about how this algorithm performs when it is asked to be pushed further into the future: Does training these models using data of the highest quality in one part of the world create path dependencies later on that will be misaligned with reality?

Moreover, since this is a new method, can the authors test the accuracy of the predictions using a simulation? Given the aforementioned problem, it would be beneficial to understand how sensitive the algorithm is to this issue of missing/noisy/low-quality data. It would not be impossible to generate world-year estimates and remove (or muss up) observations in ways that reflect our current state of data collection.

We recognize that this is a significant amount of work, but since this is a new tool, we want to understand the scope conditions under which it is likely to provide reliable estimates and under what data circumstances it yields totally inadequate estimates. Knowing the promise and limits of the tool will make us more confident about its application. As is, we are less confident about these findings based on the authors' approach of benchmarking against existing data sources alone.

We appreciate the modeling of confidence bounds and the discussion on meaningful uncertainty quantification, but given the global scope of this paper. There is significant heterogeneity within and across regions (i.e., continents, sub-regions, etc.) that needs more scrutiny and discussion. We don't get a clear sense of how this uncertainty might vary across countries and regions, etc., so there is no clear takeaway from this approach.

A few other questions and observations (these are minor compared to the above concerns).

Figure 1: While it illustrates the challenges of measuring migration, it is unclear how widespread these challenges are, where the challenges are most severe, etc. In other words, it raises a concern without documenting the extent and nature of this concern more carefully.

Figures 3-5: The countries and regions featured in these figures appear to have been selected ad hoc. There is no clear rationale as to why certain countries or regions are highlighted and not others. As a result, the findings are illustrative but not conclusive, ultimately undermining the paper's contributions.

On an editorial note, we need better legends, consistency in axis scales, and clear labels in the figures to enable readers to understand these findings.

The paper relies heavily on published papers by one of the authors to establish key data and methodological details. These citations are included in the references, but a full understanding of many key issues requires reading the referenced papers as well. This over-reliance on prior publications places undue burdens on the reviewers. Since the paper is highly technical, more details must be provided in full in this manuscript to help reviewers understand this approach and to assess its merits, especially those who are not familiar with this methodological approach (and its potential limitations).

(Remarks on code availability)
We could not replicate the analysis.

The problem seems to be missing data very early in the code. You can see the path errors in Data/__init__.py. For example, "world_shapefile/ne_50m_admin_0_countries.shp" is missing, and the entire Flow_data folder ("Flow_data\QuantMig_data\Quantmig_flows.nc.") could not be found.

To our knowledge, these files are not available on GitHub or Zenodo. It is possible that these files were available on another path or generated from data available in these sources, but no explanation was provided for how to generate them.

Referee #2

(Remarks to the Author)
I co-reviewed this manuscript with one of the reviewers who provided the listed reports.

(Remarks on code availability)

Referee #3

(Remarks to the Author)
General Summary

This is an excellent and timely paper, significantly adding to the literature on the estimation of global migration flows by applying state-of-the-art analytical methods (deep neural networks) to the existing migrant stock, flow and covariate data, with the additional advantage of assessing the uncertainty of the resulting estimates. By providing a much-needed update on the global picture of migration flows, the paper and the results presented therein will be of interest to general scientific readership and are thus appropriate for publication in Nature.

A Summary of the key results

The paper provides a set of annual migration flow and migrant stock estimates between 230 countries and regions of the world, broken down by country of birth. The estimates are obtained through an application of a novel methodology, based on deep neural networks, trained on 18 migration covariates of different types. The estimates are accompanied with propagated uncertainty, which is (rightly) framed as a way to indicate the need for future data collection.

B Originality and significance

The results provide a significant advancement of the state of the art, where most of the existing estimates are either based on five-year transitions (previous work by Guy Abel), are not broken down by the country of birth -- itself a variable strongly associated with the choice of subsequent destinations -- or are confined to subsets of countries with relatively good data availability (IMEM, QuantMig, Human Migration Database).

C Data & methodology

The paper is based on a broad array of data on migration flows, stocks and covariates, all of which come from well-established sources and are well justified, both in the main text and the methods section, as well as in the Supporting Information. The choice of a method (deep neural network) in this context is innovative and helps illustrate the potential of using deep learning algorithms for estimating migration - or in fact other flow variables in a closed system.

D Appropriate use of statistics and treatment of uncertainties

The statistical - and more broadly, machine learning methods are fully appropriate for the task, and judging by the results, the analysis has been carried out correctly. The uncertainty of the estimates has been explicitly acknowledged: my only suggestion here is to add detail to the discussion of uncertainty quantification in the Methods section (see F below).

E Conclusions

The conclusions are sound, well justified on the grounds of the analysis that has been carried out, and seem robust. The article provides a new dataset of migration estimates, obtained by using innovative methods, and making a few important contributions to the literature. A few minor suggestions for adding nuance to the interpretations are listed below.

F Suggested improvements

Overall, the manuscript has been a real pleasure to review. I only have a few minor suggestions to make it more accessible and to make the discussion more robust (and on occasion more subtle).

- Main text, p2, left column: The United Kingdom has been phasing out reliance on passenger surveys - better rephrase as "rely on visa records, administrative data, and until recently passenger surveys." In addition, some countries use surveys (such as the Labour Force Survey).

- Main text, p3, left column: When you introduce the loss function, it would help defining it in intuitive terms, to aid the more generalist readers of the paper, as a function that gives weight to the discrepancy between the model-based estimates and the data, or something similar.

- Main text, p4, left column: The argument about long memory can be seen as controversial and merits a more thorough discussion. In the migration forecasting literature (Bijak, Wiśniowski), one of the common findings is non-stationarity, which implies short memory, and ever-changing equilibria. To that end, using a whole 40-year training period may seem counterproductive. In this context, to make the argument more nuanced and also consistent with what is known about migration processes, it is worth decoupling the lack of stability (non-stationarity) of migration processes over time from some persistence of patterns across space (gravity). One lens to look at that could be offered by the generation-distribution framework [Willekens F (1994) Monitoring International Migration Flows in Europe, Eur J Pop, 10(1): 1–42]: you are concerned with the distribution aspect here.

- Main text, p4, right column: Do the UNHCR asylum data that you use include temporary protection? If not, or not precisely, this may explain why you do not pick up the Ukraine to Poland flow in 2022 as the largest one (1.568 million temporary protection decisions granted between 4 March and 31 December 2022 as per Eurostat: https://ec.europa.eu/eurostat/statistics-explained/index.php?title=Temporary_protection_for_persons_fleeing_Ukraine_-_monthly_statistics). I suggest rewriting this fragment to avoid suggesting that the main outflow from Ukraine was to Russia, which it was not: here, context is everything.

- Main text, p6, Figure 3: The alignment of your estimates with the QuantMig ones in the presented examples is remarkable. Are there any flows for which the alignment is much weaker, and are there any lessons (with respect to data availability or features of countries) that can be drawn from that?

- Main text, p10, left column: Given the importance of life expectancy and GDP, have you considered using the Human Development Index instead, for the sake of slightly greater parsimony?

- Main text, p10, right column: You make an interesting point about possible applications to forecasting, although I am not convinced that the past data are sufficiently informative to improve the inferences here: there are problems with predicting covariates, the stability of their (uncertain) relationships, and so on, not to mention the philosophical question of memory mentioned above. I think this merits a more nuanced discussion, which can be centred around the purpose of your model, which is estimation, as opposed to forecasting or explanation. This is where deep neural networks can really shine, and as a byproduct, you can argue that you offer a coherent and improved knowledge base for other types of studies.

- Methods, p14: It is worth adding that equation (2) is a deterministic identity.

- Methods, p15, Figure 7: At the end of the caption, it is worth adding that (as I presume) the thicker the lines the higher the weights, to bring home the weight-uncertainty relationship.

- Methods, p17/18: The targetting of stock differences rather than stocks themselves is novel - I would highlight this as an additional contribution vis a vis the state of the art.

- Methods, p18: The CeLU function is missing a definition on the left-hand side and in the text - readers unfamiliar with neural networks will not know which function it is and what it does (ie. activate a layer).

- Methods, p18: The section on uncertainty quantification is a bit underdeveloped and is worth expanding. You mention model uncertainty and covariate uncertainty as the two key sources, but there is vast literature (including IMEM/QuantMig) on the role of measurement errors, definitional mismatches, and so on. You can of course define your two key sources (model and covariates) in such a way that they encompass all the other ones, but this needs to be made explicit. Similarly,

given that your approach to UQ is rather simple, I would expect a longer critical discussion as to which aspects of uncertainty are included, which are not, and why what you propose is a "good enough" approximation of the total uncertainty (there is a similar argument in the QuantMig book - see G below).

- Methods, p19: In (11), better denote the offset by a different letter to avoid any subconscious confusion with births or birth rates amongst demographically-minded readers.

G References and credit to previous work

The paper is very well grounded in the existing body of work on estimating migration flows. For completeness, you may also consider referring to the IMEM model [Raymer J et al. (2013) Integrated Modeling of European Migration. JASA, 108: 801--819], which was a direct predecessor to the QuantMig work, and its follow up in the Human Migration Database [Dańko M (2024) HMigD I App: The Human Migration Database I App. Version 3.3.1. Rostock: Max Planck Institute for Demographic Research. https://maciej-jan-danko.shinyapps.io/HMigD_Shiny_App_I]. For the QuantMig references themseleves [8,9], the corresponding published work is Chapter 5 (Aristotelous et al.) in: Bijak J (ed.) (2024) From Uncertainty to Policy: A Guide to Migration Scenarios. Cheltenham: Edward Elgar. https://doi.org/10.4337/9781035319800. The QuantMig book also includes arguments on non-stationarity, building on earlier work, especially Bijak J (2010) Forecasting International Migration in Europe: A Bayesian View. Springer.

H Clarity and context

The paper is clearly written and is very well situated within the broader context of the work on (global) estimation of migration flows. The maps, graphs and tables are very aesthetically laid out: in particular, I found the conceptual diagram (Fig 7) very clear and informative. A few minor editorial suggestions for making the text even more accessible to generalist readership of Nature are listed under F above.

I Supporting Information (SI)

Supporting Information is well prepared and adds value to the manuscript, by presenting methodological detail and a selection of specific results. The data manipulations and the associated assumptions made are well documented and justified, and the validation experiments using synthetic data and unseen data are informative. The choice of the datasets or covariates is clear and follows the state of the art. With respect to the SI, I only have a few minor comments for greater clarity:

- On p6, just under Figure S3, it is not clear where the fist (unnumbered) formula comes from: adding a sentence or two with an explanation would be very helpful.

- On p7, before equation (2), it would help the reader either to repeat the formula for the loss function from the main manuscript, or at least provide a cross-reference.

- On pp7/8, it looks like a lot of the information on the errors is borrowed from the QuantMig estimates for a subset of flows: if so, this is a strong assumption, which merits a critical comment.

- Throughout, I would explain acronyms, especially the more technical ones, on their first occurrence (e.g. CeLU, ReLU on p15 -- the former also in Methods, p18). Some other terms (Yeo-Johnson transform) can be cross-referenced to the Methods section.

- In the main manuscript, "SI" needs expanding on the first occurrence as well (p2)

(Remarks on code availability)
The checklists are very clear and diligently prepared. There are no ethical issues and all statistical reporting is state of the art. I have sampled content, including the code, from the GitHub repository and found it very well prepared and documented - the deposit itself is in many ways exemplary. My only suggestion here (for later - with no bearing on the publication of the paper) to ensure preservation would be to duplicate the current version of the code on Zenodo, as a version of record, which would be independent from a private GitHub account.

Referee #4

(Remarks to the Author)
**Summary of the key results

This paper presents a global dataset and framework for modeling international migration flows using neural networks, trained on 18 features crossing political, geographic, and socioeconomic characteristics. Along with the model, the authors released the outputs as a new dataset that could be used by others in future studies, representing the annual migration flows between 230 countries from 1990 to 2023, broken down by the country of birth and uncertainty quantifications of those estimates. Within their manuscript, they highlight particular case studies of migration flows within Europe and Africa, as well as some country-specific flows.

The reviewers, though, highlight below the lack of sufficient methodological comparison to other machine learning based approaches to human migration modeling to establish that the presented approach and result are more accurate than what other approaches can bring using the same global migration dataset of variables and historic flows, contributed by this paper.

** Originality and significance: if not novel, please include a reference

This work argues novelty in the use of recurrent neural networks to achieve annual migration estimates at such a granular level, while taking into account prior information. The authors' methods increase both the spatial and temporal resolution of existing migration estimates used by major organizations, and predictions are also delimited by country of birth. Authors argue that migration estimates in areas that are data-deficient are improved as the uncertainty in those regions is decreased compared to QuantMig, the most recent development in migration estimation, according to the authors. Their approach is also readily accessible (and well documented) and can easily be adopted by those interested in modeling migration on a finer geographic scale.

*** Data & methodology: validity of approach, quality of data, quality of presentation

The quality of the visualizations is excellent. However, some figures are challenging to digest due to unclear captions, labels, or references.

- For example, Figure 1's title could replace Registration-based measurements to specify that it is specifically for flows from Poland to Germany.
- This also affects Figure 1B.
- Specifying the unit of measurement on the axes for Figure 2B/C (assuming this is millions of people).
- it is unclear what the size of each region's band means in Figure 2b. Is this the estimated population in each region and the arrows represent the flow?
- We suggest updating all written references in the manuscript to specify the figure number along with the panel (i.e., instead of "as seen in Panel B", specify "as seen in FigX.B").
- Figure 6 was challenging to understand. The titles in Panel A should specify the data source (assuming DESA?) and a color legend should be included to understand the gradient from green -> yellow -> red.

The underlying data used matches the standard of quality of what other migration estimation studies use, and comes from diverse locations (Facebook, labor stats, etc.). However, some descriptions of how much each datasource is used when making up the target data would be useful.

** Appropriate use of statistics and treatment of uncertainties

Statistics appear to be used appropriately, and estimates are reported with confidence bounds.

** Conclusions: robustness, validity, reliability

Overall, conclusions on the migration modeling could be reliable, as authors either highlight how close the estimates are to the data source or the QuantMig estimations, or they discuss a few examples of where the QuantMig estimates and their estimates differ with some justifications. However, these comparisons were not presented on a global scale (or all the countries estimated in QuantMig).
The discussion on the important features based on their model is great, but it would also be interesting to know which features were the -least- important for the knowledge insight. Some discussion of this was included in the elasticity section of the methods, but including a brief statement in the main conclusions would be useful.

** Suggested improvements: experiments, data for possible revision

There are several technical questions/suggestions:

Why was cross validation not used to evaluate over all flow corridors? How did you determine what flow corridors to train vs test? Were they spatially stratified were they temporarily stratified? There is a vast literature on the undesirable sensitivity to a single train / test split as was done here. Given the possible strong geographic and temporal differences the choice of train and test has to be done very carefully and given that it is hard ot get right, it needs to be repeated (randomized) multiple times for the final reported metrics of performance of the model to be representative.

- Ideally the authors should report metrics over repeated train/test splits or just do 5-fold cross validation. If not, the authors should add a strong justification for why cross validation or repeated train/test splitting was not utilized with more details on the representation in the training/test data (making sure the test set was representative).

It would be useful to move the justification for why correlation/MRE were used to the start of the Testing and Validation section, as it wasn't justified until after some results were already discussed and in the supplementary information.

The choice of which years/regions to highlight in Figures 3A and 4A should be justified.

The authors should comment on whether the migration insights derived from your model that are mapped in these plots can

be unveiled through the existing approaches. What is the new knowledge your method unveils here (that with the raw training data or with prior approaches would either be unavailable or the models would have pointed to a different insight about migration)?
Similarly, on page 8 before the Testing and Validation section, we believe adding an argument for how the uncertainties derived from the QuantMig study differ from the estimates derived for the same countries in the proposed approach. Specifically detailing new conclusions and compare/contrasting the knowledge insights between the two methods.

** References: appropriate credit to previous work?

The strongest weakness of the study is the contradiction with the authors' claiming innovation on bringing a sophisticated machine learning (ML) approach (recurrent neural networks) to the problem of predicting migrations vs. not actually including a deeper literature review on ML-based approaches to human migration and choosing to benchmark only against a single paper / approach QuantMig.
The QuantMig method, which the authors use as their baseline, does not cite existing technical approaches for this predictive problem or have a proper literature review and hence it is difficult to understand why QuantMig is the best baseline for the approach presented here. Therefore, this submitted manuscript is missing a proper literature review, especially as it relates to proposing a machine learning model (recurrent Neural Networks) as the main method's contribution, and hence, prior ML approaches to migration prediction should be addressed. There is a brief mention of "gravity models", but no elaboration beyond a citation to a single **discussion** paper. There are many studies that build statistical / ML models to estimate migration, some purely for migration research, others in the context of studying migration-based questions, such as forced migration under, for example, sea level rise under climate change, and those (often machine learning) models apply to the general migration estimation problem. Here are several papers that all proposed ML-based approaches relevant to modeling human migrations and the broader topic of human origin-destination flows modeling, any of which would be just as valid if not better baseline for the presented approach than QuantMig (many of these approaches can provide uncertainty estimates in one for or another, either directly through the model or from bootstrapping train/test splits etc)

- Hauer, M. Migration induced by sea-level rise could reshape the US population landscape. Nature Clim Change 7, 321–325 (2017). https://doi.org/10.1038/nclimate3271,
- Robinson C, Dilkina B, Moreno-Cruz J (2020) Modeling migration patterns in the USA under sea level rise. PLoS ONE 15(1): e0227436. https://doi.org/10.1371/journal.pone.0227436
- Simini, F., Barlacchi, G., Luca, M. et al. A Deep Gravity model for mobility flows generation. Nat Commun 12, 6576 (2021). https://doi.org/10.1038/s41467-021-26752-4
- Can Rong, Jingtao Ding, and Yong Li. 2024. An Interdisciplinary Survey on Origin-destination Flows Modeling: Theory and Techniques. ACM Comput. Surv. 57, 1, Article 4 (January 2025), 49 pages. https://doi.org/10.1145/3682058

The QuantMig approach benchmarked here was not even re-applied to the full global dataset used in this study, but only the reported estimates for the 30 countries that the QuantMig paper focused on were compared. Hence, from a methodological perspective, there is no apples-to-apples comparison on the same dataset and label (and train/test splits) used for the proposed approach here to other ML-based methods applied to the exact same data. We request that such a methodological benchmark is appropriate to be provided to establish the relative strength of the new method. This does not take away from the contribution of compiling the global migration dataset and variables that the authors provided here, but still leaves open to questions whether the recurrent Neural Network method proposed is significantly better than other ML-based approaches proposed in the past.

** Clarity and context: lucidity of abstract/summary, appropriateness of abstract, introduction and conclusions

The manuscript is well structured, with the conclusions and domain-specific discussion especially clear and contextualized appropriately.

** Detailed Comments
- On page 8, you make a reference to the target data. What is the target data?
- Made a reference to Figure 5D on page 8, but we believe this should be 5C.
- On page 8 before Testing and Validation, we believe adding an argument for how your presentation of the uncertainty

- A color legend or more descriptive caption should be added to Figure 5A and C. We assume it is distance to x=y?
- Figure 6a - Is the first chart DESA Stocks? Is this a training/testing dataset?
- Page 7 appears to be missing a reference to citation 34, when mentioning the WPP.
- We recommend reminding the readers what mu is on page 7, as it is introduced very early on in the paper.
- We recommend adding some sort of indicator on the size of your datasets that you are using.
- In the introduction, it is unclear how the digital trace data from Facebook is calculated and ultimately measures migration. Additionally, what are the biases of using this data? Communicating that briefly, as mentioned with the labour migration and UNDESA data sources, would be a good addition.
- Page 14 says there are 4 sources, but then only lists 3 items in the list.

(Remarks on code availability)

The codebase for this study is fully open source and available on GitHub. The documentation for how to utilize the code in the README file is thorough and easy to follow. Links to the dataset are included, enabling complete reproduction of the results of the paper.

Referee #5

(Remarks to the Author)
I co-reviewed this manuscript with one of the reviewers who provided the listed reports.

(Remarks on code availability)

Version 1:

Reviewer comments:

Referee #1

(Remarks to the Author)
We appreciate the authors' concerted efforts to respond to our comments from the previous round. This revision clarifies several key aspects of the paper, including its methodological underpinnings and empirical contributions. It is well-written and more accessible to readers.

Below, we addressed the key questions posed to us as reviewers.

A. Key results. Please summarize what you consider to be the outstanding features of the work.

This article provides estimates of migration flows and stocks for "annual migration flows and stocks between 230 countries and regions, spanning the period from 1990 to the present. Such a dataset, if validated, could be helpful to other researchers interested in this topic.

B. Originality and significance. If the conclusions are not original, please provide relevant references. On a more subjective note, do you feel that the results presented are of immediate interest to many people in your own discipline, or to people from several disciplines?

The article seeks to quantify annual global migration using a recurrent neural network. It builds on prior work by the authors using a similar approach for a more limited set of countries and a shorter time span. This approach is original and improves on prior analytical approaches that rely on administrative and demographic datasets. The dataset will be of interest to researchers from a broad range of disciplines.

However, we are unsure about the originality of the findings. What the paper does well is to illustrate and confirm major findings previously known about specific global migration flows (see pp. 8-10 on testing and validation).

In that sense, the methodological approach is original, but its empirical findings (to the extent that the paper contains any substantive insights) are neither original nor significant. This brings us to a more general point: Is Nature interested in publishing work that highlights a new method approach, or are new empirical findings a core criterion for publication?

Data & methodology. Is the approach valid? Are the data and presentation of good quality? Please note that we expect our reviewers to review all data, including the Supplementary Information.

Yes. The approach is valid. The revised draft carefully notes the specificity of datasets and other details. The paper also highlights how this approach extends prior approaches to estimating these migration flows.

Appropriate use of statistics and treatment of uncertainties (if applicable). All error bars should be defined in the corresponding figure legends. Please include in your report a specific comment on the appropriateness of any statistical tests, and the accuracy of the description of any error bars and probability values.

Yes. The authors were careful in describing the limitations and uncertainties of these estimates. Figures are accurate, with labels, titles, legends, etc., a clear improvement over the prior round.

E. Conclusions. Are the conclusions and data interpretation robust, valid, appropriate and reliable?
Does the manuscript have flaws that should prohibit its publication? If so, please provide details.

We note two main concerns.

First, the current draft remains under-theorized and under-motivated. The paper lacks a clear intellectual motivation. Here are a few questions: What is the scale of the "problem" (i.e., inadequate annual data coverage) that motivates this analysis? Does the proposed approach and solution improve on the existing data? An articulation of the scale of the problem and why

it needs to be solved will help readers grasp the significance of this paper and more effectively motivate the entire analysis, beyond just "improving resolution." To use an analogy, bigger maps are not necessarily better tools. The paper might be falling into the "big-data trap," where more data is "assumed" to be always better.

For example, in response to our prior comment, the authors wrote: "The challenges are very real, and we have added some further explanation and references to point readers towards
a more thorough documentation of the problem. Figure 1 is merely intended to illustrate the situation, and it is beyond the scope of our manuscript to conclusively demonstrate that global migration data is inconsistent, difficult to measure reliably, and in need of harmonization." This is precisely the point that we raised: how large is this data coverage problem? And how far does this new approach help improve this situation? What new and original insights do we gain about global migration patterns using this new approach and improved dataset?

Throughout the paper, the authors cite many data points that "confirm" what we already know about various flows, but, to the best of our reading, no new findings emerge from this approach. We need a couple of good examples of where the current data has really failed researchers, such that this sophisticated analysis (set of estimates) is warranted. Perhaps the authors can provide a few examples in which the new dataset fills a previously unknown gap or contradicts widely expected findings. That will help illustrate the value of this approach.

In this version, many of the authors' examples reflect what is widely known. For instance, it is not surprising that one of the most significant migration events in the past 30 years is driven by the conflict in the DRC, nor that the main migration routes are from the US-Mexico border and from Asia to the Middle East. Likewise, we know that migration has been steadily increasing.

As is, the main contribution is a slightly better description. We can see this in the results headers: a global map of migration, model migration in the global south, and revised UN figures.

To be fair, the authors included a short discussion of the potential use cases in the discussion:
"Reliable migration data are of great interest to economists, as covariates in econometric models; to epidemiologists, for understanding the spread of infectious diseases; and to demographers, for projecting population growth." We agree, but this underscores our point above: the paper presents a new dataset and a new method for estimating it, but it has yet to offer any new empirical findings.

We are sympathetic to the authors' space constraints, but the authors need to make a stronger case for the problem and the utility of their method. This is the main contribution, and it is mostly hidden by all the technical discussion (which is, of course, important) that is crowding out the argument. This may mean including a version of Figure 2 in the response memo in the main manuscript.

Let us also be clear. We see the potential of this new data approach, and the manuscript makes a welcoming methodological contribution to the study of global migration flows.

Second, this is a new comment, as we did not raise it in the prior round. How do unauthorized migrants shape these estimates and findings? How does the model account for them? What are the key assumptions made about them? These questions cut to the core of the models and these estimates, including the paper's intellectual motivation (better data, more detail, and better descriptions).

For example, when estimates and counts of the Mexican immigrant inflow in the US exceed the count of Mexican migrant outflow from Mexico each year, both measures could be accurate if the differential/discrepancy is due to undocumented migrants from Mexico to the US. Therefore, the discrepancy in records/sources reflects accurate counting, not errors in the counts/estimates.

There are several scenarios here. Both Mexico and the US undercount the undocumented, given the more clandestine movements of this migration flow. Mexico might accurately count them, but the US undercounts them (because they are unauthorized). Mexico might not keep an official count of this group of migrants, yet their numbers appear in the US Census, which does include a count of the undocumented population. Etc. Each scenario will affect the estimates used as input to the deep learning models. Moreover, the assumptions about any observed discrepancies matter in how we treat these datapoints (as valid or invalid).

On a related note, the authors need to be more precise and draw a distinction between migrant "stocks" and "flows." For example, the abstract mentions both "migration flows and stocks between 230 countries and regions." Does this approach improve estimates for both stocks and flows? Or only flows? These two measures capture different aspects of migration. Our reading is that the paper addresses the "flows," but not the "stocks."

F. Suggested improvements. Please list additional experiments or data that could help strengthen the work in a revision.

N/A. See above comments under "Validity"

G. References. Does the manuscript reference previous literature appropriately?

Yes. Very careful in describing its contributions to prior work.

H. Clarity and context. Is the abstract clear and accessible? Are the abstract and introduction appropriate?

Yes. The paper is clearly written and is accessible. Abstract and introduction are appropriate.

***

Please indicate any particular aspect of the manuscript, data or analyses that you feel is outside the scope of your expertise, or that you were unable to assess fully.

We reviewed the data in Python. Unlike the prior round, the data loaded and looked structurally sound. We did not have time to run the entire code (I think it might take days), but the data is available and matches what the authors described in their documentation.

***

Other minor points

The authors could be more selective about what visualizations are critical to make their points. In this version, we are still presented with lots of information that is not always important or well-explained.

We are not sure what to make of their point in the memo: that predictions fare worse with more data. This raises questions for us about the certainty of their South-South predictions, which are then trained on a single data source. Couldn't these be really off?

We appreciate the authors' validation efforts using simulation. That the authors' estimates are likely to remain consistent even with substantial missing data is a strength of their algorithm.

(Remarks on code availability)
We reviewed the data in Python. Unlike the prior round, the data loaded and looked structurally sound. We did not have time to run the entire code (I think it might take days), but the data is available and matches what the authors described in their documentation.

Referee #2

(Remarks to the Author)
I co-reviewed this manuscript with one of the reviewers who provided the listed reports.

(Remarks on code availability)
I can confirm that I was able to download and run the code.

Referee #3

(Remarks to the Author)
I thank the authors for a very thorough and thoughtful engagement with the reviews, an exemplary rebuttal letter, and in particular for the additional piece of analysis to demonstrate that the model is not very sensitive to removal of some of the the most complete data (Nordic countries & NZ), and still provides robust estimates for flows in the "Global South". I also appreciated the Taylor expansion argument -- spot on.

Just one small editorial point: in the new text, both in the article and SI, the use of upper caps in "QuantMig" needs unifying, but this can be left until the copyediting or proofs stages.

On the whole, it was a pleasure reviewing this paper and engaging in such a high-calibre exchange. I am therefore happy to endorse the current version of the article for publication in Nature.

(Remarks on code availability)
The code was examined before, and the GitHub page is exemplary. Readme is present and includes thorough instructions for running the code. Together with the explanations provided by the Authors in the Rebuttal letter, I think this should be sufficient for any reproducibility/replication needs.

Referee #4

(Remarks to the Author)
We thank the authors for the responses to our questions raised during our initial review and for the edits. We have responded to each of the authors' responses to our originally raised points below.

1. Figure 1A’s title could replace Registration-based measurements to specify that it is specifically for flows from Poland to Germany. This also affects Figure 1B.
• The authors’ edits are satisfactory.

2. Specify the unit of measurement on the axes for Figure 2B/C (assuming this is millions of people)
• The authors’ edits are satisfactory.

3. It is unclear what the size of each region’s band means in Figure 2B. Is this the estimated population in each region and the arrows represent the flow?
• The authors have added important context to the figure caption to improve the interpretability of this figure. However, we still want to confirm, the overall size of each region’s band (not the base of the arrow, but the dark colored rounded sections on the outside) do not represent something significant like the population of the region? If it does not represent anything of significance, the edits are satisfactory. If it does represent something, this could still be further clarified in the figure caption.

4. We suggest updating all written references in the manuscript to specify the figure number along with the panel (i.e.,instead of “as seen in Panel B”, specify “as seen in Fig X.B”).
• There is one instance where the panel language remains at the bottom of the left column on page 5, but it is made clearer by adding ‘3B’. To maintain consistency, this could be changed to ‘Figure 3B’, but we don’t believe this stylistic choice should affect acceptance.

5. Figure 6 was challenging to understand. The titles in Panel A should specify the data source (assuming DESA?) and a color legend should be included to understand the gradient from green -> yellow -> red.
• The authors' edits on the legend are satisfactory. We believe including the datasource in the title would still make the text more readable to an interdisciplinary audience, but we do not believe this stylistic choice should affect acceptance.

6. The underlying data used matches the standard of quality of what other migration estimation studies use, and comes from diverse locations (Facebook, labor stats, etc.). However, some descriptions of how much each data source is used when making up the target data would be useful.
• The authors’ edits are satisfactory.

7. Overall, conclusions on the migration modeling could be reliable, as authors either highlight how close the estimates are to the data source or the QuantMig estimations, or they discuss a few examples of where the QuantMig estimates and their estimates differ with some justifications. However, these comparisons were not presented on a global scale (or all the countries estimated in QuantMig). The discussion on the important features based on their model is great, but it would also be interesting to know which features were the -least- important for the knowledge insight. Some discussion of this was included in the elasticity section of the methods, but including a brief statement in the main conclusions would be useful.
• The authors’ edits are satisfactory.

8. Why was cross validation not used to evaluate over all flow corridors? How did you determine what flow corridors to train vs test? Were they spatially stratified were they temporarily stratified? There is a vast literature on the undesirable sensitivity to a single train / test split as was done here. Given the possible strong geographic and temporal differences the choice of train and test has to be done very carefully and given that it is hard to get right, it needs to be repeated (randomized) multiple times for the final reported metrics of performance of the model to be representative. Ideally the authors should report metrics over repeated train/test splits or just do 5-fold cross validation. If not, the authors should add a strong justification for why cross validation or repeated train/test splitting was not utilized with more details on the representation in the training/test data (making sure the test set was representative).
• The authors’ edits are satisfactory.

9. It would be useful to move the justification for why correlation/MRE were used to the start of the Testing and Validation section, as it wasn’t justified until after some results were already discussed and in the Supplementary Information.
• The authors’ edits are satisfactory.

10. The choice of which years/regions to highlight in Figures 3A and 4A should be justified.
• The authors response is satisfactory, but we suggest adding some of the rationale from the authors’ response into the text and/or figure captions directly as to why these scenarios were selected. We believe this will be a common question to arise from other readers.

11. The authors should comment on whether the migration insights derived from your model that are mapped in these plots can be unveiled through the existing approaches. What is the new knowledge your method unveils here (that with the raw training data or with prior approaches would either be unavailable or the models would have pointed to a different insight about migration)? Similarly, on page 8 before the Testing and Validation section, we believe adding an argument for how the uncertainties derived from the QuantMig study differ from the estimates derived for the same countries in the proposed approach. Specifically detailing new conclusions and compare/contrasting the knowledge insights between the two methods.
• The authors’ edits are satisfactory.

12. The strongest weakness of the study is the contradiction with the authors’ claiming innovation on bringing a sophisticated machine learning (ML) approach (recurrent neural networks) to the problem of predicting migrations vs. not

actually including a deeper literature review on ML-based approaches to human migration and choosing to benchmark only against a single paper / approach QuantMig. The QuantMig method, which the authors use as their baseline, does not cite existing technical approaches for this predictive problem or have a proper literature review and hence it is difficult to understand why QuantMig is the best baseline for the approach presented here. Therefore, this submitted manuscript is missing a proper literature review, especially as it relates to proposing a machine learning model (recurrent Neural Networks) as the main method's contribution, and hence, prior ML approaches to migration prediction should be addressed.
• The authors' edits are satisfactory.

13. There is a brief mention of "gravity models", but no elaboration beyond a citation to a single discussion paper. There are many studies that build statistical / ML models to estimate migration, some purely for migration research, others in the context of studying migration-based questions, such as forced migration under, for example, sea level rise under climate change, and those (often machine learning) models apply to the general migration estimation problem. Here are several papers that all proposed ML-based approaches relevant to modeling human migrations and the broader topic of human origin-destination flows modeling, any of which would be just as valid if not better baseline for the presented approach than QuantMig (many of these approaches can provide uncertainty estimates in one for or another, either directly through the model or from bootstrapping train/test splits etc)
• The authors' edits are satisfactory.

14. The QuantMig approach benchmarked here was not even re-applied to the full global dataset used in this study, but only the reported estimates for the 30 countries that the QuantMig paper focused on were compared. Hence, from a methodological perspective, there is no apples-to-apples comparison on the same dataset and label (and train/test splits) used for the proposed approach here to other ML-based methods applied to the exact same data. We request that such a methodological benchmark is appropriate to be provided to establish the relative strength of the new method. This does not take away from the contribution of compiling the global migration dataset and variables that the authors provided here, but still leaves open to questions whether the recurrent Neural Network method proposed is significantly better than other ML-based approaches proposed in the past.
• We thank the authors for explaining the QuantMig framework in more detail. We believe the authors' edits in the introduction could be further improved by including a version of their last sentence in the author rebuttal into the paper to make it clear that QuantMig is not easily transferable to other migration corridors.

15. On page 8, you make a reference to the target data. What is the target data?
• We thank the authors for their explanations. We believe editing the text of the main text to match the author rebuttal text, specifically "comprises the data (stocks, flows, net migration values) that are used to construct the loss function" would improve readability and understanding from a multi-disciplinary audience.

16. You made a reference to Figure 5D on page 8, but we believe this should be 5C
• The authors' edits are satisfactory.

17. On page 8 before Testing and Validation, we believe adding an argument for your presentation of the uncertainty.
• The authors' edits are satisfactory. The pertinent section in the Appendix is on page 22, "Uncertainty quantification". However, we note that we believe the text in the Appendix (Page 22) is still not precise enough to be able to fully reproduce the approach (e.g., it only says for the covariates uncertainty to "draw n = 100 samples of the initial stocks to estimate overall uncertainty"). For example, in the code section titles "Example: uncertainty quantification by sampling inputs" provided by the authors, it says "Here we illustrate the principle, by using a standard deviation of 5% on all covariates except the stock data, where we use 10%. Binary covariates are not sampled." The authors add this new text "We develop our uncertainty quantification approach at the global scale by hand-picking high-quality migration flow sources with similar definitions and adjusting the loss function to account for differences in data uncertainty, while propagating migration stock uncertainty through variation in initial stock sampling and our ensemble training." We note that picking high-quality sources and adjusting the loss are NOT strategies for uncertainty quantification; they are strategies for minimizing uncertainty in the model. Hence, we advise authors to rephrase the sentence.

18. Figure 6A - Is the first chart DESA Stocks? Is this a training/testing dataset?
• The authors' explanation is satisfactory.

19. Page 7 appears to be missing a reference to citation 34, when mentioning the WPP.
• The authors' edits are satisfactory.

20. We recommend reminding the readers what µ is on page 7, as it is introduced very early on in the paper.
• We believe our suggestion helps improve readability to an interdisciplinary audience. If the authors feel strongly about not including our suggestion, we don't believe it is important enough to impact acceptance.

21. We recommend adding some sort of indicator on the size of your datasets that you are using.
• The authors' edits are satisfactory.

22. In the introduction, it is unclear how the digital trace data from Facebook is calculated and ultimately measures migration. Additionally, what are the biases of using this data? Communicating that briefly, as mentioned with the labour migration and UN DESA data sources, would be a good addition.
• The authors' edits are satisfactory.

23. Page 14 says there are 4 sources, but then only lists 3 items in the list.
• The authors' edits are satisfactory.

** Minor Notes on revised manuscript:
24. On Page 23: "The optimal offset b_ij ∈ R of the initial stock … between observed and predicted stocks:"
• We believe you meant to say p_ij instead of b_ij

(Remarks on code availability)
The codebase for this study is fully open source and available on GitHub. The documentation for how to utilize the code in the README file is thorough and easy to follow. Links to the dataset are included, enabling complete reproduction of the results of the paper.

One thing that is somewhat unclear is that some parts of the code (Evaluation script) are named "Example ..." or "Demo ...", which seems to suggest they are not the exact procedure used for the analysis shown in the manuscript, but a simplified or reduced demo version. If that is the case, the documentation of those pieces of code should include precise instructions for setting up the code to reproduce the results presented in the paper exactly.

Referee #5

(Remarks to the Author)
I co-reviewed this manuscript with one of the reviewers who provided the listed reports.

(Remarks on code availability)

Version 2:

Reviewer comments:

Referee #1

(Remarks to the Author)
The authors have adequately addressed our comments.

The revised manuscript will make a solid contribution to research un this area.

(Remarks on code availability)

Referee #2

(Remarks to the Author)
I co-reviewed this manuscript with one of the reviewers who provided the listed reports.

(Remarks on code availability)
We did not review the code this time, as it was reviewed in prior rounds of review

Open Access This Peer Review File is licensed under a Creative Commons Attribution 4.0 International License, which permits use, sharing, adaptation, distribution and reproduction in any medium or format, as long as you give appropriate credit to the original author(s) and the source, provide a link to the Creative Commons license, and indicate if changes were made.
In cases where reviewers are anonymous, credit should be given to 'Anonymous Referee' and the source.
The images or other third party material in this Peer Review File are included in the article's Creative Commons license, unless indicated otherwise in a credit line to the material. If material is not included in the article's Creative Commons license and your intended use is not permitted by statutory regulation or exceeds the permitted use, you will need to obtain permission directly from the copyright holder.
To view a copy of this license, visit https://creativecommons.org/licenses/by/4.0/

Response to reviewers

Deep learning four decades of human migration

Thomas Gaskin[1, 2], Guy J. Abel[3]

[1] Department of Methodology, London School of Economics and Political Science; [2] Department of Applied Mathematics and Theoretical Physics, University of Cambridge; [3] Department of Sociology, University of Hong Kong

---

General comments

We are very grateful to the reviewers for their thoughtful and detailed comments, which we have worked into our manuscript and which have substantially improved the work. Please find a detailed response to each point below. In addition to the reviewer comments, we have made a number of improvements to the code and model, which have sped up training time by about 30%. We have dropped the `Refugees_diff` covariate, which is redundant, since it is merely tracking the change in the refugee figures and will thus be captured anyway by the RNN memory. It was for this reason that the model was least sensitive to this covariate in the original version. We have re-trained the ensemble, leading to a slight improvement in overall accuracy. Data is now hosted on huggingface, making integration with the git repository, as well as semantic versioning, easier.

Reviewers 1 & 2

1. *While the approach is novel, it raises several questions about the method, the data, the accuracy of prediction, and other aspects that limit its potential contributions in its current form. First, how do current data limitations shape these estimates? The bulk of the learning is occurring using data from a small subset of locations, primarily in Europe and the Global North, which are more reliable than in other areas. This feature generates more uncertainty about the migration estimates in regions of the Global South. That is not a weakness of the proposed algorithm, given that the data estimates the authors compare have high uncertainty. Our concern is about how this algorithm performs when it is asked to be pushed further into the future: Does training these models using data of the highest quality in one part of the world create path dependencies later on that will be misaligned with reality?*

   This is an important question, and one we have spent some time reflecting on and analysing from a number of different angles.

   First, we have analysed the geographic distribution of our target data—see the newly added Figure S6 in the appendix. Almost 80% of the target data are Facebook flow and UN DESA stock data, which have global coverage, though the latter is markedly less dense across Africa (see panel S6**A**). This is in part due to missing data, and in part due to a smaller diversity of migrant populations in these countries. The Finnish, Swedish, New Zealand, and QuantMig datasets only make up around 20% of target values. Europe is the region with the highest number of data points (around 150,000)—almost twice as many as the second-largest region (Sub-Saharan Africa, 87,000). However, Sub-Saharan Africa, Latin America and the Caribbean, and the Middle East together already have as many total target data points as the entire Global North. The inset in Fig. S6**E** shows that around a third of stock and

flow target points are South-South data, i.e. connecting two Southern countries; more than two-thirds in each are North-South flows; less than a quarter are fully Northern flows and stocks.

Second, in Figure 1**A** (next page) we show the variance on the net migration estimator $\hat{\boldsymbol{\mu}}$ as a function of the number of data points available for each country. Recall that since $\mu_k = \sum_{j,i} T_{ijk} - T_{ikj}$, if T_{ijk} are assumed i.i.d. Poisson distributed with variance λ_{ijk}, we have

$$\mathrm{var}\ \mu_k = \sum_{i,j} \lambda_{ijk} + \lambda_{ikj},$$

hence it is reasonable to expect the variance on the net migration to also reflect the uncertainty on the flows. As shown, the relative uncertainty on $\boldsymbol{\mu}$ does drop by about 0.02% per data point added, demonstrating that the uncertainty on our estimator reflects the range of possible migration flows that plausibly fit the observations.

Third, in order to assess the degree of Northern migration patterns being projected onto the South, we compared the test performance of our model on the different developmental regions. In response to Reviewers 4 & 5, Q8, we used 5-fold cross validation to evaluate performance, and show the prediction performance on each fold by developmental region in Fig. 1**B**. North-South flows are reproduced the most accurately, followed by South-South flows. While it at first glance may appear surprising that North-North flows are significantly *less* accurate, there is in fact a simple explanation: this is the region for which we have several conflicting data sources, and the neural networks must therefore 'balance' more information than for the global South, for which we are broadly relying on stocks and Facebook flows; see also Rev. 3, Q5 and figures therein. Performance on South-South flows is typically around 15-20% worse than for North-South flows, which may be a reflection of the lower training data quality, but could also indicate data leakage.

Fourthly, to further investigate this question, we trained a new neural network and withheld the QuantMig, Swedish, Finnish, and New Zealand (QSFN) flow data. The goal is to understand the dependence of the various regional estimates on Northern migration data. Ideally, we would need to train an entire ensemble of neural networks to fully quantify the dependence of non-Western estimates on Western-sourced training data, but the results with just one realisation are already fairly insightful. In Fig. 1**C** we plot the distribution over the change in flow estimate relative to the uncertainty,

$$\Delta_{ij} = \left\langle \frac{\left| \hat{F}_{ij}^{\text{original}} - \hat{F}_{ij}^{\text{no QSFN}} \right|}{\mathrm{std}(\hat{F}_{ij})} \right\rangle_t \tag{1}$$

where $\langle \cdot \rangle_t$ represents the time-average. Dividing by the uncertainty is necessary since we are comparing a single realisation to a distribution. We see that South-South flows are disrupted the least, with a median of shift of 1.4σ; North-South flows and North-North flows are disrupted by 3.4σ and 2.7σ, respectively. The geographic distribution of the change is given in Fig. 1**D**, with the largest deviations occurring across Scandinavia and New Zealand (unsurprisingly), as well as continental Europe. In this panel, we show

$$\frac{1}{2}\left[\mathrm{Median}_i \Delta_{ij} + \mathrm{Median}_j \Delta_{ij}\right], \tag{2}$$

though showing mean values for just the origin or destination yield much the same plot.

Lastly, we also trained a new neural network only on data from 1990-2015, withholding the entire period from 2015 as a validation period. This is to analyse the question of path dependencies raised by the reviewers, and to understand whether the data produces any noticeable differences in forecasting ability across regions. This experiment means withholding the Facebook data, causing the target flows to be made up entirely of North-South and North-North flows. The results are given in plots 1**E**: we again show the change eq. (1) to the flow and the stock estimates for the

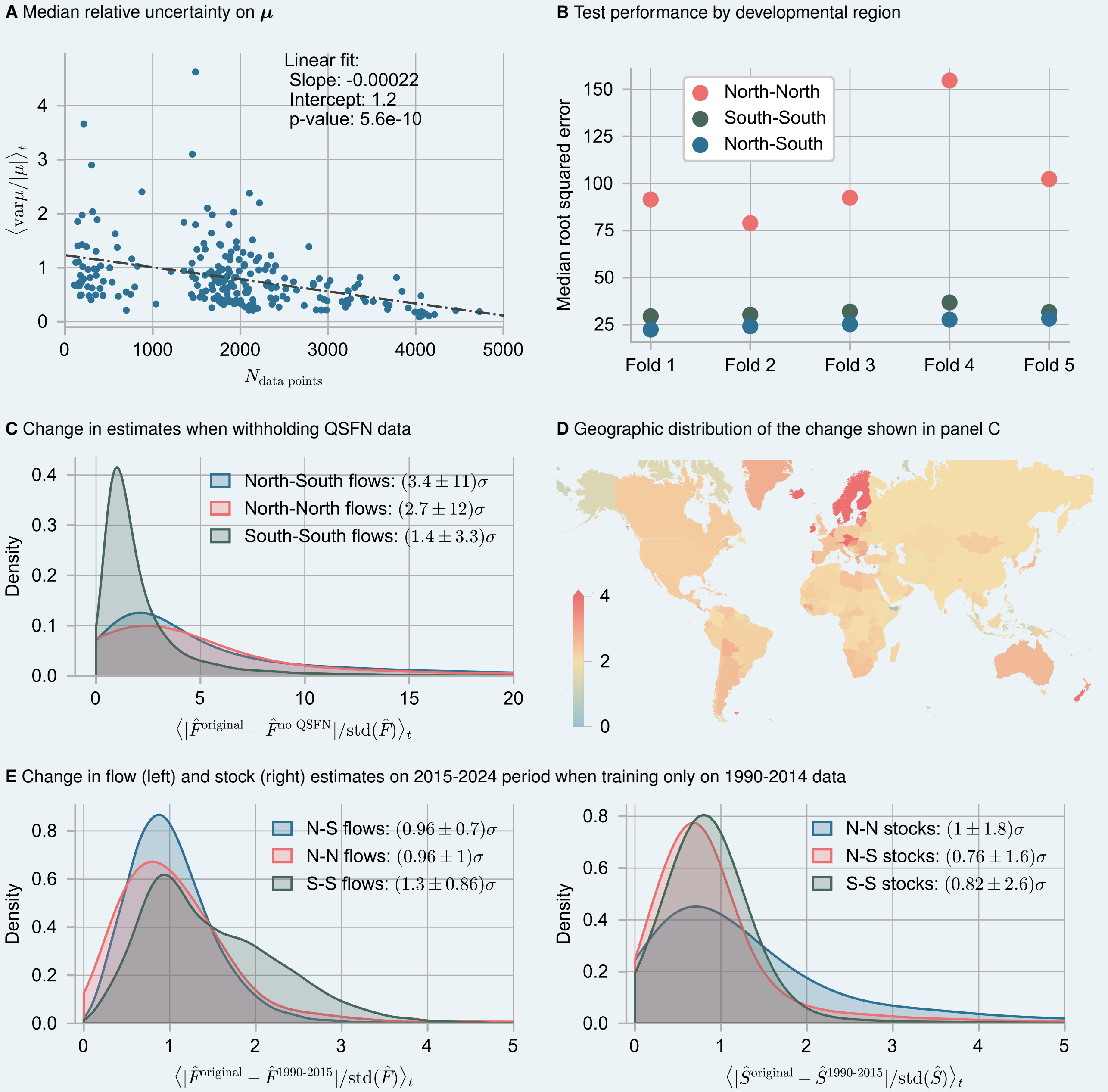

Figure 1: Investigating bias in the estimates with regard to development level. **A** Relative uncertainty on the net migration estimate μ (y-axis) as a function of the number of target data points available for each country (stocks, flows, and net migration values). Sweden, Finland, and New Zealand are excluded—they each have around 15,000 data points with a median relative uncertainty of between 10% and 20% (see Fig. 5**C**, main manuscript). Also shown is a linear fit and its parameters. **B** Median root squared error on flow corridors for all five test folds, by developmental region. **C** The change in estimates when withholding QSFN data. Shown are densities over the median absolute difference for each corridor (median taken over time), in units of the standard deviation. **D** The same figures as in panel C, this time additionally taking the median over both the destination and origin country and averaging to obtain a single value for each country. **E** The same figures as in panel C, this time when training without data from 2015-2024. Shown are the densities on both the flow estimates (left) and the stock estimates (right).

period 2015–2024. Note that this period contains the Covid drop, but we found that restricting ourselves to only using the years 2015–2020 as a validation period did not change the result. The plots indicate that the network's

forecasting ability is roughly the same across developmental regions—slightly worse for the global North than the global South for the stocks, slightly better for the flows.

One final point to consider is that the question of bias is not just related to the targets, but also the *input* data. The observation data $\boldsymbol{Y}$ is after all not a modelling choice—the observations are given, what is a choice are the input covariates $\boldsymbol{\chi}$. The inference problem (eq. (2) in the manuscript) is severely underdetermined due to the lack of data. There are many flow tables $\hat{\boldsymbol{T}}$ that mathematically reproduce the target flows and stocks; however, many of these are wholly unreasonable solutions to the inference problem. For instance, a flow table moving 100,000 people from Malta to Fiji in one year and 99,900 back the next may *mathematically* fit the observations, but is incompatible with geographic and political realities. As with gravity models, we thus take the approach to force the solutions to take the form of a mapping

$$\hat{\boldsymbol{T}} = u_\theta(\boldsymbol{\chi}), \tag{3}$$

with $\boldsymbol{\chi}$ the covariates and u_θ a neural network. Geographically at least, all countries are represented equally in the input data, which is a complete set of 17 covariates for each country and year. Now let $p_\chi(u_\theta|\boldsymbol{Y})$ be the distribution of possible functions mapping the covariates $\boldsymbol{\chi}$ to flow tables $\boldsymbol{T}$ given the observables $\boldsymbol{Y}$; ensemble training naturally provides an unbiased (in the statistical sense) estimator of p_χ, since in the limit of infinitely neural networks with iid. random initialisation, we naturally recover p_χ. But the real question is, is our choice of $\boldsymbol{\chi}$ the 'right' one; in other words, for a fixed $\boldsymbol{\chi}$, is the distribution $p(u_\theta|\boldsymbol{\chi}, \boldsymbol{Y})$ biased? We cannot answer this question directly, since we do not know the 'true' covariates $\boldsymbol{\chi}^0$ (i.e. the true drivers of migration). Indeed, many migration scholars argue against the possibility of a universal set of determinants for a comprehensive theory of migration (which we might use for estimation in our context), emphasising the necessity of context-dependent mechanisms [1–3] instead.

We thank the reviewers for raising this interesting question, and hope the additional numerical results prove insightful. We have added much of this above discussion to the SI, with a short paragraph in the main text (under 'Testing and Validation' sumarising the results).

2. *Moreover, since this is a new method, can the authors test the accuracy of the predictions using a simulation? Given the aforementioned problem, it would be beneficial to understand how sensitive the algorithm is to this issue of missing/noisy/low-quality data. It would not be impossible to generate world-year estimates and remove (or mess up) observations in ways that reflect our current state of data collection. We recognize that this is a significant amount of work, but since this is a new tool, we want to understand the scope conditions under which it is likely to provide reliable estimates and under what data circumstances it yields totally inadequate estimates. Knowing the promise and limits of the tool will make us more confident about its application. As is, we are less confident about these findings based on the authors' approach of benchmarking against existing data sources alone.*

   This was already done in the original manuscript: in the SI under 'Validation on synthetic data' (pp. 14ff), we generated a set of synthetic flows and carefully analysed the model performance. Figures S18–S21 show the results; in S19, we use noiseless and fully observed data, showing that we recover the originally used flow matrix in panel **c**. In S20, we use a high level of noise and mask out a significant proportion of the data—see the text on page 16 for a description. This demonstrates that, in the limit of low noise and fully observed data, $\boldsymbol{T}$ is uniquely inferred. Since we are, of course, not operating in the fully observed, noiseless regime, ensemble training should gauge the degree to which the problem is ill-posed (i.e. how many equally good solutions to the problem there are).

3. *We appreciate the modeling of confidence bounds and the discussion on meaningful uncertainty quantification, but given the global scope of this paper there is significant heterogeneity within and across regions (i.e., continents, sub-regions, etc.) that needs more scrutiny and discussion. We don't get a clear sense of how this uncertainty might vary across countries and regions, etc., so there is no clear takeaway from this approach.*

   We thank the reviewer for raising this point. In fact, our results already illustrate some regional variation in uncertainty (see section on Meaningful uncertainty quantification and Fig. 5**c**). Note that, by the argument given in

response to Q1, analysing the uncertainty on the net migration is sufficient to gauge the uncertainty on the full flow table $\boldsymbol{T}$, since the T_{ijk} values can broadly be assumed to be i.i.d Poisson. The geographic distribution of the variance on $\boldsymbol{\mu}$ in Fig. 5**C** should for this reason be understood as representative of the overall uncertainty. However, we agree that a more general takeaway was not made sufficiently explicit in the text. We have revised the discussion to highlight that uncertainty is systematically wider in regions with sparse or volatile data (e.g., Sub-Saharan Africa, Pacific Islands) and narrower in regions with more stable migration systems and stronger statistical infrastructures (e.g., intra-European flows).

4. *Figure 1: While it illustrates the challenges of measuring migration, it is unclear how widespread these challenges are, where the challenges are most severe, etc. In other words, it raises a concern without documenting the extent and nature of this concern more carefully.*

   The challenges are very real, and we have added some further explanation and references to point readers towards a more thorough documentation of the problem. Figure 1 is merely intended to illustrate the situation, and it is beyond the scope of our manuscript to conclusively demonstrate that global migration data is inconsistent, difficult to measure reliably, and in need of harmonisation; however, in Fig. 2 we have assembled some additional plots that hopefully serve to underline our point. Fig. 2**A** shows the relative uncertainty on the QuantMig estimates in Europe; Fig. 2**B** shows the median relative difference between US Census Bureau and UN WPP net migration estimates; note that France, the US, Brazil, China, and South Africa all have median relative differences of at least 50%. Fig. 2**C** shows the median correction to the UN DESA stock data between the 2020 and 2025 revisions. Note the high value for earlier years, including 1990. Figure 2**D** shows the revisions for the 5 largest migrant stocks in the US. Fig. 2**E** shows the median difference between the reporter values in the DemigC2C dataset (i.e. origin-side and destination-side). For all countries not in Scandinavia, the median difference is at least 50%, and in many cases exceeds 100%. These plots give a lower bound on the problem, since they mostly show European countries whose migration figures are generally assumed to be of high quality. Note that in all datasets we are showing median values. See also Fig. S3 in the appendix.

5. *Figures 3–5: The countries and regions featured in these figures appear to have been selected ad hoc. There is no clear rationale as to why certain countries or regions are highlighted and not others. As a result, the findings are illustrative but not conclusive, ultimately undermining the paper's contributions.*

   Countries and regions were chosen with the aim of covering a broad range of migration contexts in the paper overall—geographic regions, development levels, sizes of flows, and degrees of temporal variation—while also allowing comparison with external datasets where available. This necessarily requires some selectivity, but the choices were guided by the need to illustrate the model's behaviour across contrasting situations rather than to highlight particular outcomes.

   In Figure 3 we focus on Europe for comparison purposes with QuantMig and national statistics. Within this region we selected flows that reflect both large volumes (e.g., Poland–UK), strong temporal fluctuations, and cases where national statistical sources for Sweden and Finland allow a second layer of comparison. These considerations explain the inclusion of Hungary–Finland and Czechia–Sweden despite their smaller sizes. Figure 4 turns to Africa, where UNHCR and UN DESA provide the most consistently available external data. The African cases were selected to span both major regional hubs and corridors known to be difficult to estimate—for instance Nigeria, whose migration patterns are highly uncertain and therefore informative for stress-testing the model.

   Because Figures 3–4 concentrate on Europe and Africa, Figure 5 introduces large and influential countries in South, East, and Southeast Asia—China, India, and Vietnam—to ensure representation of regions otherwise absent from corridor-level illustrations. The remaining panels highlight major global actors (e.g., Russia) and cases illustrating alignment or divergence between our estimates and UN sources (e.g., Iraq). Taken together, Figures 2–5 provide coverage across Europe, Africa, the Middle East, South Asia, East/Southeast Asia, and parts of Latin America.

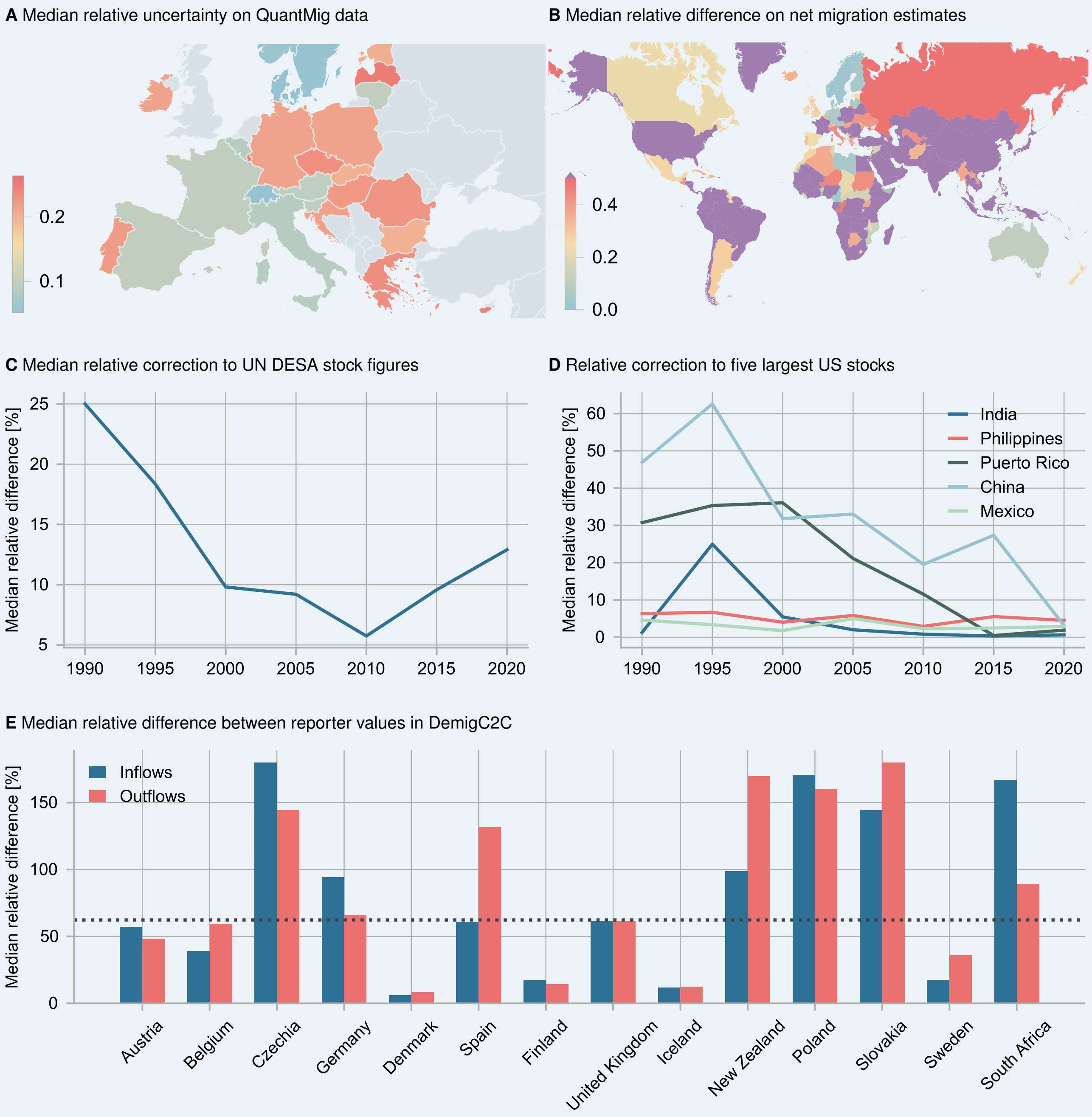

Figure 2: Complementary figure to Figure 1 in the main manuscript, further illustrating the extent of uncertainty on international migration flow data. **A** The median relative uncertainty on intra-European QuantMig flows for each destination country; in many cases, the median uncertainty is well above 20%, and is rarely below 10%. **B** Median relative difference between the US Census Bureau and UN WPP net migration estimates. Countries with a median relative difference over 50% are marked in purple. **C** Median relative stock figure correction between the UN DESA 2020 and 2025 revisions, in %. **D** Relative stock figure correction for the five largest migration stocks in the US, in %. Note the large revisions for earlier stocks, despite the revision occurring between 2020–2025. **E** Median relative difference between the reporter values (i.e. origin-side and destination-side) for all countries in the DemigC2C dataset, in %. Shown is the difference for both inflows and outflows, and the median is taken over all years, methods, and criteria (i.e. country of birth/country of residence). Also marked is the overall median (dotted line).

Oceania and Small Island States are omitted because their UN net-migration series are typically flat or driven by small absolute numbers, offering limited diagnostic value for evaluating uncertainty.

Any finite subset of examples is unavoidably somewhat ad hoc; however, the set used here is constructed to maximise geographic and substantive diversity across the paper within tight space constraints. We would also like to note that our choice of visualising a large, global dataset via a selection of corridors is quite common; see, for example, [4–7]. Crucially, the paper's main results do not depend on any single corridor. The global net migration comparison in Fig. 5 offers a non-selective benchmark that complements the illustrative corridor-level panels and ensures that the conclusions are not sensitive to the particular examples highlighted. Figures 6 and S22 provide dataset-level analysis validation results that provide the primary support for our approach and findings. We have also changed the selection of edges in Fig. 2**C** to now show the six largest corridors by total flow (summed over the entire period).

6. *On an editorial note, we need better legends, consistency in axis scales, and clear labels in the figures to enable readers to understand these findings.*

   We have updated the figures accordingly. Since the flow values typically span several orders of magnitude, panel-specific and even subpanel-specific y-scales are unavoidable. However, all panels in figure 2 now show flows in millions; in figures 3 and 4 we have changed the scientific notation ($1e5$) to a more familiar $\times 10^5$-type notation. We decided that showing all flows in thousands would be confusing in cases when the y-scale would still need to be scaled by several orders of magnitude. 5**A** is now entirely given in millions; in figure 6 we have added a colour legend and made the axis labels clearer. The subpanels 2**C**, 3**B**, 4**B**, 5**A** share an x-axis across columns, hence we still use a shared x-axis and omit the x-label since it is clearly indicating years. This in our view produces a visually more appealing and less cluttered plot while maintaining legibility. Similarly in the SI, where we have updated the scientific notation.

7. *The paper relies heavily on published papers by one of the authors to establish key data and methodological details. These citations are included in the references, but a full understanding of many key issues requires reading the referenced papers as well. This over-reliance on prior publications places undue burdens on the reviewers. Since the paper is highly technical, more details must be provided in full in this manuscript to help reviewers understand this approach and to assess its merits, especially those who are not familiar with this methodological approach (and its potential limitations).*

   Our method is wholly independent of previous methods. We compare our methods to previous approaches to estimating five-year flows which have been developed by many authors over the years and comparatively assessed, but not all developed, by Abel et al. in previous work. Our method is independent of these previous approaches; we only draw on them for benchmarking purposes and to give the reader methodological context. We have now added a description of each comparison method at the start of the Methods subsection on Quantifying global migration.

8. *We could not replicate the analysis. The problem seems to be missing data very early in the code. You can see the path errors in `Data/__init__.py`. For example, `world_shapefile/ne_50m_admin_0_countries.shp` is missing, and the entire `Flow_data` folder (`Flow_data/QuantMig_data/Quantmig_flows.nc`) could not be found. To our knowledge, these files are not available on GitHub or Zenodo. It is possible that these files were available on another path or generated from data available in these sources, but no explanation was provided for how to generate them.*

   We have uploaded al the missing files, and thank the reviewers for pointing this out. All the files and notebooks should now work correctly, provided all datasets have been downloaded. Note that the data and trained networks have been entirely moved out of the Github repository to huggingface.

Reviewer 3

1. *Main text, p. 2, left column: The United Kingdom has been phasing out reliance on passenger surveys - better rephrase as "rely on visa records, administrative data, and until recently passenger surveys." In addition, some countries use surveys (such as the Labour Force Survey).*

   Amended.

2. *Main text, p. 3, left column: When you introduce the loss function, it would help defining it in intuitive terms, to aid the more generalist readers of the paper, as a function that gives weight to the discrepancy between the model-based estimates and the data, or something similar.*

   Added.

3. *Main text, p. 4, left column: The argument about long memory can be seen as controversial and merits a more thorough discussion. In the migration forecasting literature (Bijak, Wiśniowski), one of the common findings is non-stationarity, which implies short memory, and ever-changing equilibria. To that end, using a whole 40-year training period may seem counterproductive. In this context, to make the argument more nuanced and also consistent with what is known about migration processes, it is worth decoupling the lack of stability (non-stationarity) of migration processes over time from some persistence of patterns across space (gravity). One lens to look at that could be offered by the generation-distribution framework [Willekens F (1994) Monitoring International Migration Flows in Europe, Eur J Pop, 10(1): 1–42]: you are concerned with the distribution aspect here.*

   We thank the reviewer for raising the important point. The recurrent neural network is not equivalent to a time series model with the entire 40 year training window functions as a quasi-covariate. Rather, the RNN maintains a single hidden state $z_{ijk}(t)$ which is updated every year. The length of the correlation structures learned may vary, and could be extremely short, since the effect of previous years can be overwritten. It is thus perfectly possible that the learned correlation lengths are no more than a few years. Our model design simply gives the neural network maximum flexibility in how much information it chooses to retain from the past. We have added text to the introduction clarifying our use of the term memory in the context of often non-stationary migration flows.

4. *Main text, p. 4, right column: do the UNHCR asylum data that you use include temporary protection? If not, or not precisely, this may explain why you do not pick up the Ukraine to Poland flow in 2022 as the largest one (1.568 million temporary protection decisions granted between 4 March and 31 December 2022 as per Eurostat). I suggest rewriting this fragment to avoid suggesting that the main outflow from Ukraine was to Russia, which it was not: here, context is everything.*

   We thank the reviewer for pointing this out; indeed, as stated in the Methods section, the UNHCR data only contains "refugees under UNHCR's mandate", "asylum-seekers", "other people in need of international protection". Our estimate of Ukraine to Poland flows in 2022 is around 720,000: this figure is most likely informed from the Facebook estimate (around 650,000). However, since retraining the ensemble, the Rwanda to DRC outflow ranks as the largest, before Venezuela to Columbia and Ukraine to Poland, Germany, and Russia, so we have removed the statement on flows from Ukraine entirely.

5. *Main text, p. 6, Figure 3: The alignment of your estimates with the QuantMig ones in the presented examples is remarkable. Are there any flows for which the alignment is much weaker, and are there any lessons (with respect to data availability or features of countries) that can be drawn from that?*

   Good alignment is not surprising, as the QuantMig data is part of the training targets. In Fig. 3**A** we show the median absolute deviation between our estimates and the QuantMig data in standard deviations for each destination

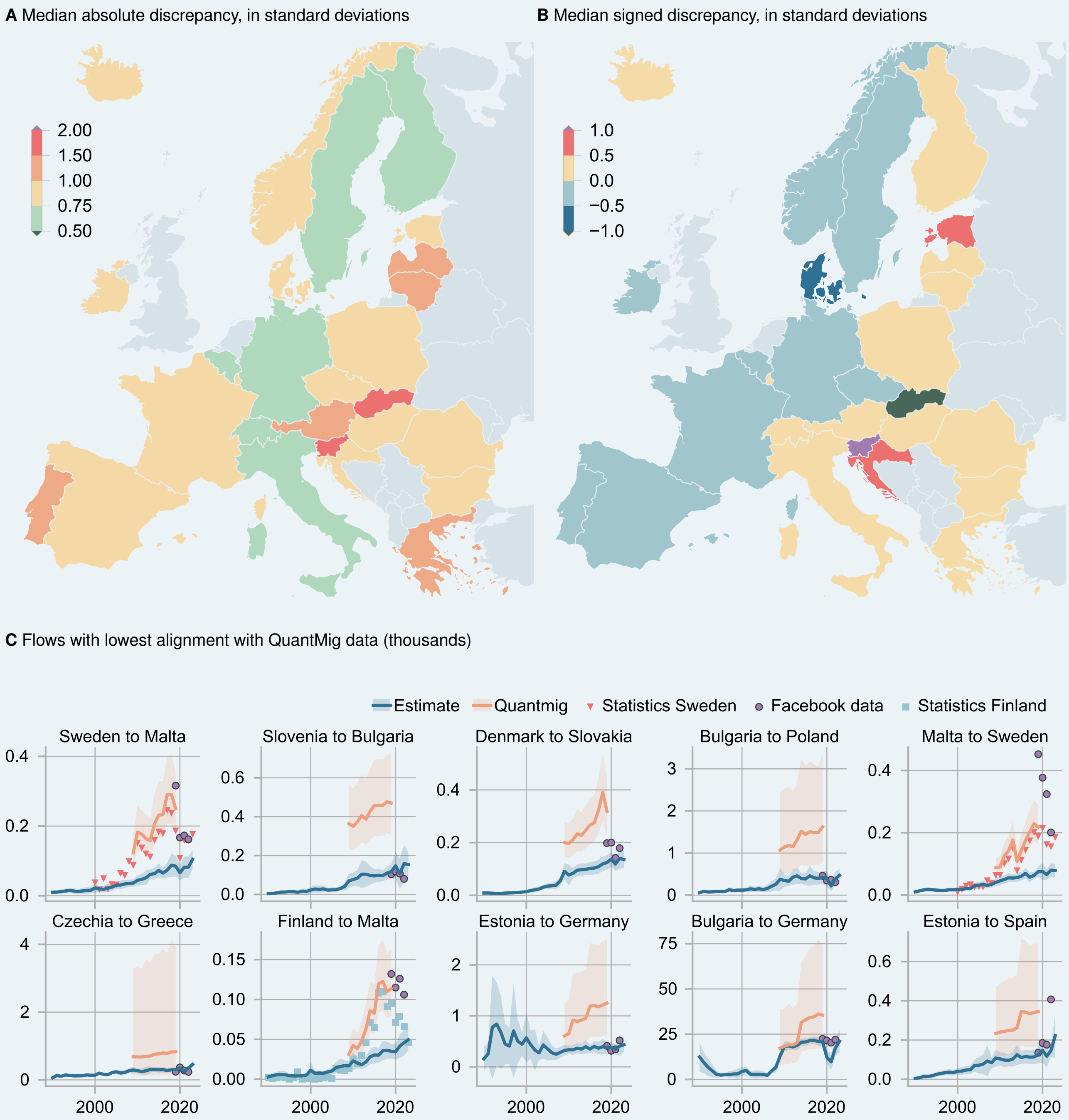

Figure 3: Overall alignment of our estimates with the QuantMig data. **A** The median absolute discrepancy between our estimates and the QuantMig values, using eq. (4). **B** The same plot as in panel A, using the signed discrepancy eq. (5). A negative value means our estimates are lower than the QuantMig values. **C** Flow estimates (in thousands) for the ten corridors with the highest median absolute discrepancy with the QuantMig values (median taken over time). (Continued next page)

country,

$$\mathrm{med}_{i,t}\left[\frac{|\hat{F}_{ij} - F_{ij}^{Q}|}{\mathrm{var}(F_{ij})^{1/2}}\right] \tag{4}$$

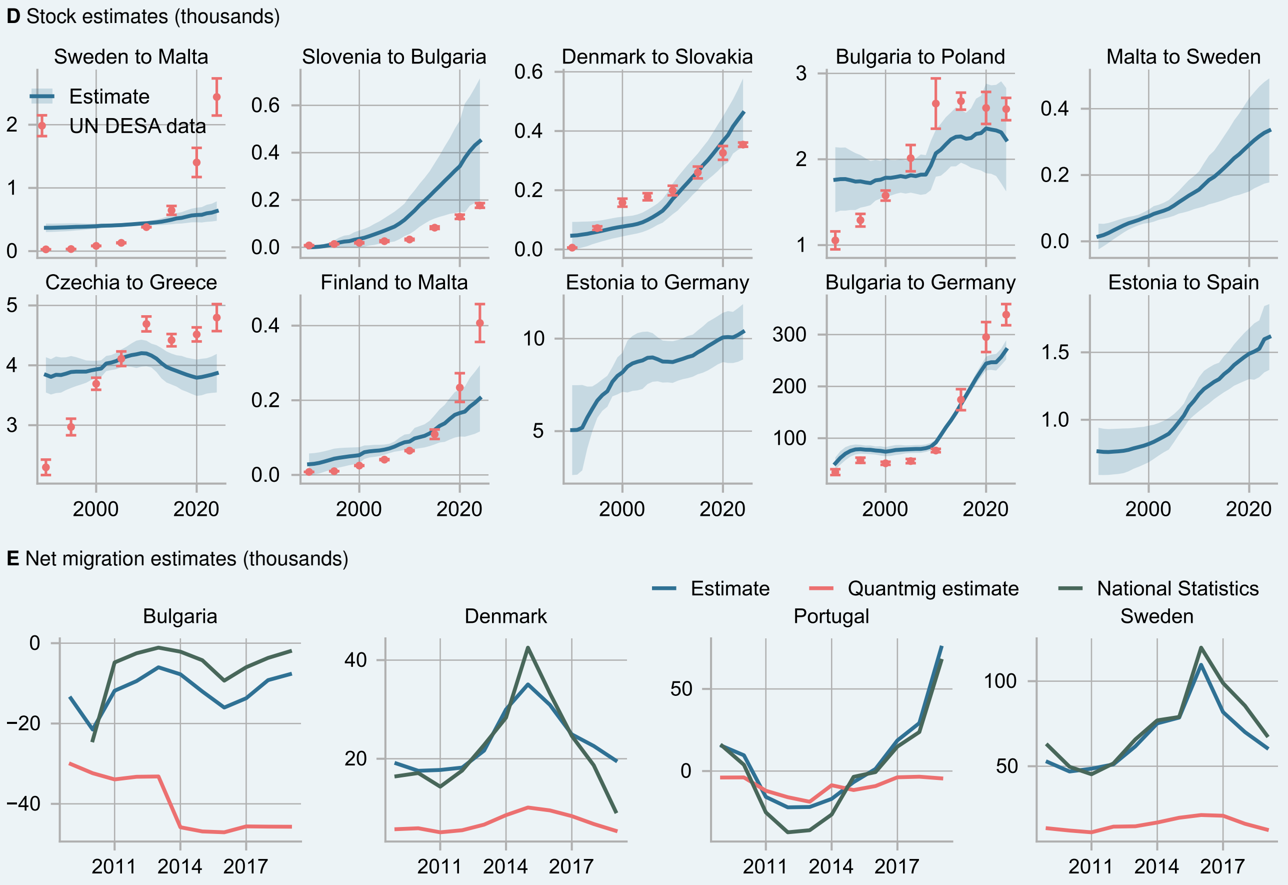

Figure 3: **D** Stock estimates for the same ten edges as in C. **E** Net migration estimates for Bulgaria, Denmark, Portugal, and Sweden. The QuantMig estimate is calculated by only considering flows modelled in that dataset and does not include non-European migration; it is thus only intended for comparison purposes.

(that is, the median is taken over the year and the *origin* country, but taking the median over the destination instead yields a very similar plot). Generally, the discrepancy between the estimates is within one standard deviation (using our standard deviation, which is more conservative than the QuantMig values), and there is no clear geographic pattern as to the performance in absolute terms. One pattern we did discover is shown in Fig. 3**B**: here, we are showing the *signed* median discrepancy for each destination country, i.e.

$$\mathrm{med}_{i,t}\left[\frac{\hat{F}_{ij}-F_{ij}^{Q}}{\mathrm{var}(F_{ij})^{1/2}}\right]. \quad (5)$$

A negative value means that our estimates tend to be *lower* than the QuantMig values, a positive sign indicates a higher flow. We found that, in general, our flows tend to be lower than the QuantMig estimates for Northern Europe and Western Europe, and higher in Southern and Eastern Europe.

There are several edges for which alignment is bad—the ten corridors with the lowest alignment are shown in Fig. 3**C**. The corridors are mainly small (except Bulgaria to Germany), and the discrepancy can often (though not always) be explained by conflicting values between the Quantmig and Facebook data. For Sweden to Malta, our estimates are much smaller than both the QuantMig and official Swedish statistics (which are probably broadly overlapping datasets in this case). We found that for very small flows (less than 10) we are consistently higher than the QuantMig

estimates, but for flows above 10, especially flows between 10 and 1000, we are mostly lower than QuantMig, as can be seen. A linear regression of the signed error against the QuantMig flows yields a slope of -3.5×10^{-5}—only a very weak effect. In absolute terms, performance on small edges is only marginally worse than for large flows—we obtain a slope of -5×10^{-7}—which can be explained by the Yeo-Johnson transform. To conclude, overall alignment is good, since the QuantMig data are part of the training dataset, and most of our estimates fall within one standard deviation of the QuantMig data. In signed terms, most flows fall within $\pm 1\sigma$, but with a marked bias towards *smaller* migration flows.

As we discuss in Rev. 1, Q1, Europe is the region in which the model actually performs the *worst* on the test data; the reason for this is simply the large diversity of different data sources available, which are not always compatible with each other. In addition to three different sources of flow data, there is a high density of UN DESA stocks and net migration data available. These constraints cannot all be optimised simultaneously, and the misalignment with the QuantMig data is highest where there exists some incompatibility with some other source. In Fig. 3**D** we show the stocks corresponding to the 10 most misaligned European flows—we can see that in many cases, here too the performance is poor. The case of Czechia to Greece is informative: here the stocks and flows seem incompatible with each other, with the model attempting to fit both the increase in stock and the relatively flat flow data, and choosing a 'middle ground'. Then there are cases where stocks and flows *do* seem compatible: Sweden to Malta is an obvious case. We suspect that in these cases, the net migration data is acting as a constraint limiting the flows (Fig. 3**E**). One lesson to be learned is that strong misalignment to any one data source indicates incompatible target data values; another reason is that the information given in the input covariates is not sufficient to model *all* aspects of the dynamics (i.e. our chosen covariates might not explain the sharp increase of Swedes in Malta).

6. *Main text, p. 10, left column: Given the importance of life expectancy and GDP, have you considered using the Human Development Index instead, for the sake of slightly greater parsimony?*

   Indeed we did—HDI was one of our initial input features. However, it is not directly available for all countries for the period under consideration (such as Eritrea pre-2005, many Pacific Islands such as Tokelau or Niue, overseas territories such as the Falklands, etc.) and would thus need to be manually calculated for these countries anyway using economic, life expectancy, and education data (the latter especially are also difficult to obtain for earlier years). We thus decided to stick with HDI's constituent indices, though the idea that our feature importance ranking is essentially giving a proxy for HDI is eminently plausible.

7. *Main text, p. 10, right column: You make an interesting point about possible applications to forecasting, although I am not convinced that the past data are sufficiently informative to improve the inferences here: there are problems with predicting covariates, the stability of their (uncertain) relationships, and so on, not to mention the philosophical question of memory mentioned above. I think this merits a more nuanced discussion, which can be centred around the purpose of your model, which is estimation, as opposed to forecasting or explanation. This is where deep neural networks can really shine, and as a byproduct, you can argue that you offer a coherent and improved knowledge base for other types of studies.*

   We agree that migration forecasting faces challenges due to the instability of covariate relationships, the difficulty of predicting future drivers, and the non-stationarity of migration processes. We have revised the text to stress that the primary purpose of our model is estimation, and that its contribution lies in providing a coherent, finer-grained knowledge base (of annual global flows) than previous studies, which could potentially support research into migration forecasts and projections.

8. *Methods, p. 14: It is worth adding that equation (2) is a deterministic identity.* Added.

9. *Methods, p. 15, Figure 7: At the end of the caption, it is worth adding that (as I presume) the thicker the lines the higher the weights, to bring home the weight-uncertainty relationship.* Added.

10. *Methods, pp. 17/18: The targeting of stock differences rather than stocks themselves is novel—I would highlight this as an additional contribution vis a vis the state of the art.*

    This is also related to the last point in the Methods section ('Calibrating the initial stock value'), which is only possible since we targeting differences, not flows. Using absolute stocks as targets is ill-advised since it conditions the time series on the highly uncertain initial values.

11. *Methods, p. 18: The CeLU function is missing a definition on the left-hand side and in the text—readers unfamiliar with neural networks will not know which function it is and what it does (ie. activate a layer).*

    We have added a $\sigma(x) = \dots$ to the equation (the canonical symbol for a neural network activation function) and an additional explainer to the text.

12. *Methods, p. 18: The section on uncertainty quantification is a bit underdeveloped and is worth expanding. You mention model uncertainty and covariate uncertainty as the two key sources, but there is vast literature (including IMEM/QuantMig) on the role of measurement errors, definitional mismatches, and so on. You can of course define your two key sources (model and covariates) in such a way that they encompass all the other ones, but this needs to be made explicit. Similarly, given that your approach to UQ is rather simple, I would expect a longer critical discussion as to which aspects of uncertainty are included, which are not, and why what you propose is a "good enough" approximation of the total uncertainty (there is a similar argument in the QuantMig book - see G below).*

    We have added a paragraph to provide context and justify our UQ approach within the broader migration estimation literature. We note that we adapt our UQ approach to the global scale and the available data environment, rather than attempting to implement an explicit measurement model due to the lack of meta information in global-scale datasets and our approach to selecting target data with source-specific components of the loss function. For the latter, our framework incorporates flow information only from a hand-picked set of high-quality sources with relatively consistent definitions (e.g., New Zealand, Sweden, Finland, Facebook global estimates, selected net migration, QuantMig).

13. *Methods, p. 19: In (11), better denote the offset by a different letter to avoid any subconscious confusion with births or birth rates amongst demographically-minded readers.*

    We have changed it to ρ!

14. *The paper is very well grounded in the existing body of work on estimating migration flows. For completeness, you may also consider referring to the IMEM model [Raymer J et al. (2013) Integrated Modeling of European Migration. JASA, 108: 801–819], which was a direct predecessor to the QuantMig work, and its follow up in the Human Migration Database [Dańko M (2024) HMigD I App: The Human Migration Database I App. Version 3.3.1. Rostock: Max Planck Institute for Demographic Research. https://maciej-jan-danko.shinyapps.io/HMigD_Shiny_App_I]. For the QuantMig references themseleves [8,9], the corresponding published work is Chapter 5 (Aristotelous et al.) in: Bijak J (ed.) (2024) From Uncertainty to Policy: A Guide to Migration Scenarios. Cheltenham: Edward Elgar. https://doi.org/10.4337/9781035319800. The QuantMig book also includes arguments on non-stationarity, building on earlier work, especially Bijak J (2010) Forecasting International Migration in Europe: A Bayesian View. Springer.*

    We have added these references, and a few more, to the UQ section in response to the previous comment above. We have updated the QuangMig reference to the book chapter (and keep the additional reference to the data).

Comments on SI

1. *On p. 6, just under Figure S3, it is not clear where the fist (unnumbered) formula comes from: adding a sentence or two with an explanation would be very helpful.*

We thank the reviewer for pointing this out and have added an explanation. This formula assumes an exponential decay model for the population, i.e.

$$\text{Pop}^{\text{end of year}} = \text{Pop}^{\text{start of year}} \times (1-\gamma) \Rightarrow \text{Pop}^{\text{mid year}} = \text{Pop}^{\text{start of year}} \times (1-\gamma)^{1/2} \quad (6)$$

(with γ the mortality rate). One could alternatively assume a linear model, i.e. that a fraction $\gamma/2$ of the population dies in the first half year and the same fraction in the second, giving

$$\text{Pop}^{\text{mid year}} = \text{Pop}^{\text{start of year}} \times (1-\gamma/2). \quad (7)$$

Fortunately, when reversing this process (i.e. dividing by the rates on the RHS), we have the following Taylor expansions around $\gamma = 0$:

$$\frac{1}{\sqrt{1-\gamma}} = 1 + \frac{\gamma}{2} + \frac{3\gamma^2}{8} + \dots \quad (8)$$

$$\frac{1}{1-\gamma/2} = 1 + \frac{\gamma}{2} + \frac{\gamma^2}{4} + \dots, \quad (9)$$

rendering the question of which model to pick moot.

2. *On p. 7, before equation (2), it would help the reader either to repeat the formula for the loss function from the main manuscript, or at least provide a cross-reference.*

   We have added a cross-reference.

3. *On pp. 7/8, it looks like a lot of the information on the errors is borrowed from the QuantMig estimates for a subset of flows: if so, this is a strong assumption, which merits a critical comment.*

   Indeed: where flow weights are only partially observed, the error on the remaining values is set to the median observed error. For the flows, *all* uncertainty is taken from the QuantMig data, so the weights on all edges present in the QuantMig data are essentially either 1 (meaning no information) or determined by the QuantMig flows. Our thinking was that any other approach would lead to distortions, such as artificial jumps to 1 outside the QuantMig time domain, or giving artificially large weights to non-QuantMig values. QuantMig values would then in effect be "punished" for coming with uncertainty, and given less weight in the loss function purely by virtue of being weighted. To avoid this, we adopted the current approach, but we acknowledge that it projects much of the (Europe-centered) QuantMig methodology onto other values, and have added a note to this effect in the text. In Fig. 4, we show the Fig. S5 from the SI with the QuantMig weights overlaid. Interestingly, most of the 'extrapolated' weights are above 1. This could be interpreted as meaning that most of the edges for which we have data beyond the QuantMig times are higher quality data—leading to generally higher weights on those edges—but also that we are biased towards edges that have *some* QuantMig values. See also response to Reviewer 1, Q1.

4. *Throughout, I would explain acronyms, especially the more technical ones, on their first occurrence (e.g. CeLU, ReLU on p. 15—the former also in Methods, p. 18). Some other terms (Yeo-Johnson transform) can be cross-referenced to the Methods section.*

   We realised upon revising that the function is called CELU—a "C^1 (i.e. continuously differentiable) exponential linear unit"—and should thus be abbreviated as CELU. We have added a reference to the paper that introduces the function, rather than the PyTorch documentation; however, we doubt that giving the full name would be particularly enlightening to anyone not familiar with machine learning parlance; we have thus added explanations only to the SI, in view of the space restrictions on the main manuscript.

5. *In the main manuscript, "SI" needs expanding on the first occurrence as well (p. 2).* Added.

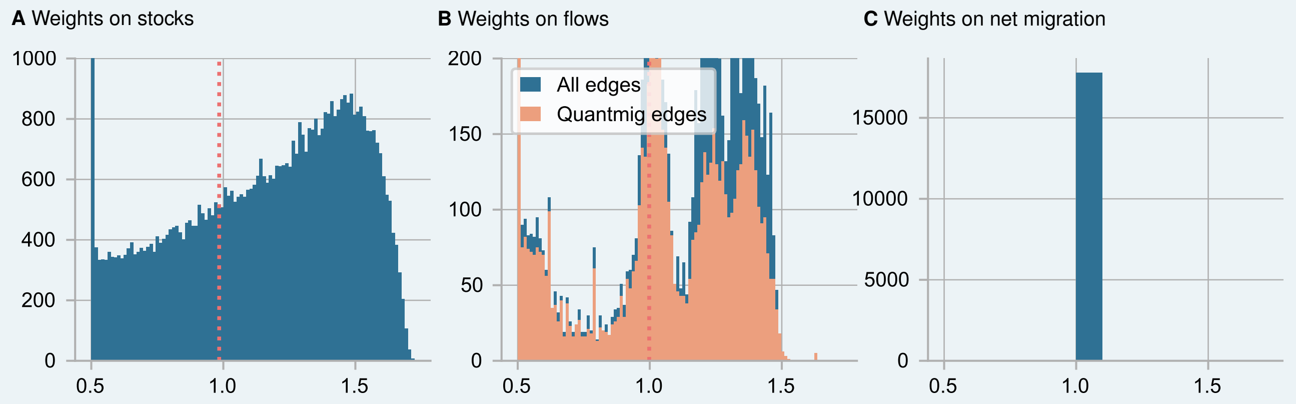

Figure 4: Figure S5 as in the manuscript, with the weights only on the QuantMig values shown in panel **B**.

Comments on code

1. *The checklists are very clear and diligently prepared. There are no ethical issues and all statistical reporting is state of the art. I have sampled content, including the code, from the GitHub repository and found it very well prepared and documented—the deposit itself is in many ways exemplary. My only suggestion here (for later—with no bearing on the publication of the paper) to ensure preservation would be to duplicate the current version of the code on Zenodo, as a version of record, which would be independent from a private GitHub account.*

   We will set up automatic depositing of Github releases to Zenodo once all revisions have been accepted; since the manuscript was submitted, we have corrected some bugs and also made improvements to the efficiency of the training code. The data has also been moved out of the Github repository to huggingface.

Reviewers 4 & 5

The quality of the visualizations is excellent. However, some figures are challenging to digest due to unclear captions, labels, or references.

1. *For example, Figure 1A's title could replace Registration-based measurements to specify that it is specifically for flows from Poland to Germany. This also affects Figure 1B.*

   This has been amended for Figs. 1**A** and 1**B**.

2. *Specify the unit of measurement on the axes for Figure 2B/C (assuming this is millions of people)*

   We have added a unit to 2**B**—indeed it is millions; in 2**C**, the units were given by the scientific scale in the top left, but we have changed this: all flows in Fig. 2 now indicate flows in millions. We have similarly standardised the flows in other figures to more clearly indicate the scale; where scales span several orders of magnitude, using different scales for individual panels is unavoidable (for the sake of legibility).

3. *It is unclear what the size of each region's band means in Figure 2B. Is this the estimated population in each region and the arrows represent the flow?*

   The arrowhead indicates the direction of the estimated migration flow. The width of the arrow at its base indicates the size of the migrant flows. Numbers on the outer section axis indicate the size of the migration flows in millions. We have added this description to the Figure caption.

4. *We suggest updating all written references in the manuscript to specify the figure number along with the panel (i.e., instead of "as seen in Panel B", specify "as seen in Fig X.B").*

   We have updated this accordingly.

5. *Figure 6 was challenging to understand. The titles in Panel A should specify the data source (assuming DESA?) and a color legend should be included to understand the gradient from green -> yellow -> red.*

   We have added a colour legend – the colour simply emphasises the relative error. The source of training stock data is UN DESA as described in the Methods section; this is the only source of stock data used throughout the article, so there should be no ambiguity as to the source of the stock data.

6. *The underlying data used matches the standard of quality of what other migration estimation studies use, and comes from diverse locations (Facebook, labor stats, etc.). However, some descriptions of how much each data source is used when making up the target data would be useful.*

   We have added a plot (fig. S6) to the SI, which includes the size of each dataset and its relative size in the target data set (see also Q22).

7. *Overall, conclusions on the migration modeling could be reliable, as authors either highlight how close the estimates are to the data source or the QuantMig estimations, or they discuss a few examples of where the QuantMig estimates and their estimates differ with some justifications. However, these comparisons were not presented on a global scale (or all the countries estimated in QuantMig). The discussion on the important features based on their model is great, but it would also be interesting to know which features were the -least- important for the knowledge insight. Some discussion of this was included in the elasticity section of the methods, but including a brief statement in the main conclusions would be useful.*

   We thank the reviewer for this suggestion. We note that QuantMig was developed specifically for Europe, so direct global comparisons are not feasible. In Figure 6 we present cross-validation results against all QuantMig flows (alongside other test flow data) in panel A, and against DESA stocks for the global scale in panel B. In panel C we compare the uncertainty of all QuantMig flows and UN DESA stocks with our estimated uncertainty. We have clarified this in the caption and text to make the scope of each comparison explicit. In addition, we have added a brief statement in the Discussion noting which features were least important in our model, complementing the elasticity discussion in the Testing and Validation section.

8. *Why was cross validation not used to evaluate over all flow corridors? How did you determine what flow corridors to train vs test? Were they spatially stratified were they temporarily stratified? There is a vast literature on the undesirable sensitivity to a single train / test split as was done here. Given the possible strong geographic and temporal differences the choice of train and test has to be done very carefully and given that it is hard to get right, it needs to be repeated (randomized) multiple times for the final reported metrics of performance of the model to be representative. Ideally the authors should report metrics over repeated train/test splits or just do 5-fold cross validation. If not, the authors should add a strong justification for why cross validation or repeated train/test splitting was not utilized with more details on the representation in the training/test data (making sure the test set was representative).*

   We thank the reviewers for pointing this out; as suggested, we have performed a 5-fold cross-validation, and all test performance statistics are now reported across the folds. The elasticity was re-calculated across the ensemble. We have amended the text accordingly, and have also included an additional plot to the SI (Fig. S23), showing each tested model's cross-fold validation performance.

9. *It would be useful to move the justification for why correlation/MRE were used to the start of the Testing and Validation section, as it wasn't justified until after some results were already discussed and in the Supplementary Information.*
   Done.

10. *The choice of which years/regions to highlight in Figures 3A and 4A should be justified.*

    See Reviewer 1 & 2, Q5.

11. *The authors should comment on whether the migration insights derived from your model that are mapped in these plots can be unveiled through the existing approaches. What is the new knowledge your method unveils here (that with the raw training data or with prior approaches would either be unavailable or the models would have pointed to a different insight about migration)? Similarly, on page 8 before the Testing and Validation section, we believe adding an argument for how the uncertainties derived from the QuantMig study differ from the estimates derived for the same countries in the proposed approach. Specifically detailing new conclusions and compare/contrasting the knowledge insights between the two methods.*

    We agree that it is important to clarify the novel insights our approach provides. We have added text in the last paragraph of the Discussion to highlight how our estimation framework goes beyond what can be gleaned from raw training data or prior approaches. Specifically, it integrates multiple data sources, quantifies uncertainty, captures complex nonlinearities and threshold effects, and provides a finer temporal resolution than previous global migration estimates, all within a unified framework. We have also sought to restrict our contributions to migration estimation rather than forecasting or causal explanation, which lie beyond the scope of our analysis—though we hope in the future that our estimates can nevertheless make an important contribution to both.

    In addition, we have expanded the Methods section to explain how our uncertainty quantification differs from prior work. Previous approaches, such as IMEM and its successor QuantMig, focused on European flows, reconciling definitional mismatches and measurement error with rich metadata and expert opinion. By contrast, our framework is designed for the global setting, where estimates must cover both stocks and flows simultaneously across many countries with limited metadata. We explicitly propagate uncertainty in migrant stocks through ensemble training and initial stock sampling, and incorporate covariate uncertainty. The differences in approaches are motivated by the distinct goals and data environments: our uncertainty quantification is tailored to the global scale, whereas IMEM/QuantMig were designed for the European context. As a result, it is prohibitively complex to identify precise causes for differences in the uncertainty estimates between the two approaches, beyond the comparisons already presented in Figures 3 and 6 and in response to Reviewer 3, Point 5.

12. *The strongest weakness of the study is the contradiction with the authors' claiming innovation on bringing a sophisticated machine learning (ML) approach (recurrent neural networks) to the problem of predicting migrations vs. not actually including a deeper literature review on ML-based approaches to human migration and choosing to benchmark only against a single paper / approach QuantMig. The QuantMig method, which the authors use as their baseline, does not cite existing technical approaches for this predictive problem or have a proper literature review and hence it is difficult to understand why QuantMig is the best baseline for the approach presented here. Therefore, this submitted manuscript is missing a proper literature review, especially as it relates to proposing a machine learning model (recurrent Neural Networks) as the main method's contribution, and hence, prior ML approaches to migration prediction should be addressed.*

    We thank the reviewer for this important observation. We had aimed to situate our contribution in the global migration setting, where there have been no proposed ML approaches. We have clarified this point in the Introduction section. However, we agree that the paper is strengthened by situating our recurrent neural network approach within the broader literature on ML-based mobility and migration research. We have expanded the Introduction and Methods sections to include a more detailed review of prior ML approaches to estimating mobility and migration, including applications to commuting and trip modelling, refugee forecasting, asylum applications, and internal migration. We have also added references to prior Bayesian approaches to estimating European international migration in the Methods section.

We would like to clarify that we do not treat QuantMig as a methodological benchmark. Rather, QuantMig is one of several target datasets used in our estimation framework, alongside other sources such as global estimates based on Facebook data, national statistical office data (e.g., New Zealand, Sweden, Finland), selected net migration datasets, and UN DESA migrant stock figures. QuantMig is included because it represents a recent large-scale European project that produces annual migration flow estimates, which we use as one of several reference points to inform our migrant flow and stock estimates in the region.

13. *There is a brief mention of "gravity models", but no elaboration beyond a citation to a single* ***discussion*** *paper. There are many studies that build statistical / ML models to estimate migration, some purely for migration research, others in the context of studying migration-based questions, such as forced migration under, for example, sea level rise under climate change, and those (often machine learning) models apply to the general migration estimation problem. Here are several papers that all proposed ML-based approaches relevant to modeling human migrations and the broader topic of human origin-destination flows modeling, any of which would be just as valid if not better baseline for the presented approach than QuantMig (many of these approaches can provide uncertainty estimates in one for or another, either directly through the model or from bootstrapping train/test splits etc)*

    - *Hauer, M. Migration induced by sea-level rise could reshape the US population landscape. Nature Clim Change 7, 321–325 (2017). https://doi.org/10.1038/nclimate3271,*
    - *Robinson C, Dilkina B, Moreno-Cruz J (2020) Modeling migration patterns in the USA under sea level rise. PLoS ONE 15(1): e0227436. https://doi.org/10.1371/journal.pone.0227436*
    - *Simini, F., Barlacchi, G., Luca, M. et al. A Deep Gravity model for mobility flows generation. Nat Commun 12, 6576 (2021). https://doi.org/10.1038/s41467-021-26752-4*
    - *Can Rong, Jingtao Ding, and Yong Li. 2024. An Interdisciplinary Survey on Origin-destination Flows Modeling: Theory and Techniques. ACM Comput. Surv. 57, 1, Article 4 (January 2025), 49 pages. https://doi.org/10.1145/3682058*

    See the response to the previous point for the location of our expanded discussion of the recent advances in the development of statistical/ML models for mobility and migration research. This includes these suggested citations, as well as several others.

    We believe these studies are relevant, but they are not directly applicable as baselines for our work. Our focus is on estimating annual global migration flows and stocks simultaneously to provide a plausible demographic account, where the response variable itself is uncertain and must be constructed from a variety of migration data sources. The direct benchmarks are the existing estimates of global migration that we utilise throughout the paper. By contrast, the cited studies generally assume a well-defined response variable and concentrate on explanatory covariates (e.g., climate shocks, mobility traces). None of these papers proposes their methods as candidates for global migration estimation or for integrating migration data sources. Moreover, many rely on covariates that are not robust predictors of global migration patterns and trends, such as localized climate variables. For these reasons, we treat such studies as part of the broader ML literature on mobility and migration, but not as methodological baselines for our global estimation framework.

    We address the possibility of the QuantMig approach as a benchmark in our response to the point below.

14. *The QuantMig approach benchmarked here was not even re-applied to the full global dataset used in this study, but only the reported estimates for the 30 countries that the QuantMig paper focused on were compared. Hence, from a methodological perspective, there is no apples-to-apples comparison on the same dataset and label (and train/test splits) used for the proposed approach here to other ML-based methods applied to the exact same data. We request that such a methodological benchmark is appropriate to be provided to establish the relative strength of the new method. This does not take away from the contribution of compiling the global migration dataset and variables that the authors provided*

here, but still leaves open to questions whether the recurrent Neural Network method proposed is significantly better than other ML-based approaches proposed in the past.

The reviewer is under the impression that the QuantMig framework is a methodological approach that can be applied to produce flows for arbitrary corridors, but that we only chose to use the European flows, rather than generate (or benchmark against) a global version. However, the QuantMig framework was a long-term, ERC-funded project which extended work from another large-scale project (IMEM) that painstakingly harmonised *European* flow data through a Bayesian framework, primarily by collecting expert opinions on the timing criteria, coverage and accuracy of international migration flow statistics in European countries, which were then transformed into informative prior distributions for a measurement model within their frameworks. In the introduction, we wrote that "the QuantMig project used a Bayesian framework to estimate bilateral migration flows for 30 European countries from 2009 to 2019". This is misleading, and we have amended the passage accordingly to avoid suggesting that the QuantMig project offers a general method that could easily be reproduced. In reality, it is a dataset that cannot be extended or reproduced without extensive work, certainly beyond the scope of this article. Even then, we strongly suspect the IMEM/Quantmig framework will struggle in the global data environment due to the dearth of migration flow statistics available to monitor many major migration corridors between developing countries.

15. *On page 8, you make a reference to the target data. What is the target data?*

    The target is introduced on page 3 (emphasised), and then again in more detail in the Methods section (under 'Target data'). It comprises all the target values (stocks, flows, net migration values) that are used to construct the loss function eq. (9).

16. *You made a reference to Figure 5D on page 8, but we believe this should be 5C.* Corrected.

17. *On page 8 before Testing and Validation, we believe adding an argument for your presentation of the uncertainty.* See Reviewer 3, Point 12.

18. *Figure 6A - Is the first chart DESA Stocks? Is this a training/testing dataset?* Yes, these are DESA stocks—all stock data used in the work are UN DESA stocks. The stocks are all training data, only flows are used for testing. See also Q5.

19. *Page 7 appears to be missing a reference to citation 34, when mentioning the WPP.* Added.

20. *We recommend reminding the readers what μ is on page 7, as it is introduced very early on in the paper.* We believe this should be clear from the text immediately above the formula, which discusses net migration; μ also appears on the LHS of the equation, meaning the equation can be taken as a definition.

21. *We recommend adding some sort of indicator on the size of your datasets that you are using.* We have added a plot (fig. S6) to the SI, showing the size of each dataset and its relative size in the target data set (see also Q6).

22. *In the introduction, it is unclear how the digital trace data from Facebook is calculated and ultimately measures migration. Additionally, what are the biases of using this data? Communicating that briefly, as mentioned with the labour migration and UN DESA data sources, would be a good addition.* We have included some additional description in the Introduction on the Facebook flow estimates.

23. *Page 14 says there are 4 sources, but then only lists 3 items in the list.* Corrected: in fact this should be five—three national statistics (Sweden, New Zealand, Finland), QuantMig, and Facebook data.

References

1. De Haas, H. A theory of migration: the aspirations-capabilities framework. *Comparative migration studies* **9,** 8 (2021).
2. Castles, S. & Miller, M. J. *The Age of Migration: International Population Movements in the Modern World* 4th (Palgrave Macmillan, Basingstoke, 2009).
3. Salt, J. Contemporary trends in international migration study. *International Migration* **25,** 241–251 (1987).
4. Gerland, P. *et al.* World population stabilization unlikely this century. eng. *Science (American Association for the Advancement of Science)* **346,** 234–237. ISSN: 0036-8075 (2014).
5. Chi, G., Abel, G. J., Johnston, D., Giraudy, E. & Bailey, M. Measuring global migration flows using online data. *Proceedings of the National Academy of Sciences* **122,** e2409418122. eprint: https://www.pnas.org/doi/pdf/10.1073/pnas.2409418122. https://www.pnas.org/doi/abs/10.1073/pnas.2409418122 (2025).
6. Azose, J. J. & Raftery, A. E. Estimation of emigration, return migration, and transit migration between all pairs of countries. *Proceedings of the National Academy of Sciences* **116,** 116–122. ISSN: 1091-6490. http://dx.doi.org/10.1073/pnas.1722334116 (Dec. 2018).
7. Welch, N. G. & Raftery, A. E. Probabilistic forecasts of international bilateral migration flows. *Proceedings of the National Academy of Sciences* **119,** e2203822119. eprint: https://www.pnas.org/doi/pdf/10.1073/pnas.2203822119. https://www.pnas.org/doi/abs/10.1073/pnas.2203822119 (2022).

Response to reviewers

Deep learning four decades of human migration

Thomas Gaskin[1, 2], Guy J. Abel[3]

[1] Department of Methodology, London School of Economics and Political Science; [2] Department of Applied Mathematics and Theoretical Physics, University of Cambridge; [3] Department of Sociology, University of Hong Kong

Note: We have made two notational changes: the net migration is now denoted by M, and the default index notation is now T_{bij} with b for country of birth, i for origin, and j for destination. This aligns the notation with that of much of the literature in the field, and allows us to use the standard F_{ij} notation for the flows from origin country i to destination country j. For legibility reasons, we have not marked these changes in the manuscript.

Reviewers 1 & 2

1. *We are unsure about the originality of the findings. What the paper does well is to illustrate and confirm major findings previously known about specific global migration flows (see pp. 8-10 on testing and validation), but its empirical findings (to the extent that the paper contains any substantive insights) are neither original nor significant.*

   We appreciate this concern and agree that many of the broad migration patterns we report—such as the prominence of certain corridors or the persistence of regional migration systems—are consistent with prior scholarship. However, the originality of our contribution lies not in rediscovering these well-established facts, but in providing the first globally consistent annual flow estimates that enable such validation. By moving beyond five-year migration estimates, our approach enables analyses that were previously infeasible:

   - It captures short-term shocks and surges that are smoothed or hidden in existing five-year flow transition estimates.
   - It allows researchers to link migration flows to annually reported drivers (e.g., labour market statistics, conflict indicators, policy reforms).
   - It provides a framework for comparative and causal analysis across regions.
   - It allows demographers to utilise annual migration measures within single-year cohort-component models.
   - It helps address existing problems on the receiving-country bias in migration research by extending annual coverage to countries without data collection systems to monitor migration flows.

   In this sense, the empirical findings serve a dual purpose: they validate the robustness of our estimates against known migration patterns, and they demonstrate the new analytical possibilities opened by annual flow data. We believe this methodological advance constitutes a substantive and original contribution to the study of the global migration system.

2. *The current draft remains under-theorized and under-motivated. The paper lacks a clear intellectual motivation. Here are a few questions: What is the scale of the "problem" (i.e., inadequate annual data coverage) that motivates this analysis? Does the proposed approach and solution improve on the existing data? An articulation of the scale of the problem and why it needs to be solved will help readers grasp the significance of this paper and more effectively motivate the entire analysis, beyond just "improving resolution." To use an analogy, bigger maps are not necessarily better tools. The paper might be falling into the "big-data trap," where more data is "assumed" to be always better.For example, in response to our prior comment, the authors wrote: "The challenges are very real, and we have added some further explanation and references to point readers towards a more thorough documentation of the problem. Figure 1 is merely intended to illustrate the situation, and it is beyond the scope of our manuscript to conclusively demonstrate that global migration data is inconsistent, difficult to measure reliably, and in need of harmonization." This is precisely the point that we raised: how large is this data coverage problem? And how far does this new approach help improve this situation? What new and original insights do we gain about global migration patterns using this new approach and improved dataset?*

   We appreciate the reviewer's encouragement to clarify the intellectual motivation. We have now explicitly articulated our motivation for estimating annual migration patterns and why they constitute a significant step forward relative to the existing five-year estimates in the second paragraph of the introduction. This includes a new Fig. 1A, which illustrates the lack of reported annual flow data at the global level. Based on available collections of reported international migration flows, more than 85% of migration corridor-years between 1990 and 2020 have no data. We have also revised other parts of the introduction to further emphasise the motivations of our work.

3. *Throughout the paper, the authors cite many data points that "confirm" what we already know about various flows, but, to the best of our reading, no new findings emerge from this approach. We need a couple of good examples of where the current data has really failed researchers, such that this sophisticated analysis (set of estimates) is warranted. Perhaps the authors can provide a few examples in which the new dataset fills a previously unknown gap or contradicts widely expected findings. That will help illustrate the value of this approach.*

   As we note in the new paragraph in the introduction and allude to above, there are several major limitations in the currently reported annual flow data and in global migration estimates based on five-year intervals. Note that due to space constraints and our desire to provide a concise overview rather than lengthy elaboration, we summarise only these challenges and benefits in the revised introduction. However, we would like to highlight three areas where existing data have failed researchers and where our dataset provides new insights:

   - Existing annual migration flow estimates are almost exclusively available for high-income Western countries, leaving the Global South largely unrepresented. Our dataset addresses this failure by providing globally consistent annual estimates, including for countries without administrative systems to monitor international migration flows. For example, we capture the year-to-year dynamics from South Asia to the Middle East in response to economic fluctuations and policy reforms, and show how African regional migration systems—such as movements within West Africa—evolve annually in response to policy changes and conflict, filling a gap where five-year transition data obscure short-term changes and existing annual data are unavailable.
   - With only six five-year observations over three decades, researchers cannot robustly distinguish long-term trends from short-term shocks, nor align migration flows with contemporaneous drivers such as conflict onset, economic crises, or policy reforms. The lack of annual data, hence, constrains comparative and causal analysis of the global migration systems. However, our annual estimates enable causal designs for analyses to study and compare migration patterns over time that were previously impossible along the vast majority of migration corridors.
   - Population projection models require demographic measures (births, deaths, migration) to match the interval length of the age decomposition. When age groups are defined in five-year intervals, five-year migration

flows can be used alongside five-year fertility and mortality rates. But when age groups are defined annually, as in single-year cohort-component models, migration inputs must also be annual. This restriction prohibits the use of existing five-year migration flow estimates in single-year models with annual population, birth and death data provided for all countries by the United Nations. In what is known as the "one-year/five-year problem," five-year migration data cannot simply be divided by five to obtain annual flows, because migration is unevenly distributed across years and often involves repeat, onward, or return movements. Periods of crisis, policy change, or economic fluctuation frequently concentrate migration in particular years, making the relationship between migration volume and interval length inherently non-linear and potentially unique to each migration corridor [1]. As a result, current single-year projections, such as those produced by the UN World Population Prospects, rely on net migration figures, which collapse immigration and emigration into a single balance and obscure underlying dynamics. These simplified measures are known to produce less accurate projections than more nuanced bi-regional or multi-regional population projection models [2]. By providing annual bilateral flows, our dataset resolves this mismatch, enabling single-year cohort-component models to incorporate immigration and emigration totals, rates—or even full bilateral matrices alongside births and deaths. This has the potential to improve the accuracy and realism of population projections, particularly for countries where net migration masks offsetting inflows and outflows, or where forecasting and scenario development require a clearer picture of directional movements.

4. *In this version, many of the authors' examples reflect what is widely known. For instance, it is not surprising that one of the most significant migration events in the past 30 years is driven by the conflict in the DRC, nor that the main migration routes are from the US-Mexico border and from Asia to the Middle East. Likewise, we know that migration has been steadily increasing.*

   We agree that the broad events we highlight—such as displacement from the DRC, migration along the US-Mexico corridor, or flows from Asia to the Middle East—are well-established in the literature. Our aim was not to claim novelty in identifying these events, but to demonstrate how annual flow estimates allow us to analyse them in greater depth and with new comparative precision. For example, our estimates make it possible to trace how conflict in the DRC reshaped flows to particular destinations over time, and to compare these dynamics to other conflict-driven shocks elsewhere. Similarly, we can examine how policy interventions at the US-Mexico border altered flows year-by-year and contrast these effects with policy-driven changes, for example, in the opening up of the European Union or in migration from North Africa.

   Importantly, because our dataset provides annual observations, it enables researchers to move beyond describing *where and when* migration occurs to investigating *why* flows respond to particular shocks or policies. This capacity for causal inference—linking annual flows to contemporaneous drivers such as economic change, policy reform or conflict—is precisely what distinguishes our contribution from prior work based on five-year migration data. The current coarse resolution of migration flow estimates restricts causal inference: with only six five-year observations across a thirty-year period, it is difficult to identify how specific shocks or policy interventions shape migration flows.

5. *The authors need to make a stronger case for the problem and the utility of their method. This is the main contribution, and it is mostly hidden by all the technical discussion (which is, of course, important) that is crowding out the argument. This may mean including a version of Figure 2 in the response memo in the main manuscript.*

   We appreciate this comment and agree that the manuscript should more clearly foreground the problem and the utility of our method. As noted in the responses above, we have strengthened the introduction in the revised version to highlight the limitations of existing migration flow data and the analytical opportunities created by our annual estimates, and have updated Figure 1 to include an overview of the global scale of the issue of missing data. We believe these revisions make the problem and the value of our solution much more visible to readers.

6. *How do unauthorized migrants shape these estimates and findings? How does the model account for them? What are*

the key assumptions made about them? These questions cut to the core of the models and these estimates, including the paper's intellectual motivation (better data, more detail, and better descriptions). For example, when estimates and counts of the Mexican immigrant inflow in the US exceed the count of Mexican migrant outflow from Mexico each year, both measures could be accurate if the differential/discrepancy is due to undocumented migrants from Mexico to the US. Therefore, the discrepancy in records/sources reflects accurate counting, not errors in the counts/estimates. There are several scenarios here. Both Mexico and the US undercount the undocumented, given the more clandestine movements of this migration flow. Mexico might accurately count them, but the US undercounts them (because they are unauthorized). Mexico might not keep an official count of this group of migrants, yet their numbers appear in the US Census, which does include a count of the undocumented population. Etc. Each scenario will affect the estimates used as input to the deep learning models. Moreover, the assumptions about any observed discrepancies matter in how we treat these datapoints (as valid or invalid).

We agree that discrepancies such as those highlighted in the US–Mexico case are real and important. They are similar (in a statistical measurement sense) to the discrepancies we discuss in the paper for German–Polish flows, where differences arise because origin and destination sources apply different timing criteria in their definitions of migration. In the US–Mexico case, however, the key issue is not timing but coverage: destination data often includes unauthorised migrants, while origin data may not. This asymmetry can generate inflow–outflow differentials that reflect genuine differences in coverage rather than errors.

Our model does not attempt to resolve these discrepancies. Instead, the deep learning framework is trained on target data that all adopt a common definition of migration, in line with UN recommendations. We are very particular about our choice of target values to ensure that we do not include data sources with significantly different definitions of migration flows.

As we do not include any US or Mexican bilateral flow data as target values, unauthorised migrants are only implicitly included to the extent that they appear in the underlying target stock data (for example, census counts in destination countries), estimates based on the Facebook paper, or in net migration calculations from national demographic accounting balances.

7. *The authors need to be more precise and draw a distinction between migrant "stocks" and "flows." For example, the abstract mentions both "migration flows and stocks between 230 countries and regions." Does this approach improve estimates for both stocks and flows? Or only flows? These two measures capture different aspects of migration. Our reading is that the paper addresses the "flows", but not the "stocks".*

   We thank the reviewer for raising the important distinction between migrant stocks and flows. Our framework estimates both: migrant stocks are incorporated separately in the model as covariates and targets, with accumulated flows contributing to migrant populations (stocks). The evolution of stocks is therefore tightly linked to flow dynamics and is described in Equation (2). However, the paper's substantive focus is on flows, which provide far greater utility for understanding migration dynamics. To avoid confusion, we have revised the abstract and introduction to emphasise flows as the central contribution, removing phrasing that previously suggested equal attention to both stocks and flows.

8. *The authors could be more selective about what visualizations are critical to make their points. In this version, we are still presented with lots of information that is not always important or well-explained.*

   We thank the reviewer for raising the issue of selectivity in visualisations. Because the comment did not specify which figures might be considered less critical, we carefully revisited all figures to assess their contribution. We concluded that each figure highlights a distinct aspect of the study: the motivation (Fig. 1), global results (Fig. 2), a data-rich region (Fig. 3), a data-poor region (Fig. 4), comparison with available net migration data (Fig. 5), and model performance (Fig. 6). Each is therefore necessary to support the paper's arguments. We hope this resolves

the concern while retaining the transparency and completeness of our presentation, though we would be happy to reconsider the point if further advice is provided.

9. *We are not sure what to make of their point in the memo: that predictions fare worse with more data. This raises questions for us about the certainty of their South-South predictions, which are then trained on a single data source. Couldn't these be really off?*

   We would like to clarify that our statement in the memo—that predictions fare worse with more data—was not intended to suggest that the model deteriorates as data availability increases. Rather, it reflects that in data-rich contexts, predictions are benchmarked against multiple target data points, thereby naturally exposing discrepancies. In contrast, in data-poor contexts (such as many South–South corridors), the model is trained on and evaluated against limited benchmarks.

Reviewer 3

1. *In the new text, both in the article and SI, the use of upper caps in "QuantMig" needs unifying.* Amended.

Reviewers 4 & 5

1. *Regarding Fig. 2: does the overall size of each region's band (not the base of the arrow, but the dark colored rounded sections on the outside) represent something significant like the population of the region? If so, this could still be further clarified in the figure caption.* We thank the reviewers for their attention to this matter; indeed, we had failed to realise this was unclear during the first round of revisions. The section bands show the total flow volume in 2023, allowing for easier comparison of flows over the years. We have added a clarifying note to the caption.

2. *There is one instance where the panel language remains at the bottom of the left column on page 5, but it is made clearer by adding '3B'. To maintain consistency, this could be changed to 'Figure 3B'.* Amended.

3. *We believe including the data source in the title of Fig. 6 would still make the text more readable.* Amended

4. *Page 23: "The optimal offset $b_{ij} \in \mathbb{R}$ of the initial stock ... " should be ρ_{ij} instead of b_{ij}.* Amended.

5. *We thank the authors for explaining the QuantMig framework in more detail. We believe the authors' edits in the introduction could be further improved by including a version of their last sentence in the author rebuttal into the paper to make it clear that QuantMig is not easily transferable to other migration corridors.* Added.

6. *On page 8, you make a reference to the target data. We believe editing the text of the main text to match the author rebuttal text, specifically "comprises the data (stocks, flows, net migration values) that are used to construct the loss function" would improve readability and understanding from a multi-disciplinary audience.*

   We thank the reviews for their attention to this matter; the phrase "comprises the data (stocks, flows, net migration values) that are used to construct the loss function" is already used word-for-word in our explanation of the target data in the introduction. In view of the space constraints, we will adhere to the current format: giving an explanation of the target data in brief in the introduction, and in detail in the Methods section.

7. *Regarding uncertainty quantification: we note that we believe the text in the Appendix (Page 22) is still not precise enough to be able to fully reproduce the approach (e.g., it only says for the covariates uncertainty to "draw n = 100 samples of the initial stocks to estimate overall uncertainty"). For example, in the code section titles "Example: uncertainty quantification by sampling inputs" provided by the authors, it says "Here we illustrate the principle, by using a standard deviation of 5% on all covariates except the stock data, where we use 10%. Binary covariates are not sampled." The*

authors add this new text "We develop our uncertainty quantification approach at the global scale by hand-picking high-quality migration flow sources with similar definitions and adjusting the loss function to account for differences in data uncertainty, while propagating migration stock uncertainty through variation in initial stock sampling and our ensemble training." We note that picking high-quality sources and adjusting the loss are NOT strategies for uncertainty quantification; they are strategies for minimizing uncertainty in the model. Hence, we advise authors to rephrase the sentence.

We thank the reviewers for spotting this—the paragraph in question was both a repetition and highly confusing, and we have largely removed it. The uncertainty quantification approach as outlined in the appendix should now be clear: train 15 neural networks in parallel, sample $n = 100$ initial stock values for each, and propagate the uncertainty through each ensemble member, giving a total of $n = 1500$ samples. The code repository gives an example for additionally assuming an error on the other covariates, for which we have designed the code, but now explicitly mentions that this is not done when generating the final estimates as it would require making assumptions on the quality of each input covariate.

8. *We still recommend reminding the readers what μ is on page 7, as it is introduced very early on in the paper.* Amended.

9. *Some parts of the code (Evaluation script) are named "Example ..." or "Demo ...", which seems to suggest they are not the exact procedure used for the analysis shown in the manuscript, but a simplified or reduced demo version. If that is the case, the documentation of those pieces of code should include precise instructions for setting up the code to reproduce the results presented in the paper exactly.*

   We greatly appreciate the feedback, and have re-structured the notebook such that it is more apparent which parts reproduce the plots and which parts are intended only for demonstration purposes. One of the journal requirements is that we provide a simplified code demo, but we agree that the previous notebook structure was haphazard, and hope it is improved now.

Editorial comments

1. Word count: approx. 4000 words (not counting abstract, references, and Methods). We assessed this before the changes made to the manuscript, as the current tracking makes it impossible to accurately count words.

2. Figure count: 6 figures.

3. Figure legends: we have added statistical information to all figures.

4. Figure format: all figures are provided as vector graphics.

References

1. Kitsul, P. & Philipov, D. *The One-Year–Five-Year Migration Problem* tech. rep. WP-80-81 (International Institute for Applied Systems Analysis, 1980).
2. Rogers, A. Requiem for the Net Migrant. *Geographical Analysis* **22,** 283–300 (1990).
